# HERPUD1 promotes ovarian cancer cell survival by sustaining autophagy and inhibit apoptosis via PI3K/AKT/mTOR and p38 MAPK signaling pathways

**DOI:** 10.1186/s12885-022-10248-5

**Published:** 2022-12-21

**Authors:** Xin Nie, Dawo Liu, Mingjun Zheng, Xiao Li, Ouxuan Liu, Qian Guo, Liancheng Zhu, Bei Lin

**Affiliations:** 1 Department of Obstetrics and Gynecology, Shengjing Hospital of China Medical University, 36 Sanhao Road, Heping District, Shenyang, 110004 China; 2 Key Laboratory of Maternal-Fetal Medicine of Liaoning Province, Key Laboratory of Obstetrics and Gynecology of Higher Education of Liaoning Province, Shenyang, China; 3 Department of Obstetrics and Gynecology, University Hospital, LMU Munich, Munich, Germany

**Keywords:** HERPUD1, Ovarian cancer, PI3K, MAPK, Endoplasmic reticulum stress

## Abstract

HERPUD1 is an important early marker of endoplasmic reticulum stress (ERS) and is involved in the ubiquitination and degradation of several unfolded proteins. However, its role in tumorigenesis is seldom studied, and its role in ovarian cancer is unclear. Lewis y antigen is a tumor-associated sugar antigen that acts as an ‘antenna’ on the cell surface to receive signals from both inside and outside the cell. We previously reported that Lewis y can promote ovarian cancer by promoting autophagy and inhibiting apoptosis. In this study, we detect the expression of HERPUD1 and Lewis y antigens in 119 different ovarian cancer tissues, determine their relationship with clinicopathological parameters, analyze the correlation between these two proteins, and explore the related cancer-promoting mechanisms through MTT, flow cytometry, western blotting, and bioinformatics. HERPUD1 is highly expressed in ovarian cancer, especially in the early stage, and the expression of HERPUD1 and Lewis y antigen was positively correlated. After overexpression of Lewis y antigen, the expression level of HERPUD1 increased. Gene Ontology (GO) and Kyoto Encyclopedia of Genes and Genomes pathways (KEGG) analysis showed that HERPUD1 and its related genes are enriched in regulating immunity, endoplasmic reticulum stress, ubiquitin-dependent degradation, ERS-induced apoptosis, and other key signaling pathways. We also clarified the HERPUD1 network of kinases, microRNA and transcription factor targets, and the impact of HERPUD1 mutations on prognosis. In addition, HERPUD1 promotes the proliferation of ovarian cancer cells, inhibits apoptosis, affects the cell cycle, promotes the occurrence of autophagy, and inhibits EMT and PI3K/AKT/mTOR and p38MAPK pathways. Overall, HERPUD1, regulated by the expression of tumor-associated protein Lewis y, promotes cell survival in the early stages of tumors, suggesting that HERPUD1 may play an important role in the development of ovarian cancer.

**Supplementary Information:**

The online version contains supplementary material available at 10.1186/s12885-022-10248-5.

## Introduction

Epithelial ovarian carcinoma (EOC) has the highest mortality rate among gynecological malignancies, but the carcinogenic mechanism of EOC remains unclear [1]. Increasing evidence shows that endoplasmic reticulum stress (ERS) plays an important role in tumors. Cancer usually occurs in a microenvironment undergoing stress (such as abnormal tissue proliferation, chronic inflammation, injury, etc.), which may destroy the protein folding mechanism of the ER, leading to the accumulation of unfolded or misfolded proteins and disturbing the intracellular environment, thereby causing ERS [2, 3]. ERS can cause the ER to process the misfolded or unfolded proteins accumulated in the cavity of the reticulum in order to maintain normal cell function, but continuous excessive stress will cause endoplasmic reticulum function disorder and induce apoptosis. In order to reduce ERS and avoid cell apoptosis, unfolded protein response (UPR) is stimulated to maintain an effective protein folding environment [4–6]. ER-associated degradation (ERAD) is an important part of UPR, and identified the misfolded proteins (substrates of ERAD), recruited to the degradation complex, transported from the ER to the cytoplasm, and ubiquitinated; then they are transported to the 26S proteasome for hydrolysis [7–10].

The homocysteine-inducible ER protein with ubiquitin-like domain 1 (HERPUD1), also known as Herp, is a member of the ERAD degradation complex and participates in the ubiquitination and relocation of ERAD substrates [8, 10–12]. The early research of HERPUD1 mainly focused on diseases related to ERS regulated inflammation, including in the regulation of atherosclerotic inflammation and the degenerative changes to the “neuro-inflammatory” related proteins in Parkinson’s syndrome [13, 14]. A study found that HERPUD1 mRNA is significantly downregulated in metastatic prostate cancer, which effectively predicts the occurrence of tumor metastasis [15].

Glycosylation modification is one of the important ways of protein post-translational modification [16]. Lewis y antigen, a tumor-associated carbohydrate antigen, belongs to A, B, H, Lewis blood group families and is an oligosaccharide containing double fucosylation. Approximately 60–90% tissue express an increasing level of Lewis y antigen when they are undergoing cancerous transformation [17]. Alpha-1,2-fucosyltransferase is the key enzyme controlling the synthesis of Lewis y antigen. Our previous research results showed that Lewis y overexpression, induced by the transfection of α-1,2-focusyltransferase gene, promoted the malignant biological behaviors such as proliferation, adhesion, invasion, metastasis and drug resistance in ovarian cancer cells [18–27]. Using Human whole-genome oligonucleotide microarrays to detect the differences in gene expression profiles after transfection of α-1,2-focusyltransferase gene, we found that ER stress-related HSPA8 gene changes were at the forefront [28]. HSPA8 protects the proteome from stress, folds and transport of newly synthesized polypeptides, activates of proteolysis of misfolded proteins and formats and dissociates of protein complexes. We speculated that Lewis y is involved in the regulation of endoplasmic reticulum stress.

Currently, HERPUD1 is rarely reported in tumor researches. This study explored the role of HERPUD1 in ovarian cancer, its relationship with Lewis y, and related mechanisms through bioinformatics, clinical specimens, and cytology experiments, in order to provide new ideas for the research on ovarian cancer anticancer drugs.

## Materials and methods

### Specimen source and clinical data

Surgical paraffin-embedded pathology specimens were collected from 119 patients at the Department of Obstetrics and Gynecology, Shengjing Hospital affiliated to China Medical University, from 2006 to 2018. The pathological diagnosis of all histological sections was completed by a pathologist of Shengjing Hospital affiliated to China Medical University. They included 86 cases of ovarian epithelial cancer (malignant group), 12 cases of ovarian epithelial borderline tumors (borderline group), 9 cases of ovarian epithelial benign tumors (benign group), and 12 cases of normal ovarian tissue (normal group). All ovarian tissue samples were collected between 2008 and 2013 and embedded in paraffin by the staff of the obstetrics and gynecology surgery department of our hospital. The samples were re-confirmed by a pathologist for this study. Patients in the malignant group were of median age 58 years (range 36–79); borderline group, 46 years (range 30–66); benign group, 42 years (range 30–68); normal group, 44 years (35–62). There was no statistically significant difference in the age of each group (*P* > 0.05).

In the malignant group, two cases of ovarian cancer were well differentiated, 28 cases were moderately differentiated, and 56 cases were poorly differentiated. Staging was carried out according to the standards established by the International Federation of Obstetrics and Gynecology (FIGO) in 2009: 34 cases were stage I–II, and 52 cases were stage III–IV. These 86 cases underwent comprehensive exploration and staging of ovarian tumors in the early stage and cytoreductive surgery in the late stage. According to the metastasis of the pelvic and/or para-aortic lymph nodes, they were divided into: 46 cases without metastasis, 22 cases with metastasis, and 18 cases without dissection. None of the cases received radiotherapy or chemotherapy before surgery. Patient information is in Supplementary Table 1.

### Immunocytochemistry and immunohistochemistry

Cultured cells were seeded on coverslips and fixed with 4% paraformaldehyde, then stained with the SABC test kit (Wuhan Fine Biotech, Wuhan, China) according to the manufacturer’s instructions. Paraffin sections (5 μm) of patient tissue were routinely deparaffinized in water, and antigen heat retrieval was performed by boiling in citrate buffer. The tissue glass slides were immersed in buffer, and heated in the microwave oven for 10 minutes on high heat until the buffer boils to 100 degrees. After cooling for 10 minutes, the buffer is boiled again on high heat for 5 minutes. Staining was performed after cooling for 1 h to prevent tissue dissection. The expression of Lewis y and HERPUD1 was detected by the immunohistochemical streptavidin-peroxidase conjugation (SP) method (Zsgb Bio, Beijing, China). The working concentrations of primary antibodies against Lewis y antigen and HERPUD1 (both Abcam, Cambridge, MA, USA) were 1:50 and 1:200, respectively. Glioma tissue expressing HERPUD1 and breast cancer tissue expressing Lewis y were used as positive controls, and phosphate-buffered saline (PBS) was used as a negative control instead of incubating with primary antibody.

The result was judged as positive by the staining of cell membrane and cytoplasm and grouped according to the percentage of the visual field occupied by stained cells. The percentage of stained cells in the whole section was observed by the 200× optical microscope and scored. Scoring was as follows: 0 points for no positive cells, 1 point for 1–25% positive cells, 2 points for 26–50%, 3 for 51–75%, and 4 for 76–100%. Then the samples were scored according to the positive coloring intensity: 0 points, uncolored; 1, light yellow; 2, brown; and 3, dark brown. The scores of these two parameters were multiplied, and the final classifications were as follows: 0–2 points for negative (−), 3–4 for weak positive (+), 5–8 for moderate positive (++), and 9–12 for strong positive (+++). (−) is defined as negative expression, (+), (+ +), (+ + +) as positive expression. (−), (+) were defined as low expression, (+ +), (+ + +) were defined as high expression. Two pathologists judged the images independently to control for bias.

### Cell culture

Ovarian cancer cell lines (CAOV3, SKOV3, OVCAR3, ES-2) and normal ovarian epithelial cells (HOSEpiC) were purchased from Shanghai Cell Collection Center. Cells were cultured in RPMI 1640 medium (ThermoFisher, Waltham, MA, USA) containing 10% fetal bovine serum at 37 °C with 5% CO_2_ and saturated humidity.

### Cell transfection

CAOV3 and SKOV3 cells in logarithmic growth phase were digested by trypsin and seeded into 6-well plates. When cell confluency reached 50–70%, the siRNA fragments (GenePharma, Shanghai, China) was transfected into the cells using Lipofectamine 3000 Transfection Kit (ThermoFisher). Two siRNAs were generated and showed synergic effects on the knockdown of HERPUD1. The HERPUD1 siRNA sequence 1 (GenePharma) was as follows: sense, 5′- CCAGAGGACCAGAGGUUAATT-3′; antisense, 5′- UUAACCUCUGGUCCUCUGGTT-3′. The HERPUD1 siRNA sequence 2 (GenePharma) was as follows: sense, 5′-CCAGCCUGCCAAUCAGAAUTT-3′; antisense, 5′-AUUCUGAUUGGCAGGCUGGTT-3′. After 48 h transfection, the cells were collected to detect the interference effect and used in subsequent experiments. There were three replicates in each group, and the experiment was repeated three times.

Fucosyltransferase (FUT1) is a key enzyme in the synthesis of Lewis y. The pc-FUT1-GFP plasmid (Genechem, China) was transfected into the ovarian cancer cell line CAOV3 and SKOV3 to establish CAOV3-FUT1 and SKOV3-FUT1 cell lines with high Lewis y expression using Lipofectamine LTX combined with PLUS reagent (ThermoFisher) according to the manufacturer’s protocol. Stable transfected cells were selected in the presence of G418 (800 μg/mL for CAOV3 and 400 μg/mL for SKOV3).

### Real time-qPCR

Cells were collected and Trizol reagent was used to extract cell total RNA. The RT-PCR kit (TAKARA, Shiga, Japan) of the Super Script III First-Strand Synthesis System was used to reverse transcribe RNA into cDNA. The HERPUD1 primer sequences (Songon, Shanghai, China) were as follows: forward, 5′-TGGATTGGACCTATTCAGCAGC-3′; reverse, 5′-GCAGGTACATAACAACGGTGGC-3′. The amplification conditions were: denaturation at 95 °C for 30 s, annealing at 95 °C for 5 s, extension at 60 °C for 31 s, 40 cycles. Each reaction was repeated at least three times. After the reaction, the 2^−∆∆Ct^ method was used to calculate gene expression, and GAPDH was used as the internal reference for analysis.

### Western blot

Cells were collected and lysed in RIPA buffer to extract total protein. After separation by 10% SDS-PAGE, the protein was transferred to polyvinylidene difluoride membrane. The membrane was blocked with 5% skimmed milk at 37 °C for 1 h. Antibody hybridization was performed after cutting the PVDF membrane to an appropriate size. Then primary antibody was added and incubated overnight at 4 °C. The primary antibody details and dilutions in Supplementary Table 2. After washing with Tris-buffered saline with Triton X-100, the membrane was incubated with the appropriate secondary antibody (Zhongshan Jinqiao, 1:5000) for 2 h at room temperature. The protein bands were visualized by Image J 1.31v and normalized to the GAPDH protein expression level.

### MTT to detect cell proliferation

CAOV3 cells and SKOV3 cells were seeded in a 96-well plate at 2000 cells/well. Cells adhering to the plate after 6 h were recorded as “0 h”. MTT solution (20 μl of 5 mg/mL, Solarbio, Beijing, China) was added to each well and incubated for 4 h. The medium was aspirated from each well, 150 μl DMSO was added followed by shaking for 10 minutes, and then the absorbance was measured (490 nm). The experiment was repeated at 24, 48, 72, 96 h. Set 5 repeat holes and set zero adjustment holes. The experiment was repeated three times.

### Flow cytometry to detect cell apoptosis

Cells were collected, washed with PBS, and double-stained with Annexin-FITC/propidium iodide (PI) apoptosis reagent (Dojindo, Kumamoto, Japan). FITC and PI dyes (5 μL each) were added to each tube. Set up FITC single staining, PI single staining and blank control. The experiment was repeated three times.

### Flow cytometry to detect cell cycle

Cells were collected, washed with pre-cooled PBS, and fixed overnight with 70% ethanol at 4 °C. After washing the cells with PBS, 5 μl RNase A solution and 5 μl PI staining solution were added (Genechem, Shanghai, China). After staining at room temperature for 1 h, samples were loaded into a flow cytometer. The experiment was repeated three times.

### GEPIA analysis

GEPIA database (http://gepia.cancer-pku.cn) [29] is a website for dynamic analysis of gene expression profile data. We used the GEPIA database to analyze the expression level of HERPUD1 in different stages of ovarian cancer tissues. We used the “Stage plot” module of “Expression DIY” to further analyze the data, with the following screening conditions: (1) Gene: HERPUD1; (2) Datasets Selections: OV; (3) Log2FC Cutoff: 1; (4) *p*-value Cutoff: 0.05.

### LinkedOmics analysis

The LinkedOmics database (http://www.linkedomics.org) [30, 31] is a website for multi-omics and clinical database. We set the search conditions as follows: target tumor “OV”, target gene “HERPUD1”. We selected the relevant RNAseq data set, and used Pearson correlation coefficient to analyze the results. The differentially expressed genes related to HERPUD1 were presented in volcano map, heat map, and scatter plot formats. The LinkFinder module was used to enrich and analyze the signaling pathways and network regulation of differentially expressed genes. The grade standard was FDR < 0.05, and 500 simulations were performed.

### Metascape

Metascape (http://metascape.org) [32] is a gene annotation analysis tool that integrates multiple databases such as GO, KEGG, UniProt, and Drugbank. The differential expression of HERPUD1 was obtained through LinkedOmics, and we used Metascape to analyze the enrichment of the first 500 genes in the process and pathway under the limited conditions: *P* value < 0.01, minimum count 3, enrichment factor > 1.5. The use of the KEGG [33] database (www.kegg.jp/kegg/kegg1.html) has been approved by Kanehisa Laboratories.

### String analysis

String database (https://string-db.org/) [34] is an online database for searching protein interaction relationships, including direct physical interactions and indirect functional correlations. We used the String database to search for proteins related to HERPUD1.

### GeneMANIA analysis

GeneMANIA (http://www.genemania.org) [35] is a website for real-time prediction of gene function, and is also used to construct protein-protein interaction (PPI) networks, protein-DNA interactions, signaling pathways, protein domains, phenotypic screening, and other processes. We used GeneMANIA to analyze the proteins that interact with HERPUD1.

### cBioPortal analysis

The cBioPortal database (https://www.cbioportal.org/) [36] integrates TCGA, ICGC, GEO, and other databases to simplify the cancer tissues and cell lines to molecular expression profile data, to obtain the easily understood genetic, epigenetic, gene expression, and proteomics data. We used the cBioPortal database to analyze HERPUD1 gene mutations.

### Statistical analysis

SPSS Statistics ver.22.0 software (IBM, Armonk, NY, USA) was used for statistical analysis. Chi-squared test and Fisher exact probability test were used for the count data, one-way analysis of variance for the measurement data, and Spearman’s rank correlation analysis for the analysis of correlation. *P* < 0.05 was considered as statistically significant.

## Results

### The expression of HERPUD1 in different ovarian tissues and its relationship with clinicopathological parameters

**Fig. 1:**
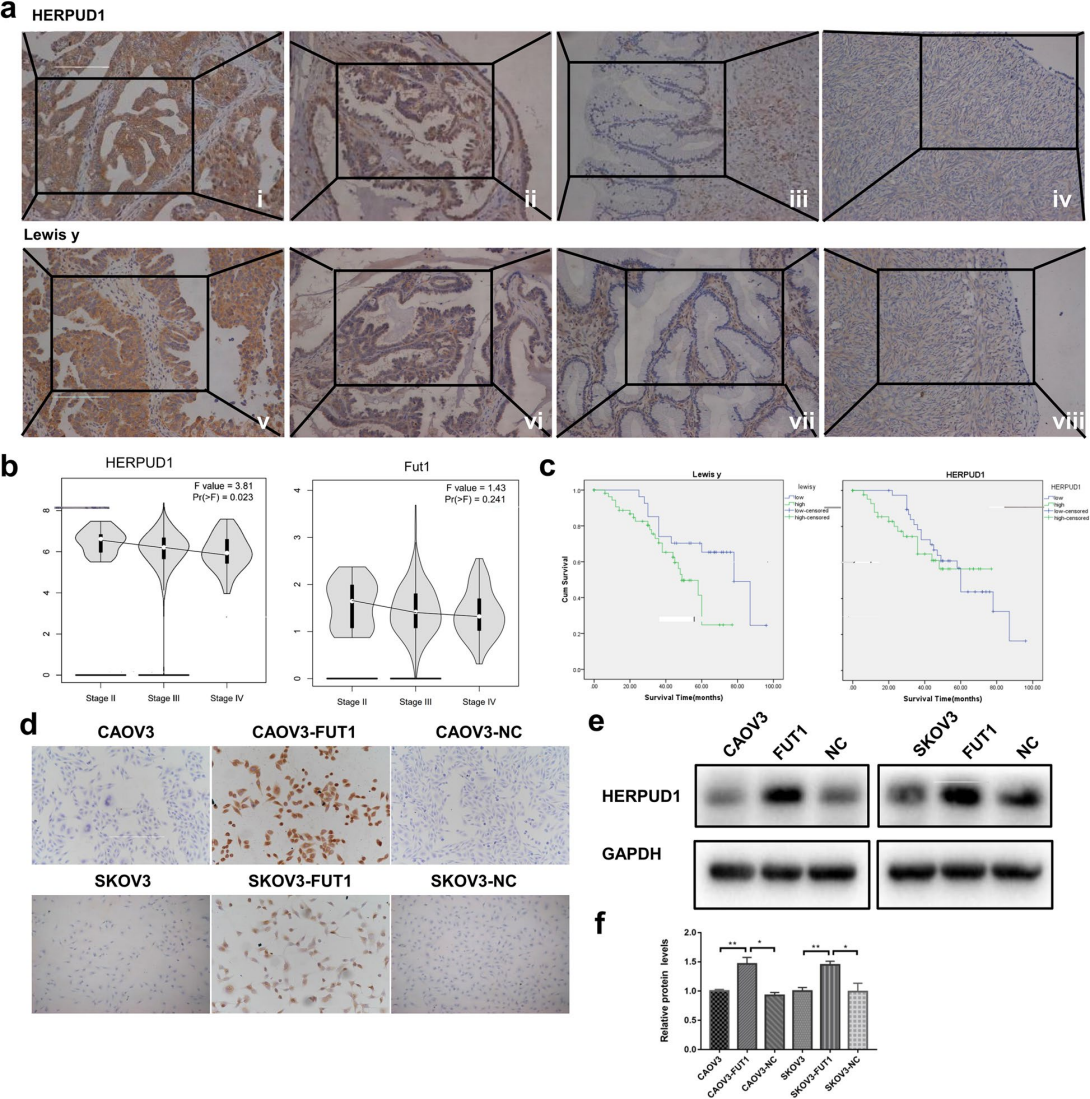
The expression of HERPUD1 and Lewis y in different ovarian tissues, and the expression of HERPUD1 is regulated by Lewis y. **a** The expression of HERPUD1 and Lewis y in different ovarian tissues (× 200).i.v: epithelial ovarian cancer; ii, vi: epithelial borderline ovarian tumor; iii, vii: benign epithelial ovarian tumor; iv, viii: normal ovary. **b** The relative expression of HERPUD1 and FUT1 in ovarian cancer patients of different FIGO stages in GEPIA database. **c** Analysis of the relationship between the expression of HERPUD1, Lewis y and the prognosis of ovarian cancer. **d** After transfection with α1,2-FUT1 plasmid, the expression of Lewis y increased was detected by immunocytochemistry. **e**, **f** Western blotting showed that after overexpressing Lewis y, HERPUD1 protein expression increased. Due to the different transfer conditions, the antibody incubation was performed after the membrane was cut. Data are presented as the mean ± SEM (*n* = 3 per group), *P < 0.05, **P < 0.01,*** *P* < 0.001 vs. the NC group

In immunohistochemistry, we used ex vivo tissue samples from clinical patients to detect the relationship between HERPUD1 expression and clinicopathological parameters. HERPUD1 was mainly expressed in the cell membrane and cytoplasm. The positive expression rate and high expression rate of HERPUD1 in the malignant group were significantly higher than that in the benign group and normal group (P all < 0.001) (Fig. 1a, Table 1). This study included 86 cases of malignant ovarian cancer. The high expression of HERPUD1 decreased with advance stages (*P* = 0.016), which is consistent with GEPIA website results (Fig. 1b), indicating that HERPUD1 may be more effective in the early stages of the tumor. Among the pathological types, the high expression rate of HERPUD1 in endometrioid group was significantly higher than that of the serous group, clear cell carcinoma group and poorly differentiated adenocarcinoma group (*P* = 0.001, 0.011, 0.023) (Table 2).

**Table 1:** The expression of HERPUD1 and Lewis y in different ovarian tissues

Groups	Cases	HERPUD1							Lewis y					
		(-)	(+)	(++)	(+++)	Positive rate%	High expression rate%		(-)	(+)	(++)	(+++)	Positive rate%	High expression rate%
Normal	12	8	4	0	0	33.3	0		10	2	0	0	16.7	0
Benign	9	5	3	1	0	44.4	11.1		5	2	1	1	44.4	22.2
Borderline	12	3	5	2	2	75.0	33.3		3	3	4	2	75.0*	50.0*
Malignant	86	15	24	27	20	82.6***	54.7***		11	17	34	24	87.2***	67.4***
Notes: *p<0.05,***p<0.001

**Table 2:** Association between HERPUD1 and Lewis y expression and pathological features in ovarian cancer

Features	Cases	HERPUD1			Lewis y		
		High expression Cases	High expression rate%	*P*-value	High expression Cases	High expression rate%	*P*-value
**FIGO stage**				0.016*			0.01*
I-II	34	24	70.6		16	47.1	
III-IV	52	23	44.2		42	80.8	
**Differentiation**				*0.123*			0.012*
Well-Moderate	30	13	43.3		15	50.0	
Poorly	56	34	60.7		43	76.8	
**LN metastasis**				*0.604*			0.006**
No	46	24	52.2		24	52.2	
Yes	22	10	45.5		19	86.4	
no lymphadenectomy	18	13	72.2		15	83.3	
**Pathologic type**							
Serous	47	20	42.6	0.001***	25	53.2	
Mucinous	4	3	75.0		3	75.0	
Clear cell carcinoma	7	3	42.9	0.011*	5	71.4	
Endometrioid	11	11	100.0		11	100.0	0.004**
Poorly differentiated adenocarcinoma	17	10	58.8	0.023*	14	82.4	0.044*

*Abbreviations: FIGO* International Federation of Gynecology and Obstetrics, *LN* Lymph nodes

*p < 0.05, **p < 0.01,***p < 0.001

### The correlation between HERPUD1 and Lewis y expression in ovarian tissues

Similar to HERPUD1, Lewis y was also mainly expressed in cell membrane and cytoplasm. The positive expression rate and high expression of Lewis y in ovarian cancer were significantly higher than that in benign tumors and normal ovarian epithelial tissue (P all < 0.001). In addition, the positive expression rate and high expression rate of Lewis y in borderline ovarian tumors were higher than that of benign tumors and normal ovarian epithelial tissue (*P* = 0.012, 0.014) (Fig. 1a, Table 1). The high expression rate of Lewis y was positively correlated with FIGO stage, differentiation, and lymph node metastasis (P = 0.01, P = 0.012, *P* = 0.006). Among the pathological types, the high expression rate of Lewis y in the serous group was significantly lower than that of the endometrioid group and poorly differentiated adenocarcinoma group (*P* = 0.004, 0.044) (Table 2).

In 119 cases of ovarian tissue samples, 52 cases showed high expression of HERPUD1 and 67 cases showed low expression; 66 cases had high expression of Lewis y, and 53 cases had low expression (Table 3). Further analysis of the correlation between HERPUD1 and Lewis y expression in ovarian tissue showed that there was a significant positive correlation between the expression of the two proteins (Spearman correlation coefficient was 0.319, *P* < 0.001). In 34 cases of Early stage ovarian cancer tissue samples, 24 cases showed high expression of HERPUD1 and 10 cases showed low expression; 16 cases had high expression of Lewis y, and 18 cases had low expression (Table 3). Further analysis of the correlation between HERPUD1 and Lewis y expression in ovarian tissue showed that there was a significant positive correlation between the expression of the two proteins (Spearman correlation coefficient was 0.776, *P* < 0.001).

**Table 3:** Relevance of HERPUD1 and Lewis y expression in ovarian tissues

Lewis y	HERPUD1		Total	Lewis y(FIGOI-II)	HERPUD1(FIGOI-II)		Total
	low expression	high expression			low expression	high expression	
**low expression**	41	12	53	**low expression**	10	8	18
**high expression**	26	40	66	**high expression**	0	16	16
**Total**	67	52	119	**Total**	10	24	34

### Relationship between the expression of HERPUD1/Lewis y and the patient survival

Follow-up of patients with ovarian cancer (as of January 30, 2021) by univariate Kaplan-Meier analysis showed that the average survival time of the lewis y high expression group was 49.57 months, whereas the average survival time of the low expression group was 70.37 months. Thus, high expression of lewis y was associated with shortened overall survival (*P* = 0.031) (Fig. 1c). The average survival time of the HERPUD1 high expression group was 54.94 months, whereas the average survival time of the HERPUD1 low expression group was 62.98 months. However, the expression of HERPUD1 was not statistically significant with survival. In addition, the FIGO stage and lymph node metastasis were both related to poor prognosis (*P* < 0.05) (Table 4; Fig. 1c). According to analysis of the relationship between different clinicopathological parameters and the prognosis of ovarian cancer patients by the Cox regression model, the FIGO stage, lymph node metastasis, and lewis y expression affected the survival time of ovarian cancer patients (*P* < 0.05). Moreover, multivariate Cox regression analysis revealed that the FIGO stage, lymph node metastasis were independent risk factors affecting the prognosis of patients with ovarian cancer (*P* < 0.05) (Table 5).

**Table 4:** Univariate Kaplan-Meier prognostic analysis of ovarian cancer

Variable	Characteristics	(Log-rank) *P*-value
**Age at diagnosis**	<50 years vs ≥ 50 years	***0.639***
**FIGO stage**	I-II vs III-IV	0.003**
**Differentiation grade**	Well-moderate vs. poor	***0.398***
**LN metastasis**	Negative vs positive	0.001***
**HERPUD1**	Low vs high	***0.694***
**Lewis y**	Low vs high	0.031*
Notes: *p<0.05, **p<0.01, ***p<0.001

**Table 5:** Univariate and multivariate Cox regression analysis of patients with ovarian cancer

Variables	Univariate analysis		Multivariate analysis	
	*P*-value	Hazard ratio (95% CI)	*P*-value	Hazard ratio (95% CI)
**Age at diagnosis**	***0.643***	0.853 (0.435–1.672)	***0.523***	0.724 (0.353–1.488)
**FIGO stage**	0.006**	3.265 (1.4.7–7.577)	0.004**	3.505 (1.496–8.214)
**Differentiation grade**	***0.197***	1.496 (0.811–2759)	***0.526***	1.168 (0.625–2.183)
**LN metastasis**	0.001***	1.956(1.307–2.926)	0.001***	2.148 (1.377–3.351)
**HERPUD1**	***0.697***	1.147 (0.575–2.286)	***0.176***	1.739 (0.805–3.756)
**Lewis y**	0.038*	2.308 (1.049–5.078)	***0.872***	1.019 (0.421–2.467)

*Abbreviations: FIGO* International Federation of Gynecology and Obstetrics, *LN* Lymph node

Notes: *p<0.05, **p<0.01, ***p<0.001

### Lewis y regulates the expression of HERPUD1 in ovarian cancer cells

After overexpression of Lewis y in ovarian cancer cells, and the expression of HERPUD1 protein also increased significantly (*P* < 0.05) (Fig. 1d-f), indicating that the expression of HERPUD1 is regulated by Lewis y. As Lewis y is expressed in whole protein, immunohistochemistry was used to detect its overall expression in cells.

### Enrichment analysis of HERPUD1 functional networks in ovarian serous cystadenocarcinoma

**Fig. 2:**
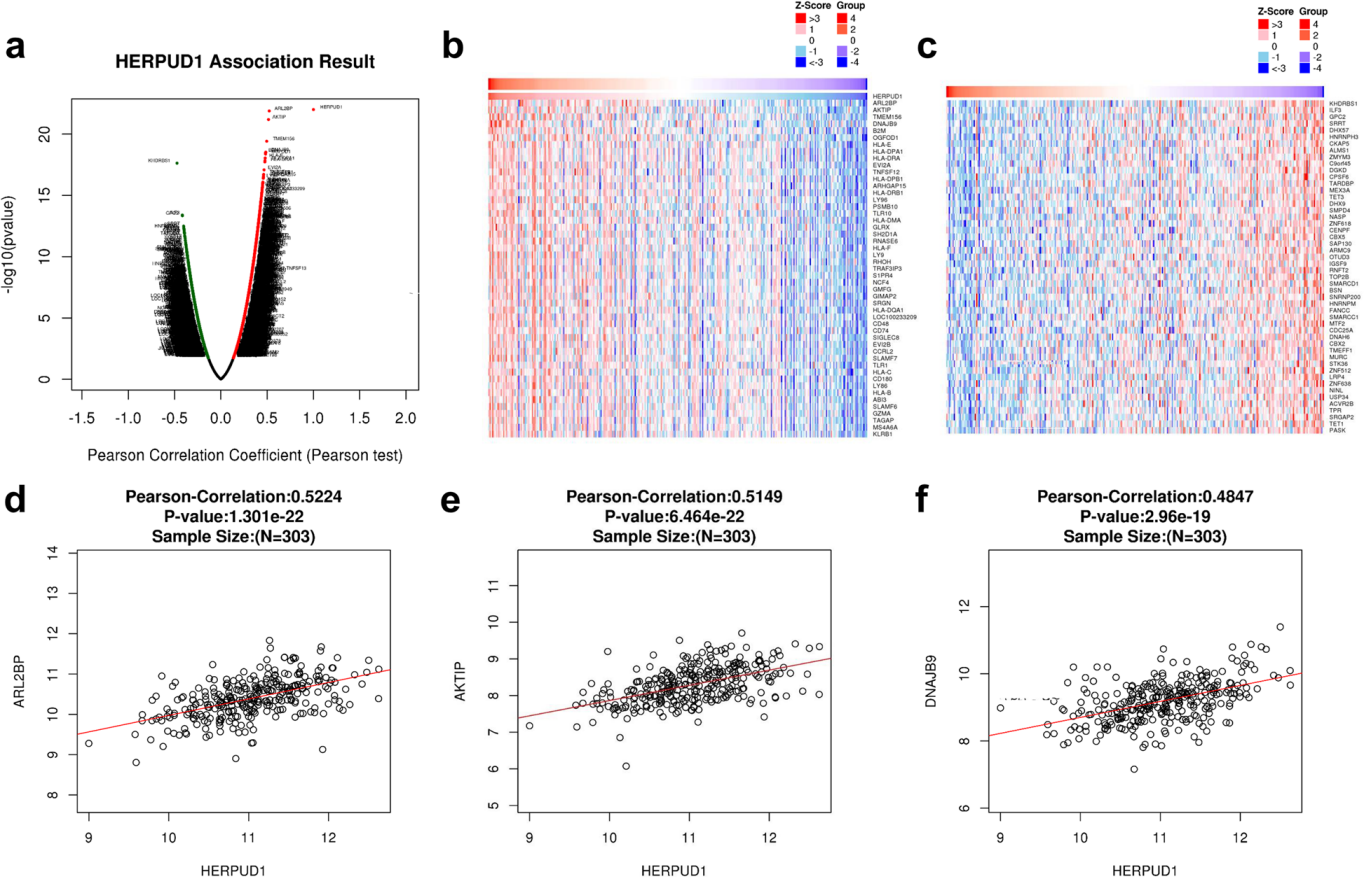
Differentially expressed genes in correlation with HERPUD1 in ovarian cancer. **a** Correlations between HERPUD1 and genes differentially expressed in ovarian cancer. **b**, **c** Top 50 genes positively (red) and negatively (green) correlated with HERPUD1 in ovarian cancer shown as heat maps. **d**–**f** Scatter plot of the Pearson correlation of HERPUD1 expression compared with expression of ARL2BP (**d**), AKTIP (**e**) and DNAJB9 (**f**)

We used the Function module in LinkedOmics to analyze the mRNA sequencing data of 581 patients with Ovarian Serous Cystadenocarcinoma in the TCGA database. As shown in the volcano diagram (Fig. 2a), there are 1695/1681 genes that are significantly positively/negatively related to HERPUD1 (dark red/green dots)(false discovery rate [FDR] < 0.01). The heat map shows the first 50 gene sets that are significantly positively/negatively correlated with HERPUD1 (Fig. 2b, c). This result indicates that HERPUD1 has a wide range of effects in regulating immunity, cytokine activity and information exchange between cells. The statistical scatter plot of a single gene shows that the expression of HERPUD1 is significantly positively correlated with ARL2BP, AKTIP and DNAJB9 (Fig. 2d-f), which play an important role in regulating the integrity of microtubules, maintaining the cell cycle, maintaining telomere homeostasis, and protecting cells from apoptosis caused by ERS.

**Fig. 3:**
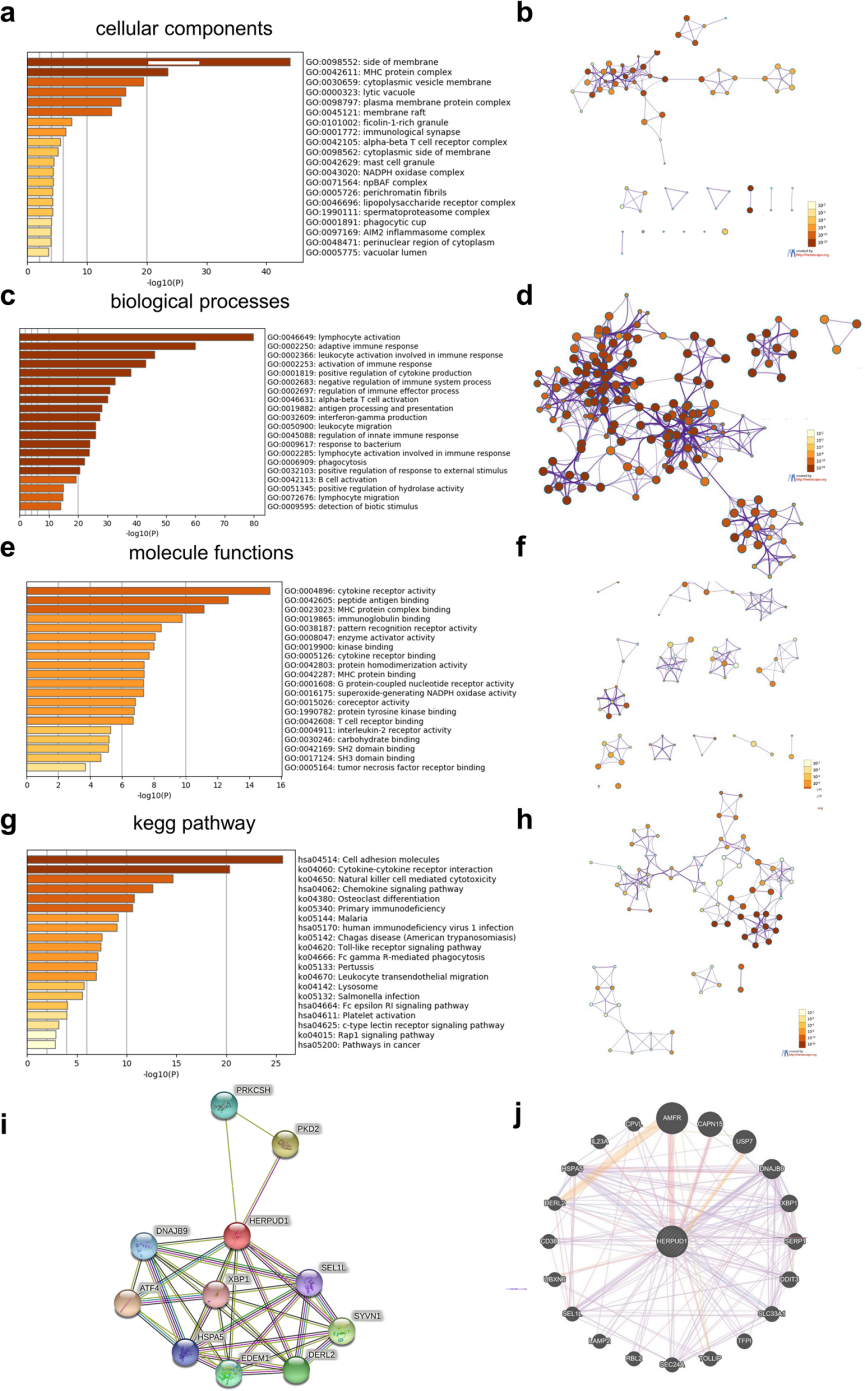
Significantly enriched GO annotations and KEGG pathways of HERPUD1 related genes in ovarian cancer. **a**, **b** Top 20 cellular components enriched in relation to HERPUD1, shown with bar graph and network. **c**, **d** Top 20 biological processes enriched in relation to HERPUD1, shown with bar graph and network. **e**, **f** Top 20 molecular functions enriched in relation to HERPUD1, with bar graph and network. **g**, **h** KEGG enriched terms shown with bar graph and network. **i** PPI network analysis using STRING database. **j** PPI network analysis using GeneMANIA database. The above results were colored by *P*-value, where terms containing more genes tend to have a more significant *P*-value

GO and KEGG enrichment analysis were performed on the functions of HERPUD1 and its related differential genes. The GO results show that HERPUD1 and its related differential genes are mainly located in side of membrane, MHC protein complex, cytoplasmic vesicle membrane and other structures (Fig. 3a, b and Supplementary Table 3). The GO results indicate HERPUD1 is mainly involved in activating the immune response, positive regulation of cytokine production, regulating immune effects, and other biological processes, such as lymphocyte activation, adaptive immune response, leukocyte activation involved in immune response, etc. (Fig. 3c, d and Supplementary Table 4). The molecular functions of HERPUD1 and its related genes mainly include regulating the activities of cytokine receptor, pattern recognition receptor, etc., and it can interact with MHC protein complex, immunoglobulin, cytokine receptor, etc. (Fig. 3e, f and Supplementary Table 5). KEGG enrichment analysis results showed that the signaling pathways interacting with HERPUD1 and its related differential genes include Cell adhesion molecules, Natural killer cell mediated cytotoxicity, Toll-like receptor signaling pathway, etc. (Fig. 3g, h and Supplementary Table 6). The above-mentioned signaling pathways can participate in the occurrence and development of a variety of tumors.

### HERPUD1 network of kinase, miRNA, or transcription factor targets in ovarian cancer

To further explore the target of HERPUD1 in ovarian cancer, we analyzed the kinase, miRNA, and transcription factor target networks of the positively related gene set generated by GSEA. The top five most important target networks are mainly related to tumor occurrence, activated immune receptors and downstream signal transduction coupling, cancer cell division, and cell cycle checkpoint activation during DNA stress (Table 6 and Supplementary Tables 7, 8 and 9). The miRNA target network identified is related to multiple sequences. The transcription factor target network is mainly related to the regulation of transcription extension, chromosome structure formation, natural immunity and adaptive immunity, cell proliferation, differentiation, apoptosis, and other physiological processes.

**Table 6:** The kinase, miRNA and transcription factor-target networks of HERPUD1 in ovarian cancer

Enriched Category	Geneset	LeadingEdgeNum	FDR
Kinase Target	LCK proto-oncogene, Src family tyrosine kinase	20	0
	spleen associated tyrosine kinase	15	0
	LYN proto-oncogene, Src family tyrosine kinase	20	0
	aurora kinase B	29	0
	ATR serine/threonine kinase	43	0
miRNA Target	ACAGGGT,MIR-10A,MIR-10B	40	0
	TCCCCAC,MIR-491	16	0
	CCAGGTT,MIR-490	27	0
	CAGCAGG,MIR-370	38	0
	TGGTGCT,MIR-29A,MIR-29B,MIR-29C	119	0
Transcription Factor Target	V$ELF1_Q6	71	0
	V$IRF_Q6	63	0
	STTTCRNTTT_V$IRF_Q6	67	0
	V$PU1_Q6	59	0
	RYTTCCTG_V$ETS2_B	297	0

### PPI analysis using STRING and GeneMANIA databases

Through String and GeneMANIA to perform PPI enrichment analysis (Fig. 3i, j), the results showed that proteins that interact with HERPUD1 are mainly enriched in the unfolded protein response (UPR) which is involved in regulating endoplasmic reticulum stress, ubiquitin-dependent degradation, assembly of multimeric protein complexes within the endoplasmic reticulum, protein transport, ERS-induced apoptosis, tumor metastasis, innate and adaptive immunity, and other processes.

### Genomic changes of HERPUD1 in ovarian cancer

**Fig. 4:**
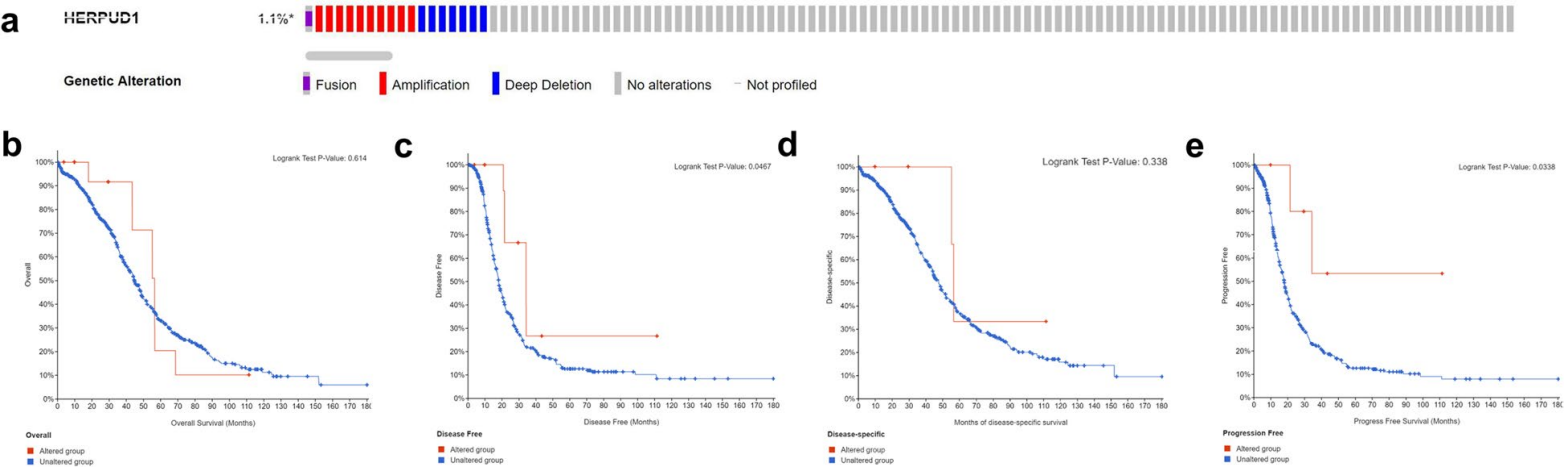
HERPUD1 gene mutation and its effect on the survival and prognosis of patients with ovarian cancer. **a** Types of HERPUD1 gene mutations in the cBioPortal database. **b**-**e** The effect of HERPUD1 gene mutation on OS (**b**), DFS (**c**), DSS (**d**), PFS (**e**) in patients with ovarian cancer. Abbreviations: OS, overall survival; DFS, disease free survival; DDS, disease specific survival; PFS, progression free survival

We use cBioPortal to determine the type and frequency of HERPUD1 gene mutations in ovarian cancer, based on the sequencing data of ovarian cancer patients in the TCGA database. 18 of the 1668 (1.1%) ovarian cancer patients had mutations in the HERPUD1 gene (Fig. 4a), and the genetic changes included fusion, amplification and deep deletion. HERPUD1 gene mutation had no significant effect on the overall survival(OS) (Fig. 4b), disease free survival(DFS) (Fig. 4c), and disease specific survival(DSS) (Fig. 4d) of patients with ovarian cancer (*P* > 0.05), but patients with HERPUD1 mutations had longer progression-free survival times(PFS) (Fig. 4e) (*P* = 0.0338), indicating that mutations in the HERPUD1 gene may be involved in affecting the prognosis of patients.

### HERPUD1 expression and transfection in various ovarian cancer cells

**Fig. 5:**
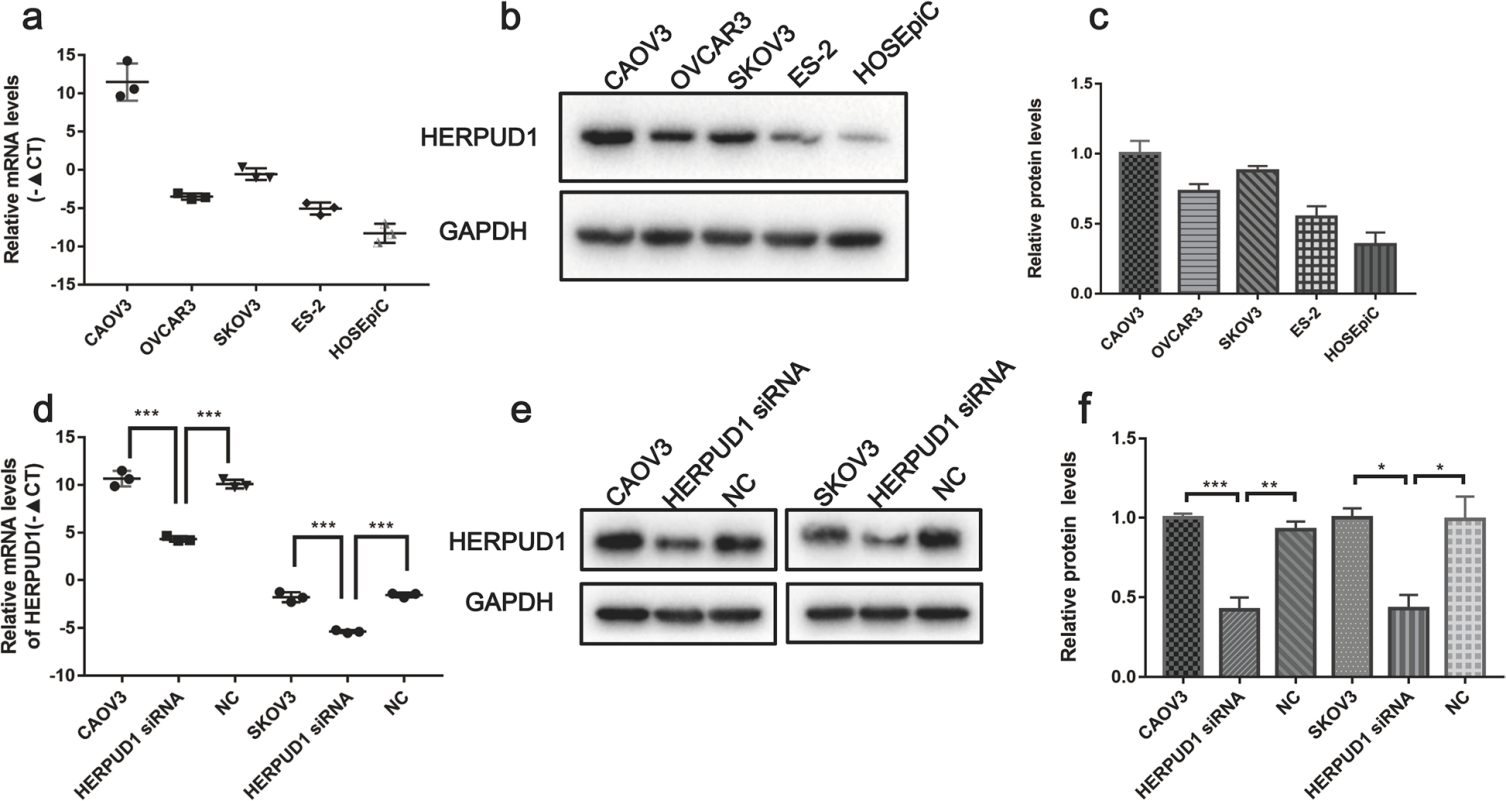
Detect the expression of HERPUD1 in different ovarian cell lines and the degree of inhibition of HERPUD1 expression after transfection with siRNA. **a**-**c** HERPUD1 mRNA and protein expression in ovarian cancer and normal cell lines. **d**-**f** Knock down HERPUD1 in ovarian cancer cell lines CAOV3 and SKOV3 to detect the expression of HERPUD1 mRNA and protein. Due to the different transfer conditions, the antibody incubation was performed after the membrane was cut. Data are presented as the mean ± SEM (*n* = 3 per group), *P < 0.05, **P < 0.01,*** *P* < 0.001 vs. the NC group

We found that HERPUD1 expression in ovarian cancer cell lines(CAOV3, OVCAR3, SKOV3, ES-2) was higher than that in normal ovarian epithelial cells(H0SEpiC), and was highest in CAOV3 and SKOV3 cell lines (Fig. 5a-c). Therefore, we selected these two high-expressing cell lines for further experiments, using siRNA for knockdown, and analyzed the knockdown efficiency by western blot and qRT-PCR experiments (*P* < 0.05) (Fig. 5d-f).

### Knockdown of HERPUD1 affects the ovarian cancer cell proliferation, apoptosis, and cell cycle

**Fig. 6:**
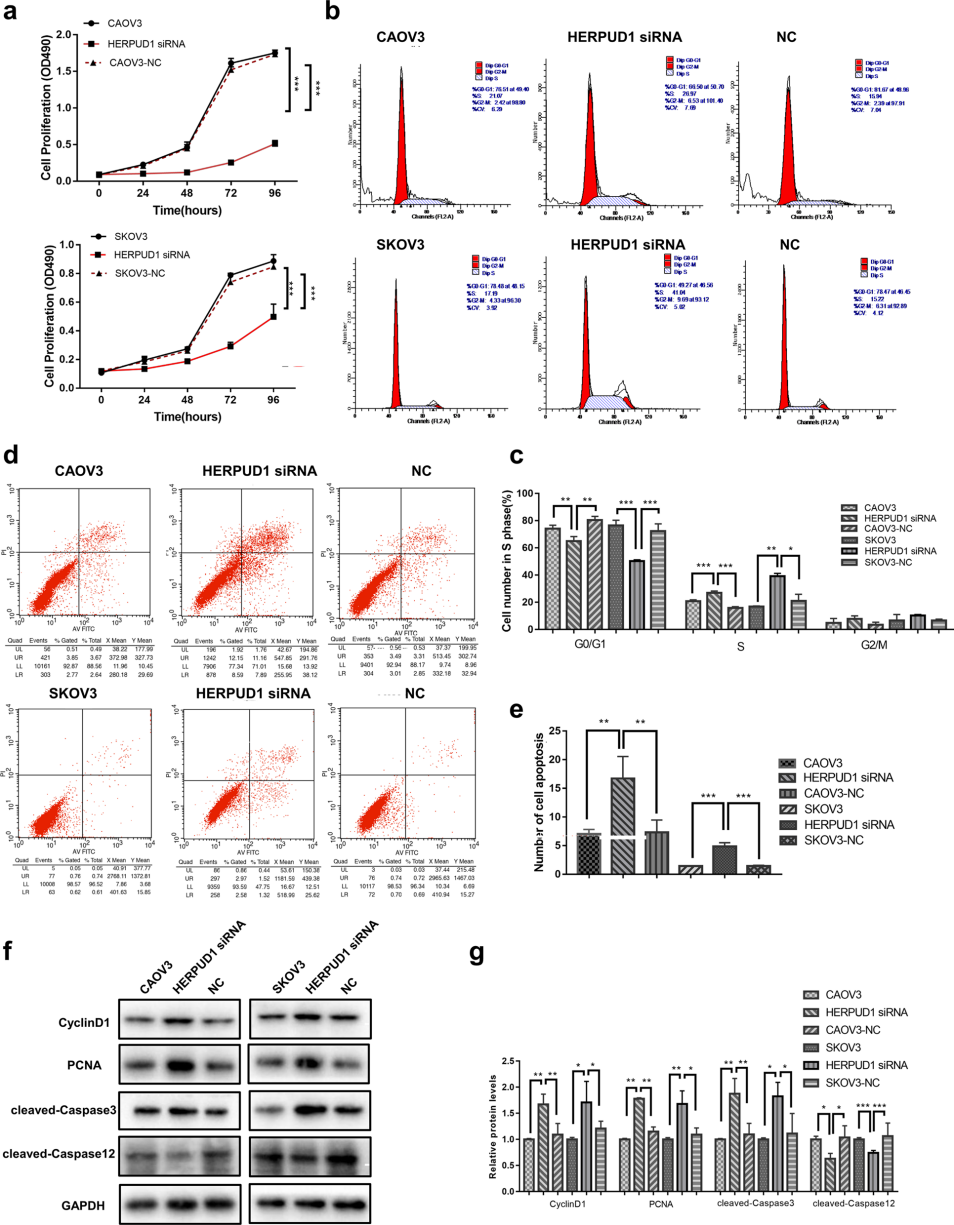
The effect of knockdown of HERPUD1 on proliferation, apoptosis, and cell cycle in CAOV3 and SKOV3 ovarian cancer cells. **a** The effect of knockdown of HERPUD1 on the proliferation in CAOV3 and SKOV3 cell lines (MTT assay). **b**, **c** Flow cytometry to detect the effect of knockdown of HERPUD1 on the cell cycle. **d**, **e** Flow cytometry to detect the effect of knockdown of HERPUD1 on cell apoptosis. **f**, **g** Knockdown of HERPUD1 affects the expression of apoptosis-related proteins Caspase3, Caspase12, and cell cycle-related proteins Cyclin D1. Due to the different transfer conditions, the antibody incubation was performed after the membrane was cut. Data are presented as the mean ± SEM (*n* = 3 per group), *P < 0.05, **P < 0.01,*** *P* < 0.001 vs. the NC group

Through the MTT test, we found that knocking down HERPUD1 significantly reduced the proliferation ability of ovarian cancer cells (*P* < 0.05) (Fig. 6a). We further tested the cell cycle using flow cytometry and found that compared with the control group, the ratio of cells in S phase and G2/M phase increased after knocking down HERPUD1 (*P* < 0.05) (Fig. 6b, c). Western blotting showed that the expression of cycle-related proteins CyclinD1, and PCNA increased (*P* < 0.05) (Fig. 6f, g), indicating that knockdown of HERPUD1 induces ovarian cancer cells to enter S phase from G0/G1 by upregulating the expression of these proteins and causing S phase block, which affects cell cycle progression. The effect of HERPUD1 on cell apoptosis was also analyzed by flow cytometry, and we found that knocking down HERPUD1 significantly promoted cell apoptosis (*P* < 0.05) (Fig. 6d, e). Western blotting showed that the expression of cleaved-Caspase12, which is related to ERS and apoptosis, was slightly downregulated, while the expression of the most important terminal cleaved enzyme cleaved-Caspase3 was upregulated (*P* < 0.05) (Fig. 6f, g). These results showed that knocking down HERPUD1 ultimately affects cell apoptosis, likely by activating apoptosis pathways other than Caspase12.

### Knockdown of HERPUD1 affects autophagy in ovarian cancer cells

**Fig. 7:**
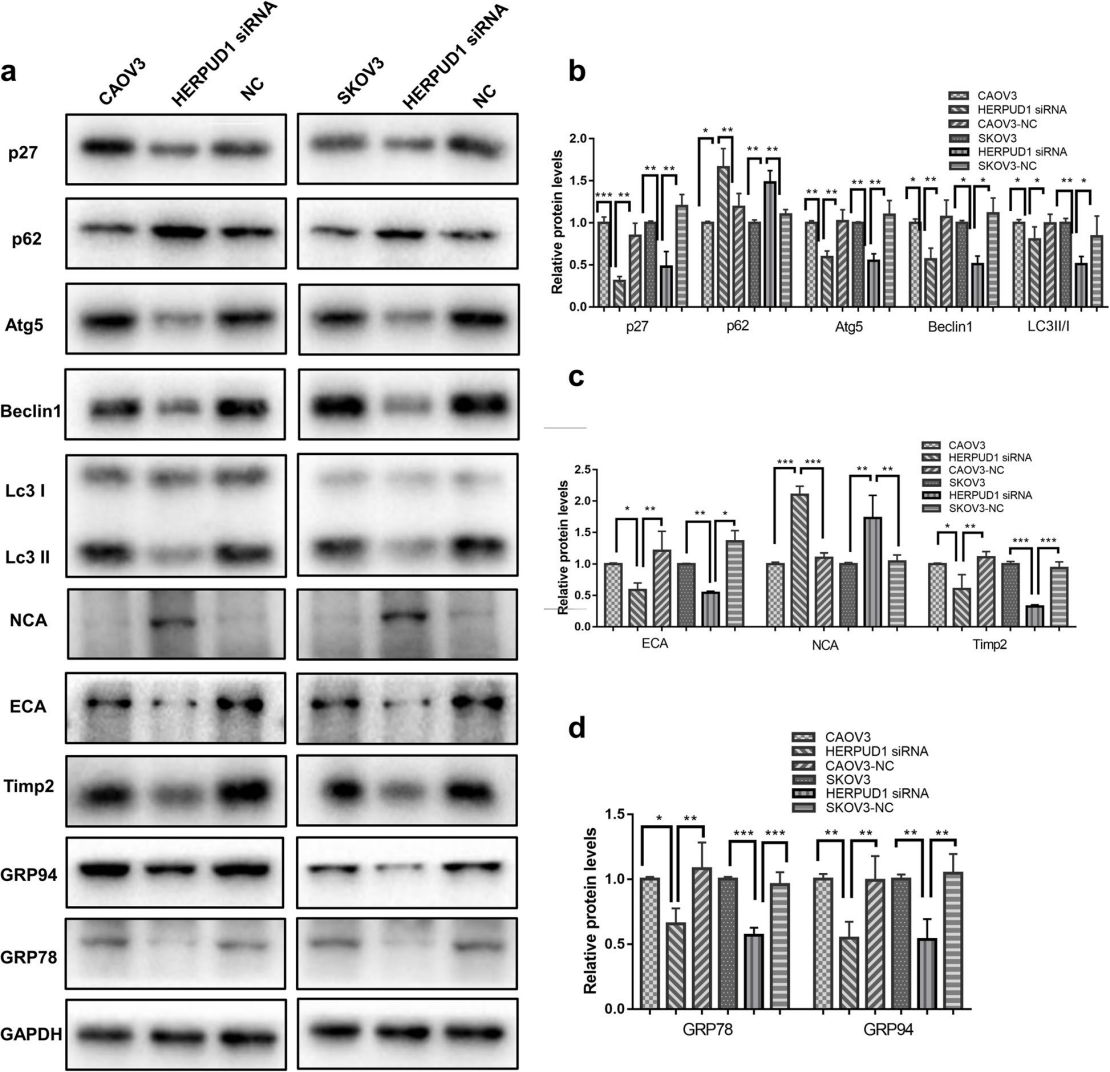
HERPUD1 regulates the expression of autophagy-related proteins, EMT-related proteins and ERS-related proteins in CAOV3 and SKOV3 ovarian cancer cell lines. **a**, **b** In CAOV3 and SKOV3 ovarian cancer cell lines, knockdown of HERPUD1 reduced the expression of protein p27, Atg5, Beclin1, and reduced the LC3 II/I ratio, but increased the expression of protein p62. **a**, **c** In CAOV3 and SKOV3 ovarian cancer cell lines, knockdown of HERPUD1 reduced the expression of E-cadherin and Timp2, but increased the expression of N-cadherin. **a**, **d** In CAOV3 and SKOV3 ovarian cancer cell lines, knockdown of HERPUD1 reduced the expression of ERS marker protein GRP78 and GRP94. Due to the different transfer conditions, the antibody incubation was performed after the membrane was cut. Data are presented as the mean ± SEM (*n* = 3 per group), *P < 0.05, **P < 0.01,*** *P* < 0.001 vs. the NC group

After knocking down HERPUD1, the ratio of autophagy marker LC3II/I decreased significantly, whereas p62, which is negatively related to autophagy activity, increased significantly. This indicated that the quantity of autophagosomes was reduced and autophagy activity was weakened. In addition, the expression of Beclin1, which promotes the formation of autophagy membranes and guides the localization of autophagy-related proteins, was downregulated. The expression of Atg5, which is involved in the formation of autophagy bilayer membranes, and p27, which promotes the occurrence of autophagy, were also downregulated (*P* < 0.05) (Fig. 7a, b). This phenomenon indicates that autophagy was weakened after knocking down HERPUD1.

### Knockdown of HERPUD1 affects EMT in ovarian cancer cells

When we detected EMT-related proteins, we found that after knocking down HERPUD1, the expression of epithelial marker E-cadherin was downregulated, the expression of mesenchymal marker N-cadherin was upregulated, and the expression of Timp2, which inhibits the expression and activity of metalloproteinases, was downregulated (*P* < 0.05) (Fig. 7a, c). These data indicated that HERPUD1 inhibits the occurrence of epithelial-mesenchymal transition.

### Changes in the expression of endoplasmic reticulum stress-related proteins before and after HERPUD1 knockdown

After knocking down HERPUD1, we performed western blot experiments and found that the expression levels of GRP78 and GRP94, the key molecules of endoplasmic reticulum stress, were significantly reduced (*P* < 0.05) (Fig. 7a, d). This result indicated that the endoplasmic reticulum stress state was inhibited.

### Changes in the expression of PI3K/AKT/mTOR pathway and p38 MAPK pathway node proteins before and after knocking down HERPUD1

**Fig. 8:**
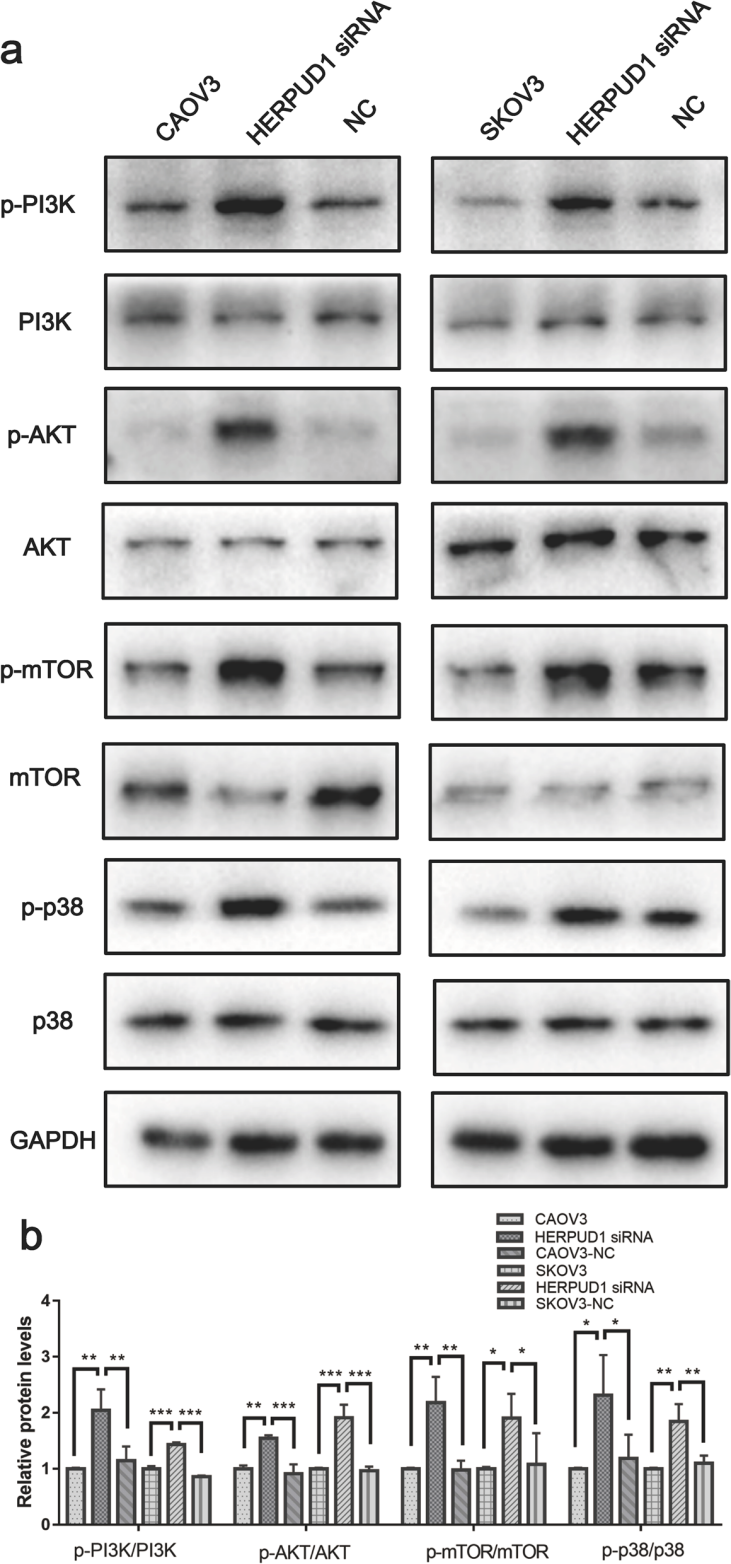
In CAOV3 and SKOV3 ovarian cancer cell lines, HERPUD1 inhibits PI3K/AKT and p38MAPK signaling pathways. **a**, **b** After knocking down HERPUD1, the phosphorylation ratio of PI3K / AKT / mTOR pathway-related nodal proteins (PI3K / AKT / mTOR) and p38MAPK pathway-related nodal protein (p38) increased. Due to the different transfer conditions, the antibody incubation was performed after the membrane was cut. Data are presented as the mean ± SEM (*n* = 3 per group), *P < 0.05, **P < 0.01,*** *P* < 0.001 vs. the NC group

Compared with the control group, HERPUD1 knockdown increased the ratio of p-PI3K/PI3K, p-AKT/AKT and p-mTOR/mTOR; the ratio of p-p38/p38 also increased (*P* < 0.05) (Fig. 8a, b). These results indicate that HERPUD1 inhibited the PI3K/AKT/mTOR signaling pathway and the p38 MAPK signaling pathway in ovarian cancer cell lines.

## Discussion

Ovarian cancer is the leading cause of death from gynecological tumors, therefore, exploring the molecular mechanisms underlying the occurrence and development of ovarian cancer is of pivotal for improving patient prognosis [37]. During tumorigenesis, adaptive stress promotes the unfolded protein response (UPR) and endoplasmic reticulum-related protein degradation (ERAD), and initiates and activates the survival cascade [38]. Studies have found that inhibiting the activity of important downstream proteins of ERAD can inhibit tumor growth [38]. The human HERPUD1 gene is located in the 16q13 locus and encodes a membrane protein with a ubiquitin-like (UBL) domain, which is believed to play a role in ER-associated degradation by recruiting ubiquitin [39–41]. Currently, HERPUD1 is rarely studied in tumors. Based on the results of IHC and GEPIA website, we first proposed that the expression level of HERPUD1 was widely upregulated in ovarian cancer tissue samples, especially in the early FIGO stages, indicating that ovarian cancer may require higher levels of ERAD to maintain tumor survival. In addition, the HERPUD1 gene mutation affects the prognosis of ovarian cancer, suggesting that HERPUD1 may be involved in the occurrence and development of ovarian cancer.

In order to better clarify the molecular mechanism, biological function, and related signaling pathways of HERPUD1 in ovarian cancer, we conducted enrichment analysis of HERPUD1 and related genes by PPI, GO, and KEGG pathway analysis, and found that these genes are mainly involved in immune regulation, the assembly and transport of protein complexes in the ER, regulation of ERS, ubiquitin-dependent degradation, ERS-induced apoptosis, tumor metastasis, and other biological processes. In the present study, knocking down HERPUD1 in cancer cell lines resulted in a significant decrease in the proliferation of these cells. We found that the cell cycle was blocked and apoptosis increased. We suspected that this might be the mechanism by which proliferation was inhibited. Chen et al. found that the knockdown of HERPUD1 in mouse ovarian granulosa cells also caused S phase arrest, consistent with our findings [42]. In the current study, HERPUD1 has a dual effect on the apoptosis of different cells. A previous study found that microRNA-384 mediated upregulation of HERPUD1 to promote endothelial cell apoptosis induced by angiotensin II [43]. Knockdown of HERPUD1 can protect ovarian granulosa cells from zearalenone (ZEA)-induced apoptosis [44]. However, in neurons and glioma cells, HERPUD1 promotes cell survival under endoplasmic reticulum stress conditions by inhibiting apoptosis [45, 46]. In the present study, cell apoptosis increased after knocking down HERPUD1, the expression of cleaved-Caspase3, the most important terminal cleavage enzyme in the process of apoptosis, was significantly increased, and Caspase12, which is one of the apoptotic pathways of ERS, decreased slightly. In the Caspase12 pathway, during ERS, IRE1α activates the recruited TRAF2 to cut and activate Caspase12, then activates Caspase9 and Caspase3 to cause caspase-mediated apoptosis [5, 47]. The results showed that the expression of HERPUD1 in ovarian cancer inhibited the occurrence of apoptosis and protected the survival of tumor cells, but not by activating Caspase12. The specific mechanism by which HERPUD1 is involved in apoptosis needs to be further studied.

As a protective mechanism for cells to adapt to the environment. When cancer cells lack energy, autophagy is activated and degrades its own substances to provide energy and promote cell survival. In contrast, autophagy can also lead to autophagic cell death [48–50]. Zhang et al. found that autophagy can enhance the chemotherapy resistance of cisplatin in ovarian cancer [51]. Ferraresi et al. found that enhancing autophagy can inhibit IL-6-induced cancer cell migration in ovarian cancer cells [52]. Therefore, the role of autophagy in the development and progression of ovarian cancer is bidirectional. In the present study, we found that after knocking down HERPUD1, autophagy is weakened. Interestingly, the inhibitory effect of HERPUD1 on autophagy was found in previous studies. Quiroga et al. found that knocking down HERPUD1 in HeLa and HEK293T cells can promote autophagy, and promote cell survival [53]. Studies have found that knocking down HERPUD1 enhances autophagy induced by ZEA [44]. We believe that the conflicting results may be related to cell types. It has been found that autophagy has different effects in different tumors [54]. Autophagy participates in different mechanisms and signal pathways; therefore, it has a bidirectional effect on the regulation of different tumors.

Current research shows that autophagy and EMT have a complex connection [55]. Autophagy inhibits the early stage of metastasis, and the activation of EMT is needed to break this process. EMT needs autophagy activation to maintain survival in metastatic spread [55]. Catalano et al. found that the induction of autophagy can lead to reduced migration and invasion in glioblastoma cells [56]. In the process of EMT, the silencing of the target gene Cadherin-6 of TGFβ can affect the EMT phenotype, activate of autophagy and inhibit the proliferation, migration and invasiveness of thyroid cancer cells [57]. The above research is consistent with the results of the present research. Under the ERS in early stages of tumors, HERPUD1 assists high levels of ERAD to promote autophagy, inhibit apoptosis to maintain cell survival, and inhibit EMT to inhibit metastasis in the early stages of tumors.

The regulation between autophagy and EMT is through multiple pathways: PI3K/AKT/mTOR, Beclin-1, p53 and JAK/STAT multiple signaling pathways, of which PI3K/AKT/mTOR is most critical [58]. Under adverse conditions such as cell hypoxia and nutrient deficiency, the PI3K/AKT/mTOR pathway is inhibited, which can induce the occurrence of autophagy, to maintain tumor cell survival [59, 60]. In addition, the activation of the PI3K/AKT signaling pathway can directly induce the occurrence of EMT by upregulating the expression of nuclear transcription factors such as Snail, Slug, Twist, and ZEB in the cell, thereby directly inhibiting the level of E-cadherin; or inducing the expression of matrix metalloproteinases to degrade E-cadherin [61, 62]. As the research found, WSP1 induces autophagy and inhibits EMT by downregulating the PI3K/AKT/mTOR pathway, thereby inhibiting colon cancer migration [63]. The p38MAPK signaling pathway is an important part of the MAPK cascade [64, 65]. p38MAPK can also significantly inhibit autophagy: on the one hand, p38 MAPK can phosphorylate Atg5, inhibit autophagic vesicle extension and the conversion of microtubule-associated protein LC3I to LC3II; on the other hand, it can downregulate ERK activity and significantly reduce the level of autophagy [66, 67]. In addition, studies have also found that the p38 MAPK pathway is involved in the EMT process. The pro-inflammatory factor TWEAK can enhance the EMT of human bronchial epithelial cells induced by TGF-β by activating the p38 MAPK/ZEB2 pathway, causing airway inflammation and remodeling, and promoting pulmonary fibrosis [68]. The present study found that HERPUD1 may inhibit PI3K/AKT/mTOR pathway and p38MAPK pathway in ovarian cancer cells, thereby inducing autophagy and preventing the process of EMT.

Lewis y antigen is a tumor-associated carbohydrate antigen. It is an oligosaccharide chain containing a double fucosyl group and acts as an ‘antenna’ on the cell surface to receive various signals from inside and outside the cell. In previous study, we found that the biological behaviors of cell proliferation, adhesion, metastasis, and drug resistance were also enhanced [19, 22, 24, 26, 69]. We previously identified DEGs after transfection of FUT1 gene in ovarian cancer CAOV3 cell line, and found that the endoplasmic ERS-related gene HSPA8 was significantly changed, indicating that Lewis y may be involved in the ERS process [28]. In the present study, we found that the expression of Lewis y and HERPUD1 was significantly correlated in the early stage of tumor, and proves that Lewis y regulates the expression of HERPUD1. ‘Maintain proliferative signaling’ is a hallmark of cancer, but it may play a more important role in early stage in ovarian cancer than in late stage, when “activation of invasion/metastasis” or “resistant cell death” occupy the overall clinical manifestation of ovarian cancer. Interestingly, the function of promoting cell survival of Lewis y we found earlier was highly consistent with HERPUD1. Lewis y significantly increased the proliferative capacity of ovarian cancer cells [70], which we subsequently found by activating PI3K/Akt signaling and promoting EGFR, TGF-β1, VEGF and b-FGF and other growth factors [19, 24, 26]. Lewis y antigen inhibits the apoptosis of ovarian cancer cells. Lewis y antigen confers cell adhesion-mediated drug resistance to the apoptosis in ovarian cancer cells by upregulation of Topo-I and Topo-II β [71]. Lewis(y) antigen induces the increasing expression of apoptosis-inhibiting proteins Bcl-2 and Bcl-xL, and decreases the expression of pro-apoptotic protein Bax, thus causing an increasing Bcl-2(Bcl-xL)/Bax ratio, which further inhibited the Activation of pro-caspase-3 [25]. Lewis y inhibits apoptosis and CAM-DR by activating the FAK signaling pathway and upregulating Bcl-2/Bcl-XL expression in ovarian cancer cell lines [72]. In addition, Lewis y also promotes autophagy in ovarian cancer cells. High expression of Lewis y antigen promote autophagy in the early stage, reduce the activity of the PI3K/Akt/mTOR pathway. With prolonged nutrient deprivation, the PI3K/Akt/mTOR/EIF4G2 pathway is activated, thereby inhibiting excessive autophagy and inducing cell death [18]. TGF-β1-mediated modification of Lewis y antigen can regulate autophagy and mitophagy by activating PI3K/Akt and Ras-Raf-MEK-ERK pathways in ovarian cancer cells, and Lewis y antigen modification can trigger mitochondrial membrane potential depolarization and enhance autophagy regulation [73]. In this study, we speculate that Lewis y may regulate HERPUD1 to affect the PI3K/Akt/mTOR signaling pathway in the early stage of the tumor, promote autophagy of tumor cells, inhibit apoptosis, and provide tumor cells a selective advantage under the metabolic stress environment, to protect cancer cells from various forms of cellular stress and promote the survival of tumor cells. Besides, Lewis y promotes the invasion and metastasis of ovarian cells, unlike HERPUD1, which related to its regulation of the expression of various molecules other than HERPUD1 including CD147, integrin α5β1 and CD44 [20, 23, 74].

As a member of the degradation complex, HERPUD1 can precisely regulate the degradation process, regulate the death pathway of endoplasmic reticulum stress to maintain cell survival. Therefore, HERPUD1 is expected to become a new target for ovarian cancer chemotherapy. This research still has some shortcomings. The role and mechanism of HERPUD1 in ovarian cancer needs to be further studied in vivo in the future. In addition, the samples involved in this study were obtained retrospectively, and the clinical application of HERPUD1 needs further comprehensive and in-depth analysis.

## Conclusions

In summary, in the early stage of ovarian cancer, HERPUD1 is significantly overexpressed, and is regulated by Lewis y antigen, can promote the occurrence of autophagy and inhibit the apoptosis and EMT process by inhibiting the PI3K/AKT/mTOR and p38MAPK pathways, which provides favorable conditions for tumor cell survival under ERS in the early stage of ovarian cancer.

## Supplementary Information

**Additional file 1.** Supplementary Figures.

**Additional file 2.** Supplementary Tables.

## Data Availability

All data generated or analysed during this study are included in this published article and its supplementary information files.

## Abbreviations

ER: Endoplasmic reticulum
HERPUD1: Homocysteine inducible endoplasmic reticulum protein with ubiquitin-like domain 1
GO: Gene Ontology
KEGG: Kyoto Encyclopedia of Genes and Genomes
EMT: Epithelial-to-mesenchymal
FIGO: International Federation of Gynecology and Obstetrics
EOC: Epithelial ovarian carcinoma
ERS: Endoplasmic reticulum stress
UPR: Unfolded protein response
ERAD: ER-associated degradation
PBS: Phosphate-buffered saline
LN: Lymph node
SDS-PAGE: Sodium dodecyl sulfate-polyacrylamide gel electrophoresis
ZEA: Zearalenone
