# Supplementary material for: HERPUD1 promotes ovarian cancer cell survival by sustaining autophagy and inhibit apoptosis via PI3K/AKT/mTOR and p38 MAPK signaling pathways
Source: BMC Cancer. 2022 Dec 21;22:1338. doi: 10.1186/s12885-022-10248-5 (PMC9769045; doi:10.1186/s12885-022-10248-5)

Figure 1

Figure 1e SKOV3
HERPUD1

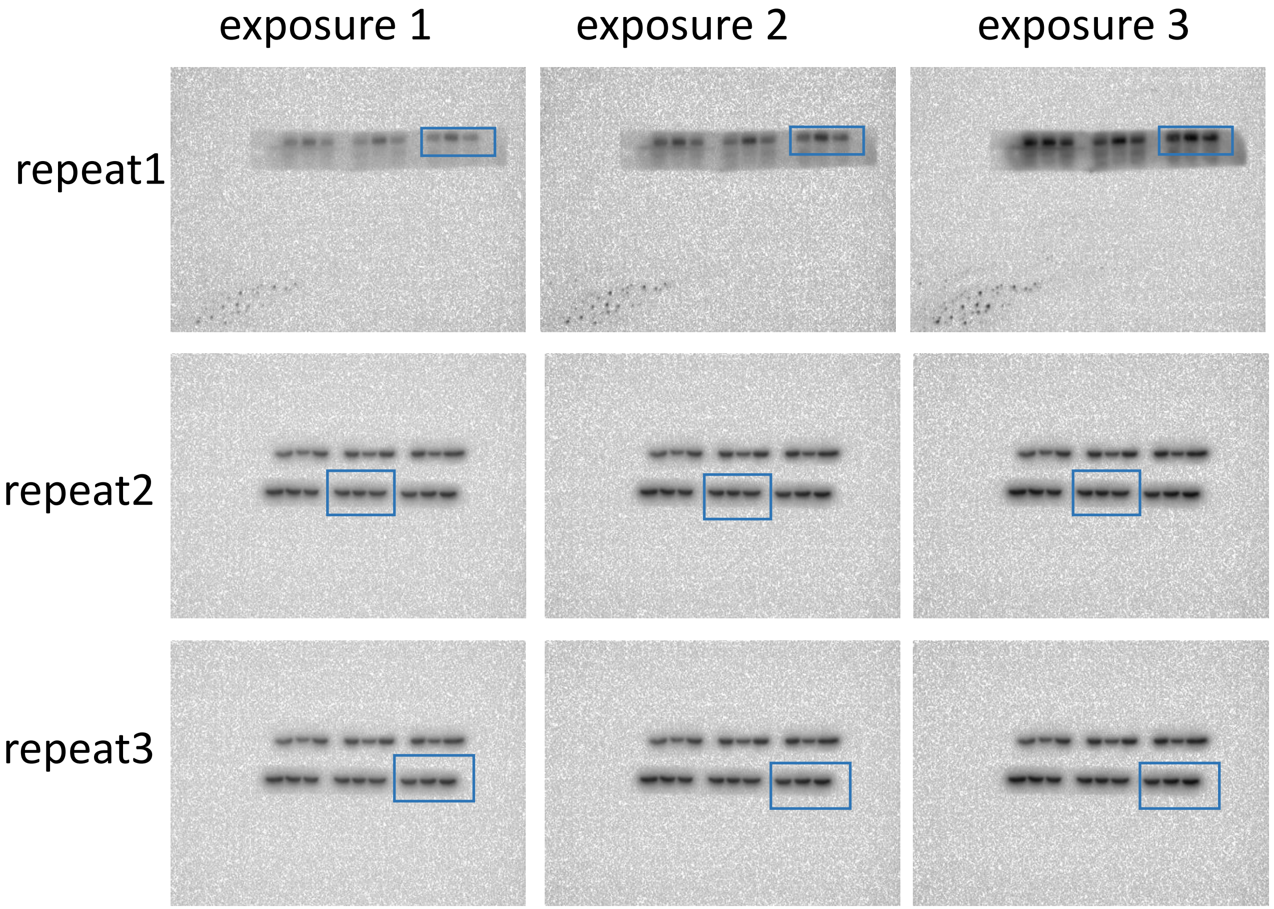

Figure 1

Figure 1e SKOV3
GAPDH

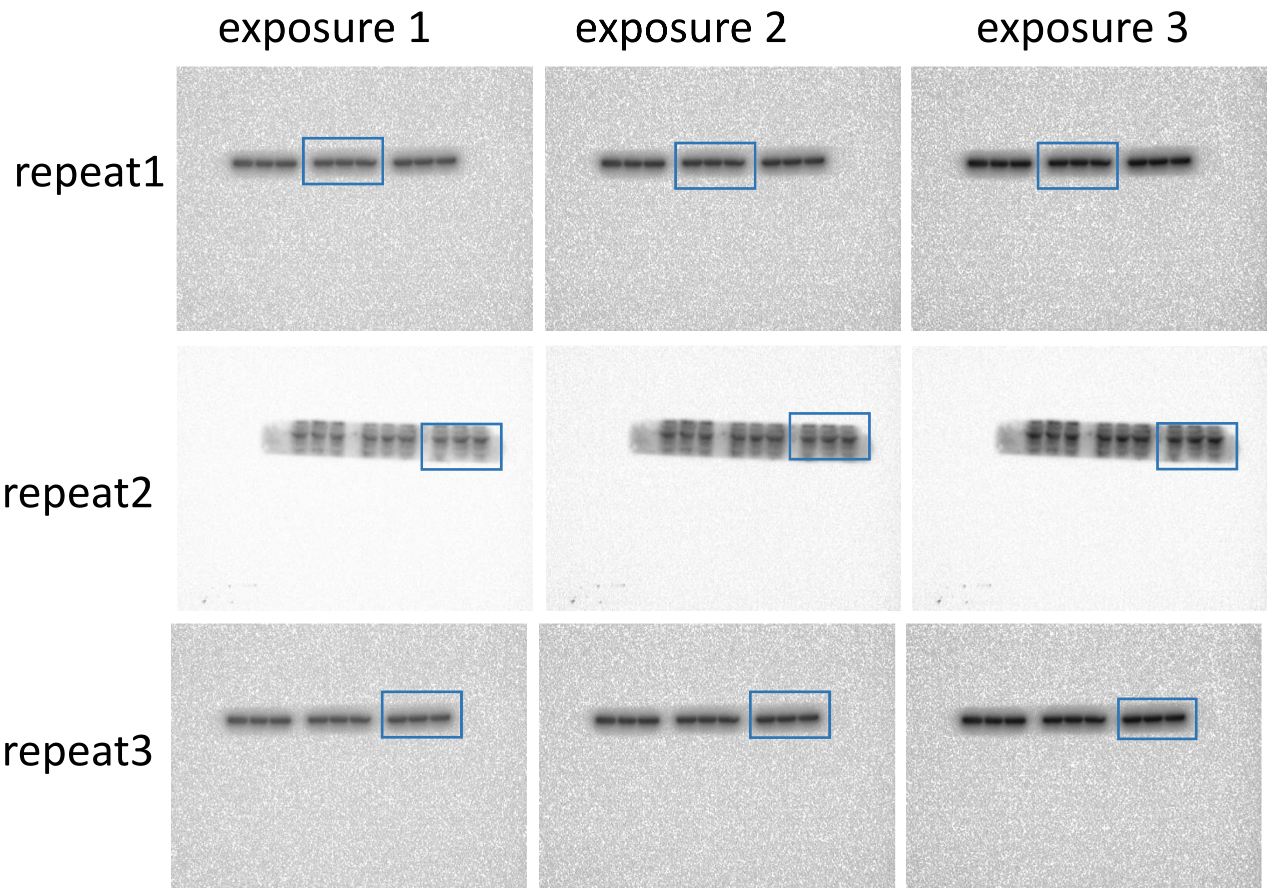

Figure 1

Figure 1e CAOV3
HERPUD1

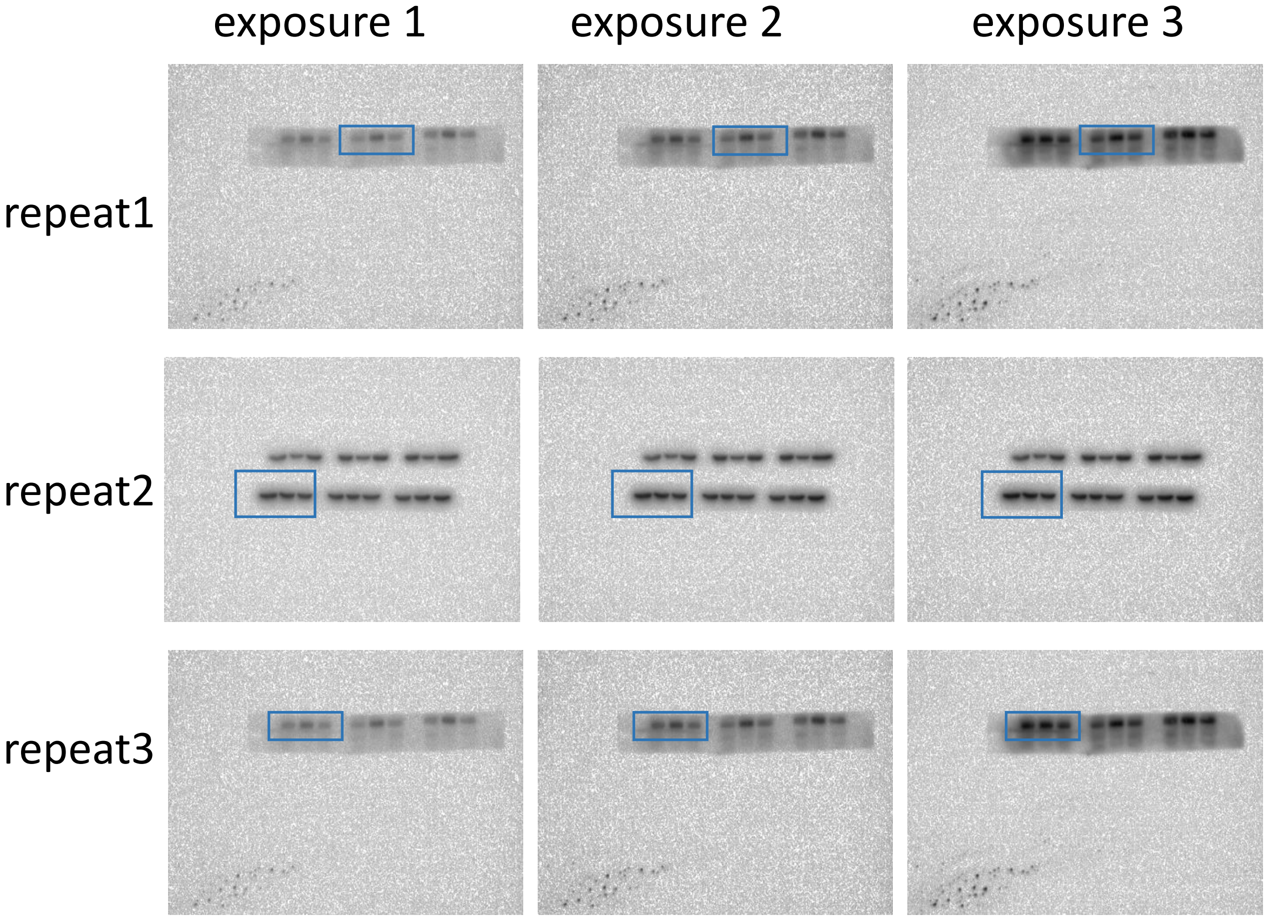

Figure 1

Figure 1e CAOV3
GAPDH

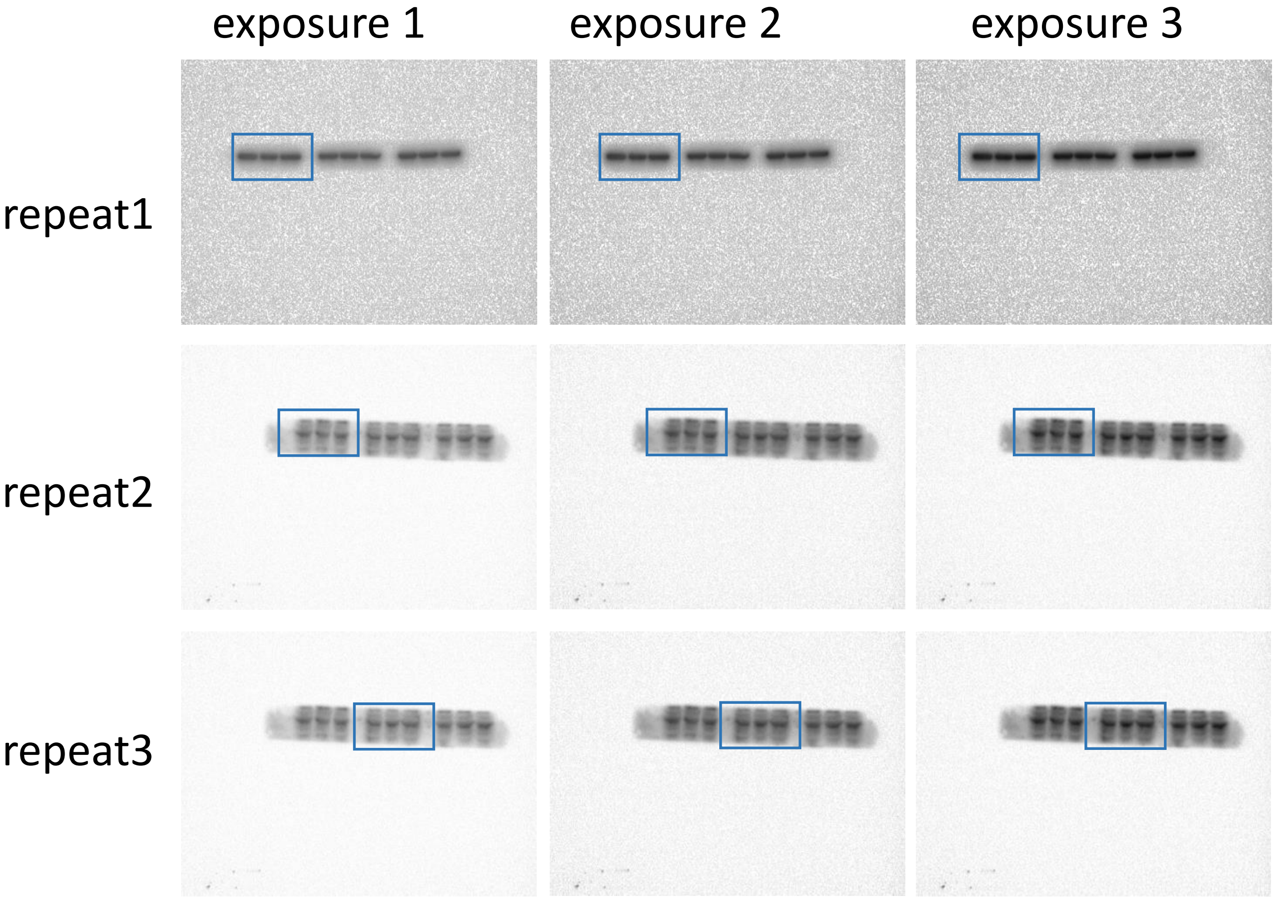

Figure 5

Figure 5b
HERPUD1

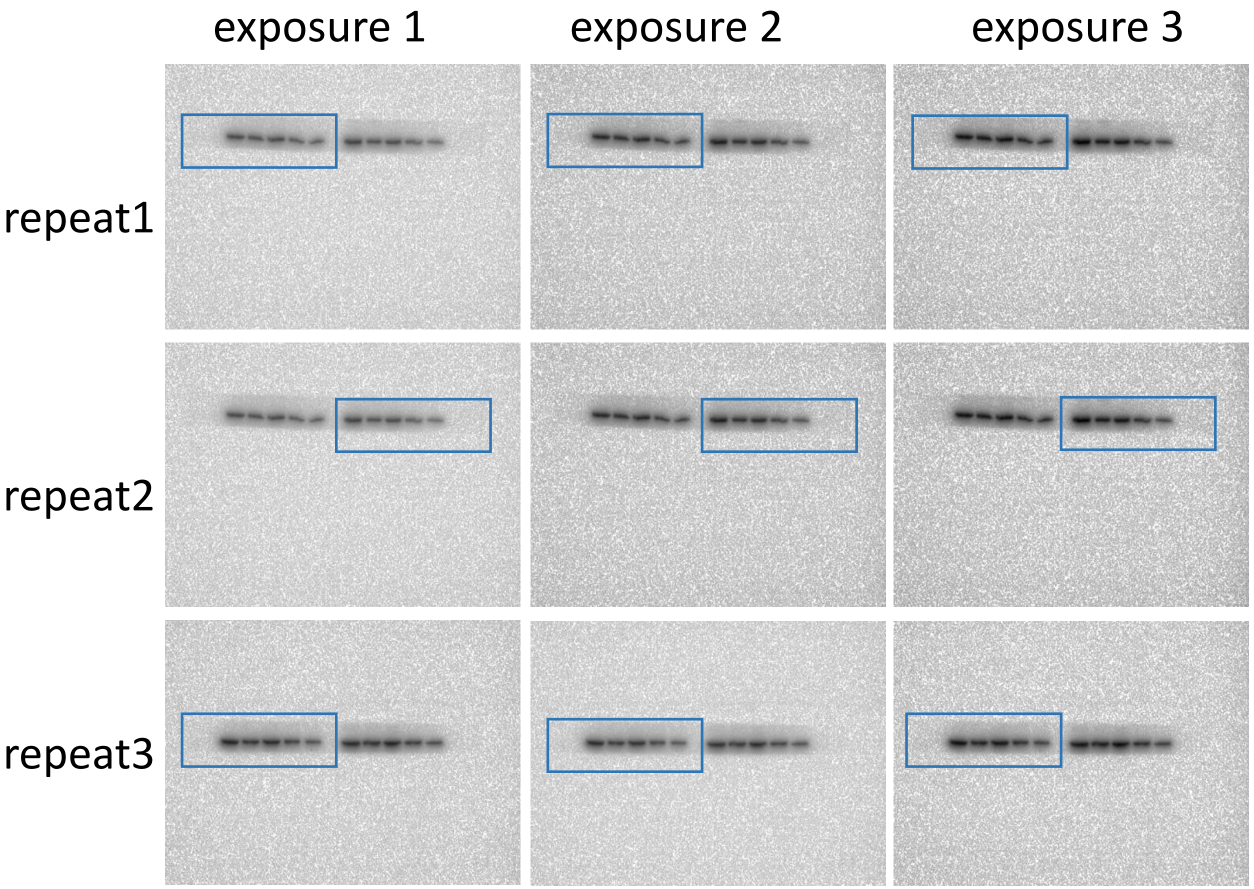

Figure 5

Figure 5b
GAPDH

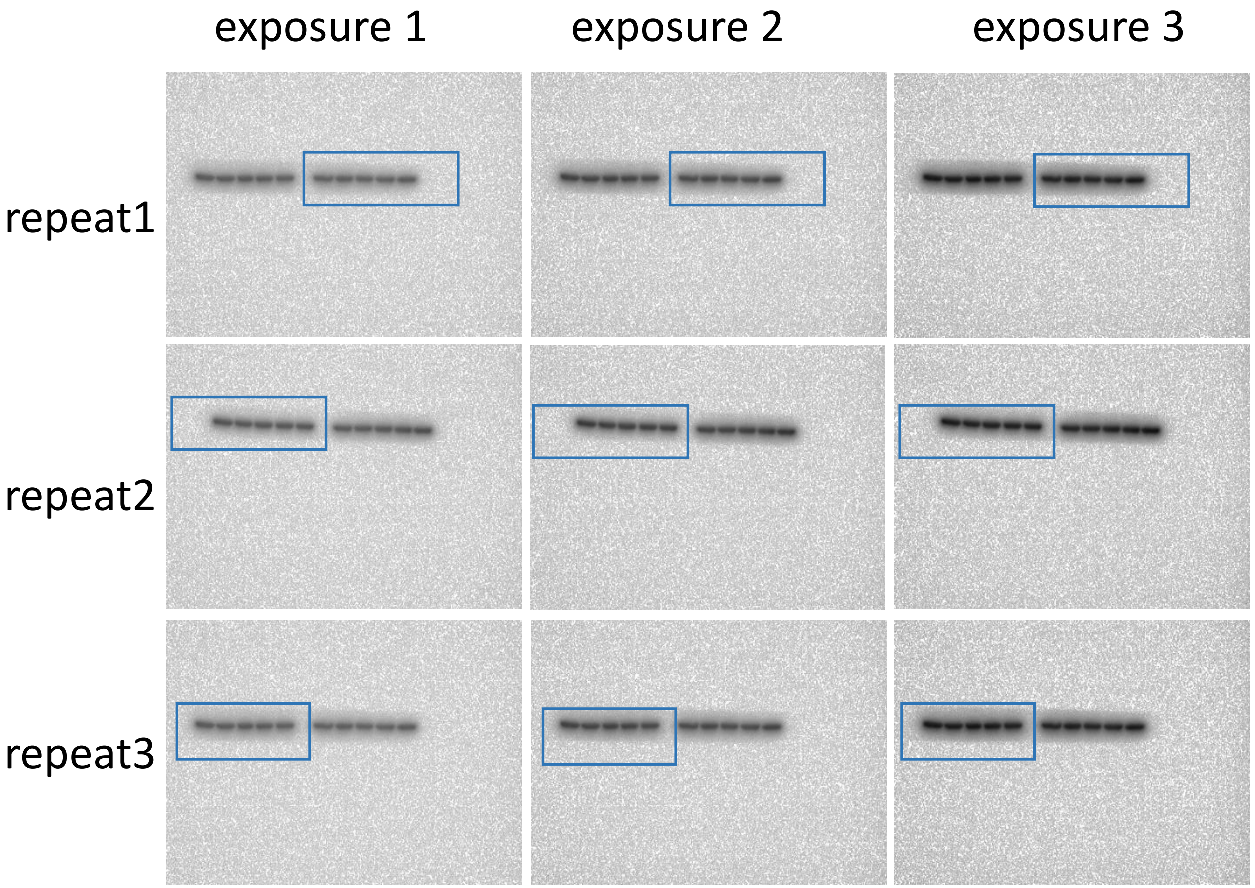

Figure 5

Figure 5e CAOV3
HERPUD1

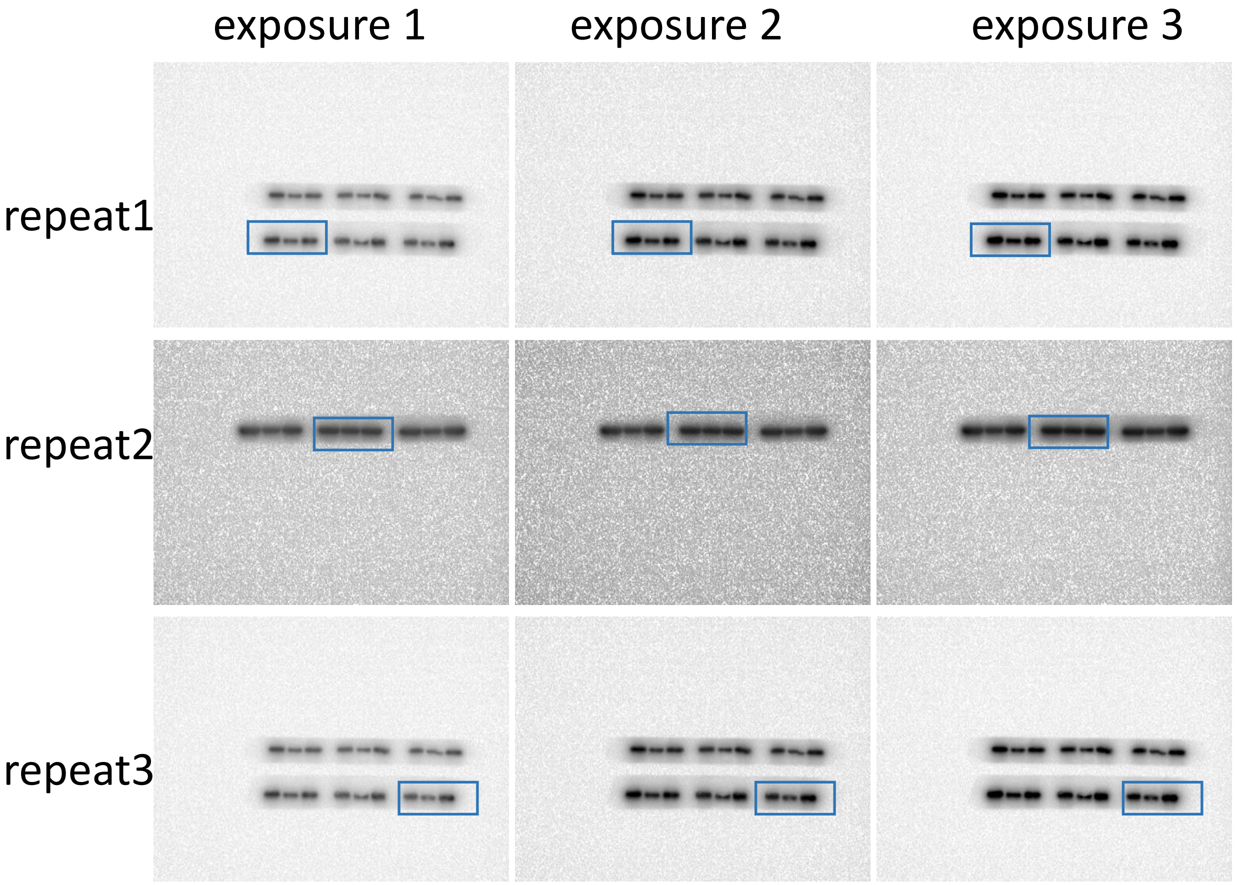

Figure 5

Figure 5e CAOV3
GAPDH

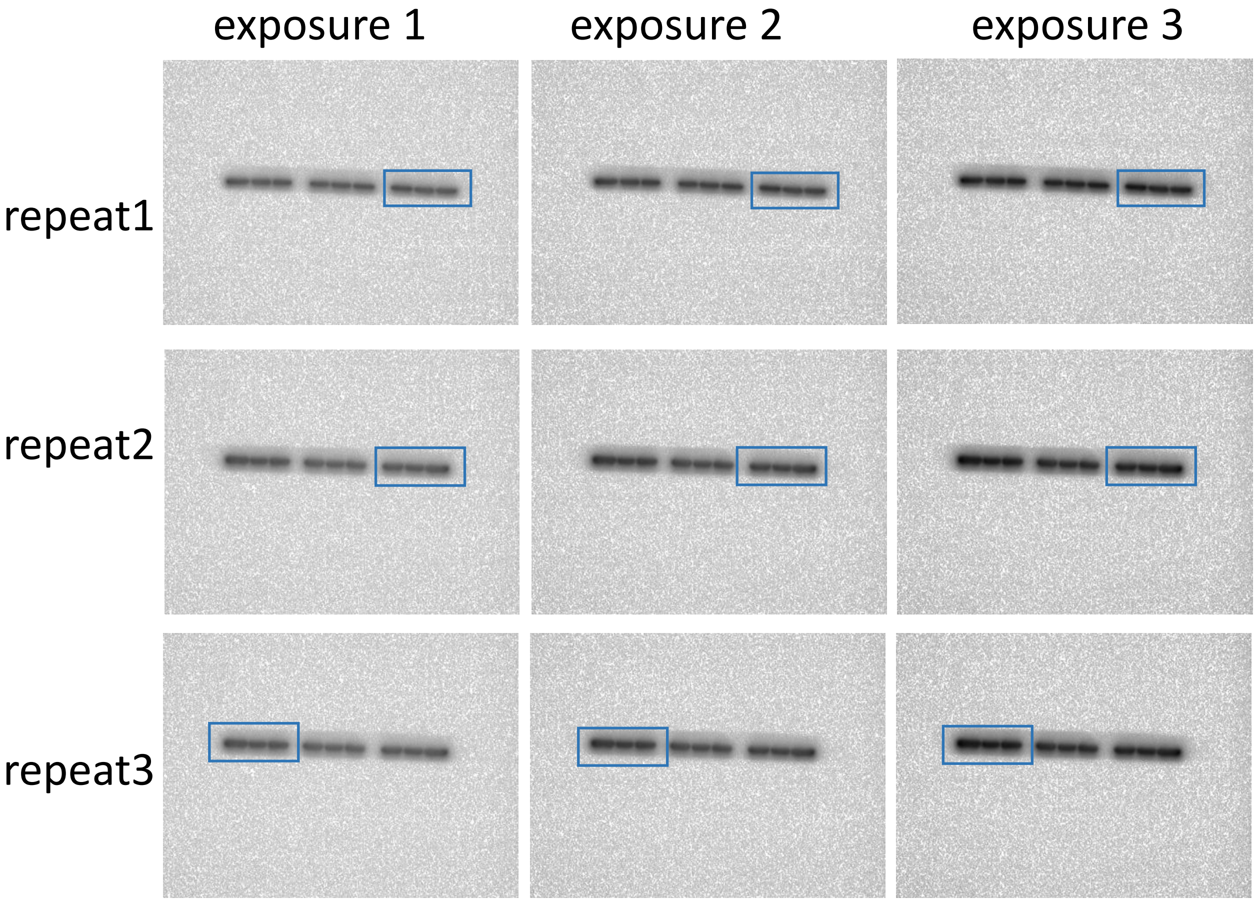

Figure 5

Figure 5e SKOV3
HERPUD1

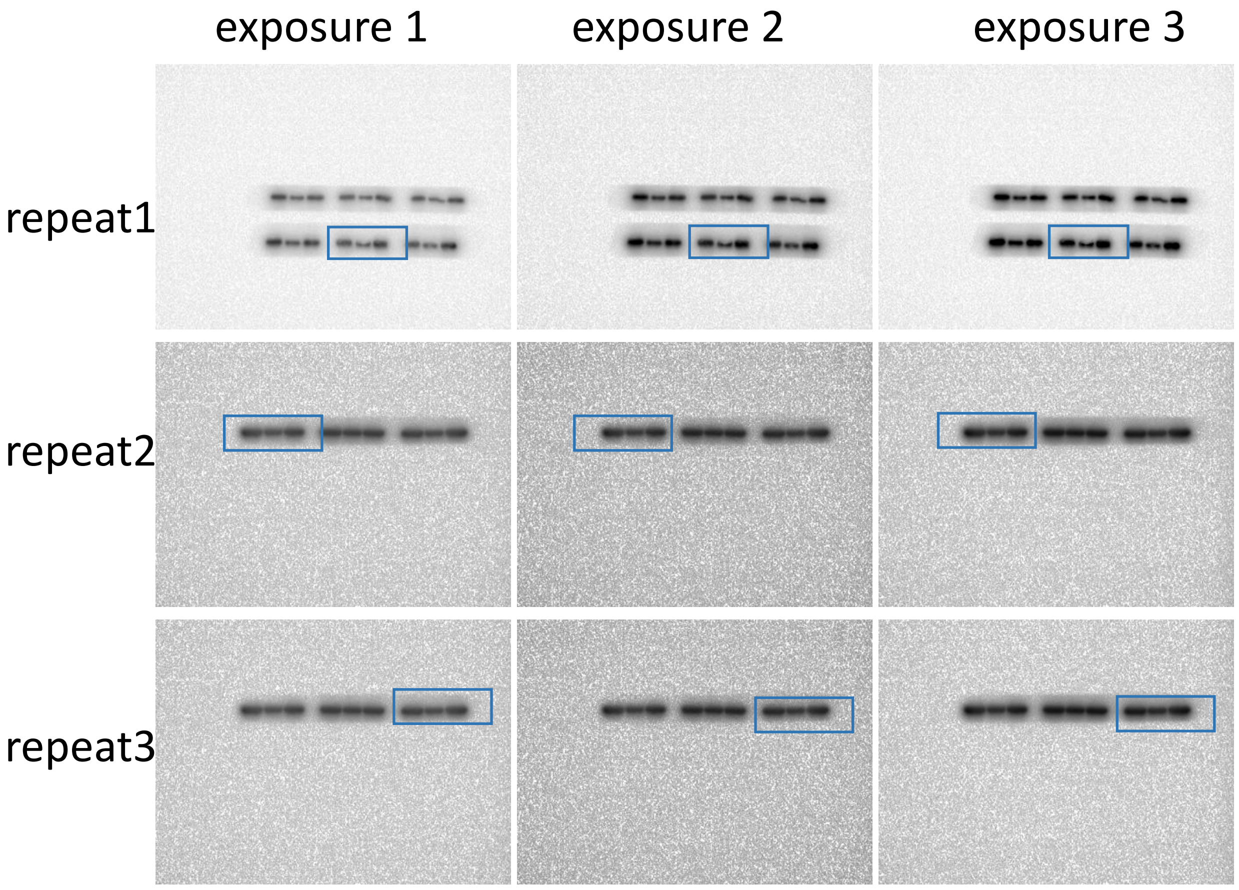

Figure 5

Figure 5e SKOV3
GAPDH

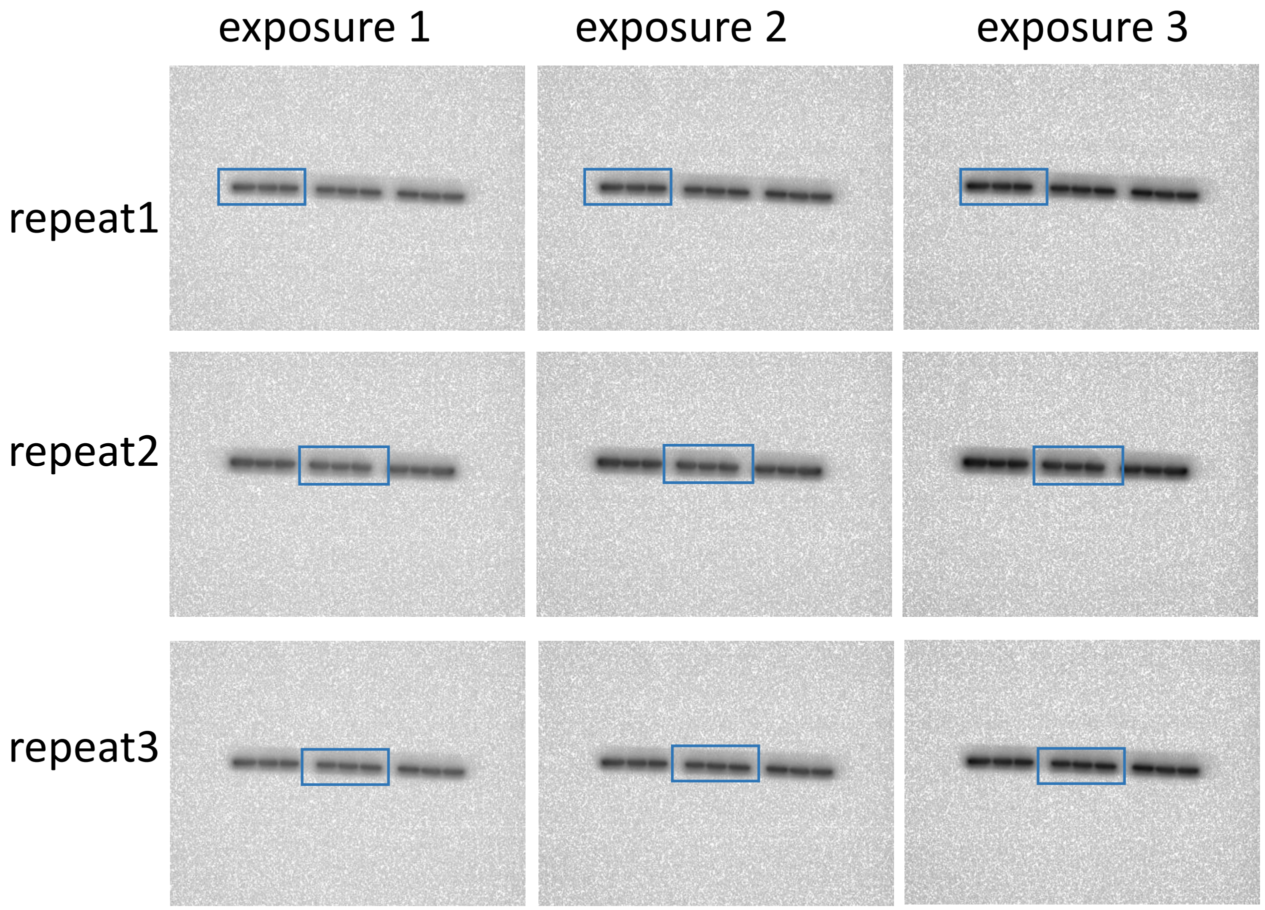

Figure 6

Figure 6f CAOV3
CyclinD1

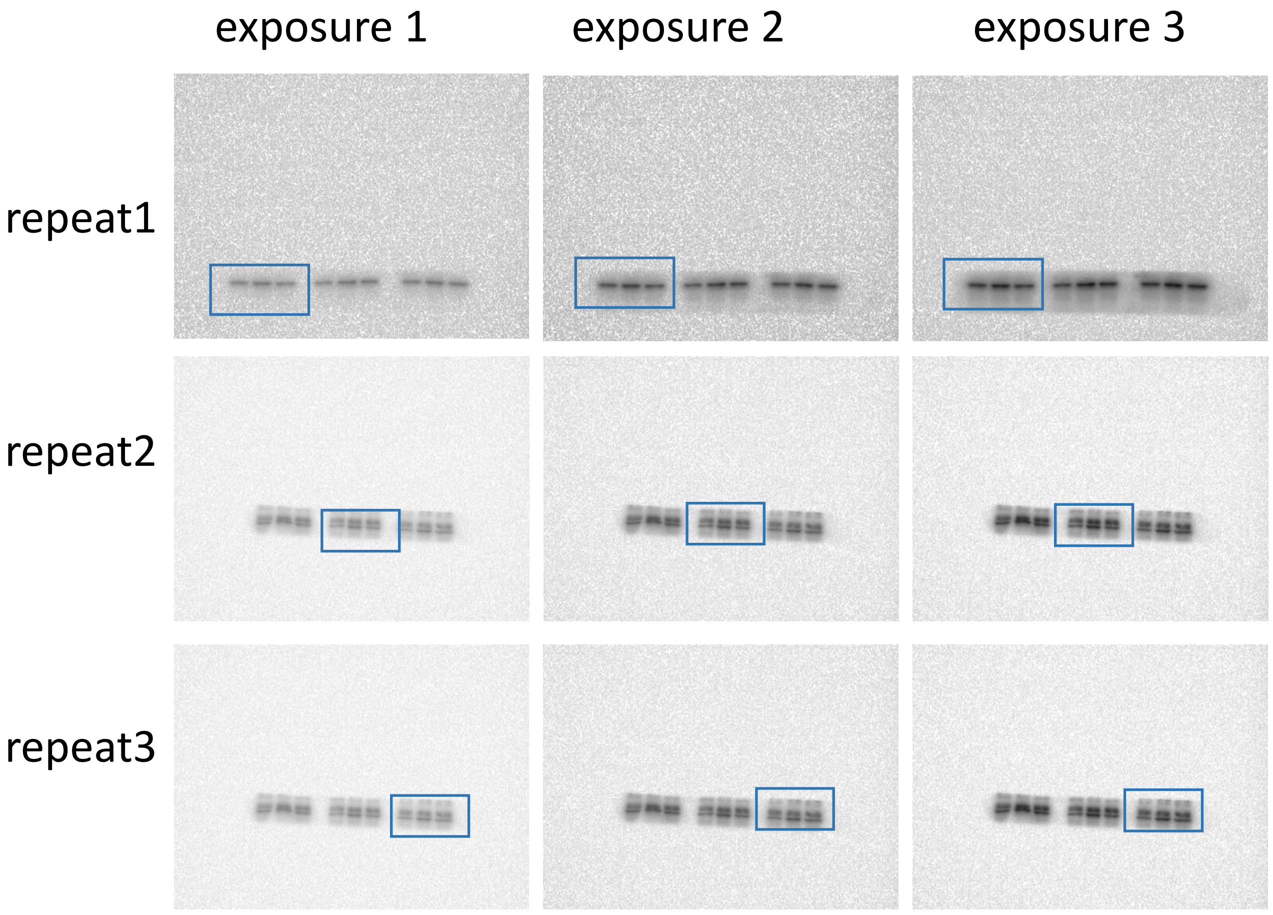

Figure 6

Figure 6f CAOV3
PCNA

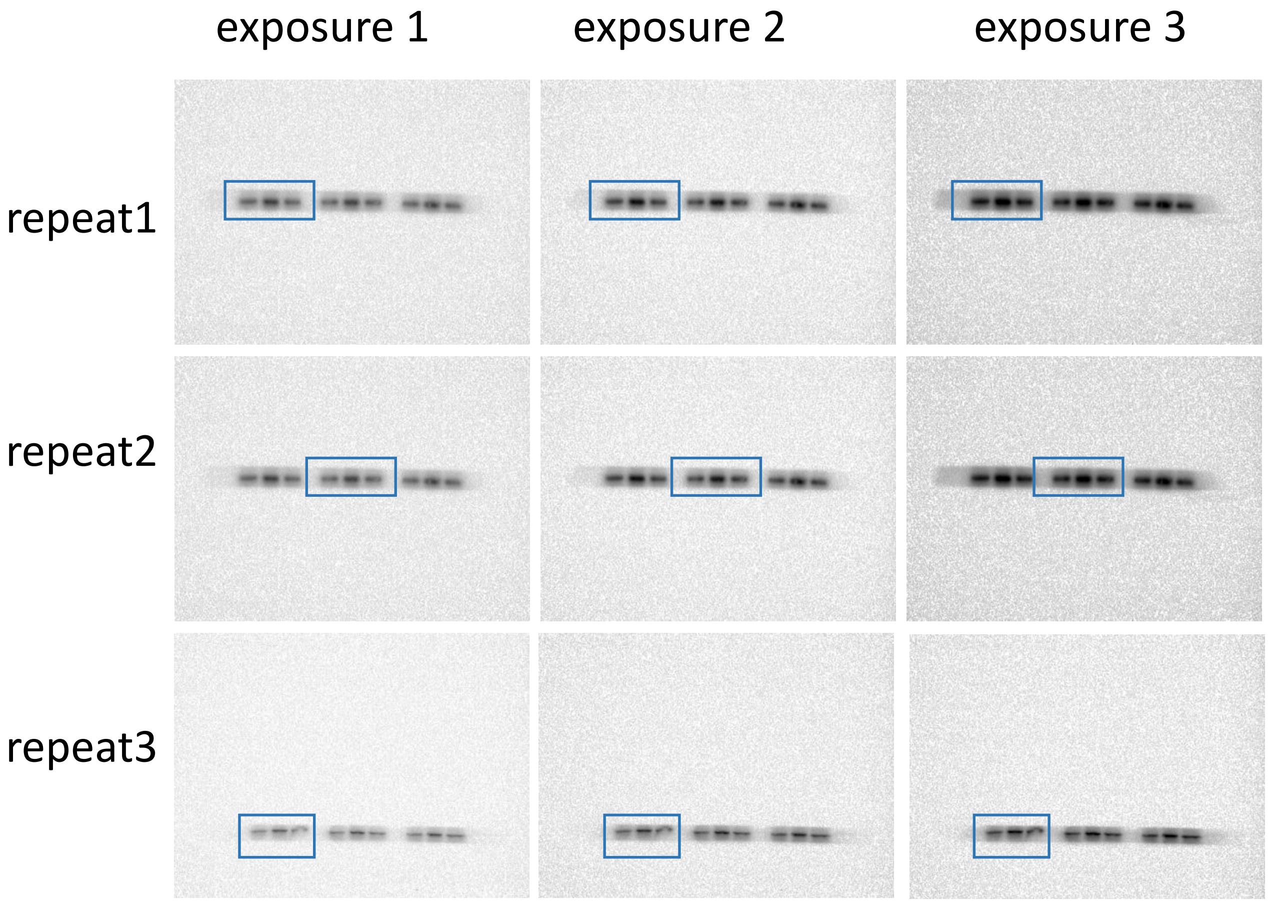

Figure 6

Figure 6f CAOV3
cleaved-Caspase3

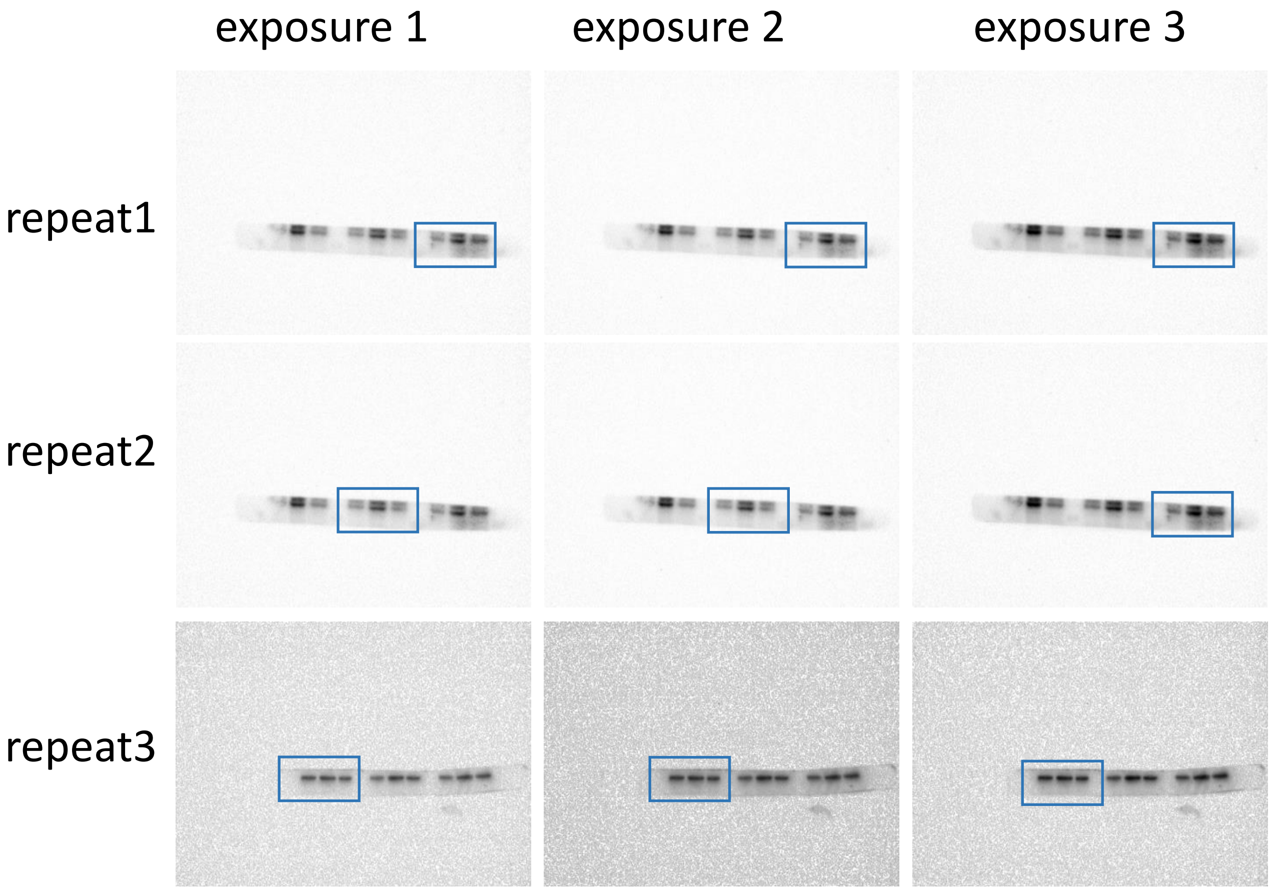

Figure 6f CAOV3
cleaved-Caspase12

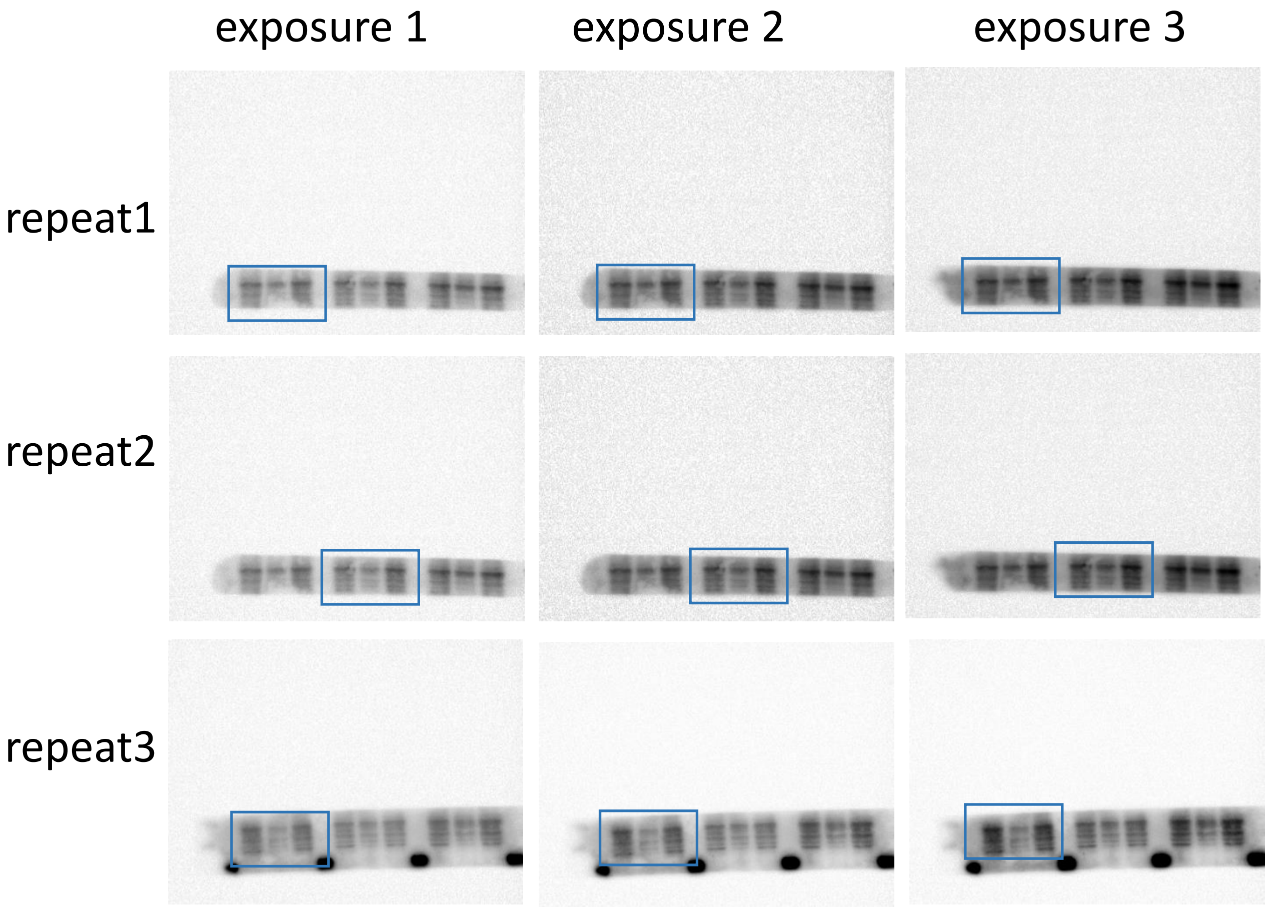

Figure 6

Figure 6f CAOV3
GAPDH

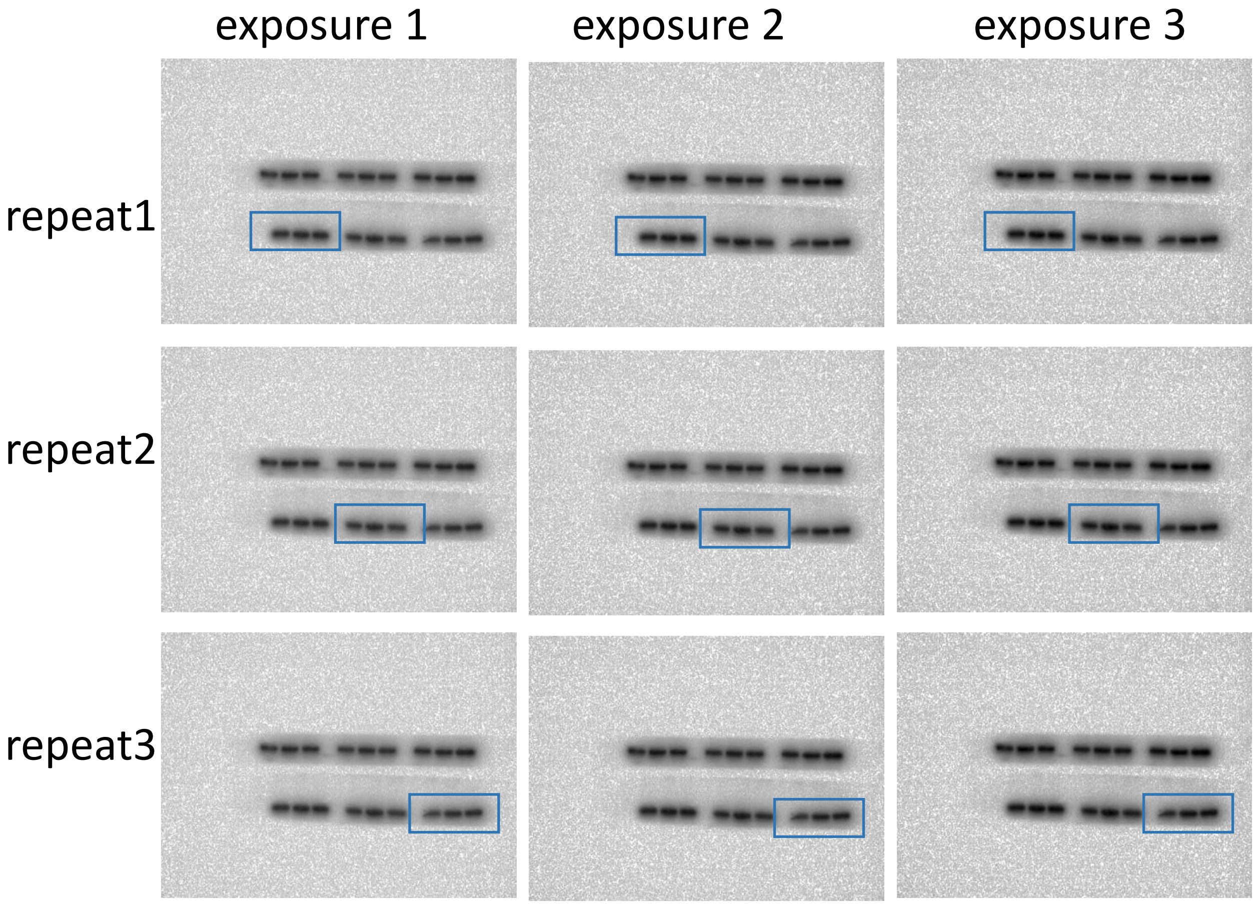

Figure 6

Figure 6f SKOV3
CyclinD1

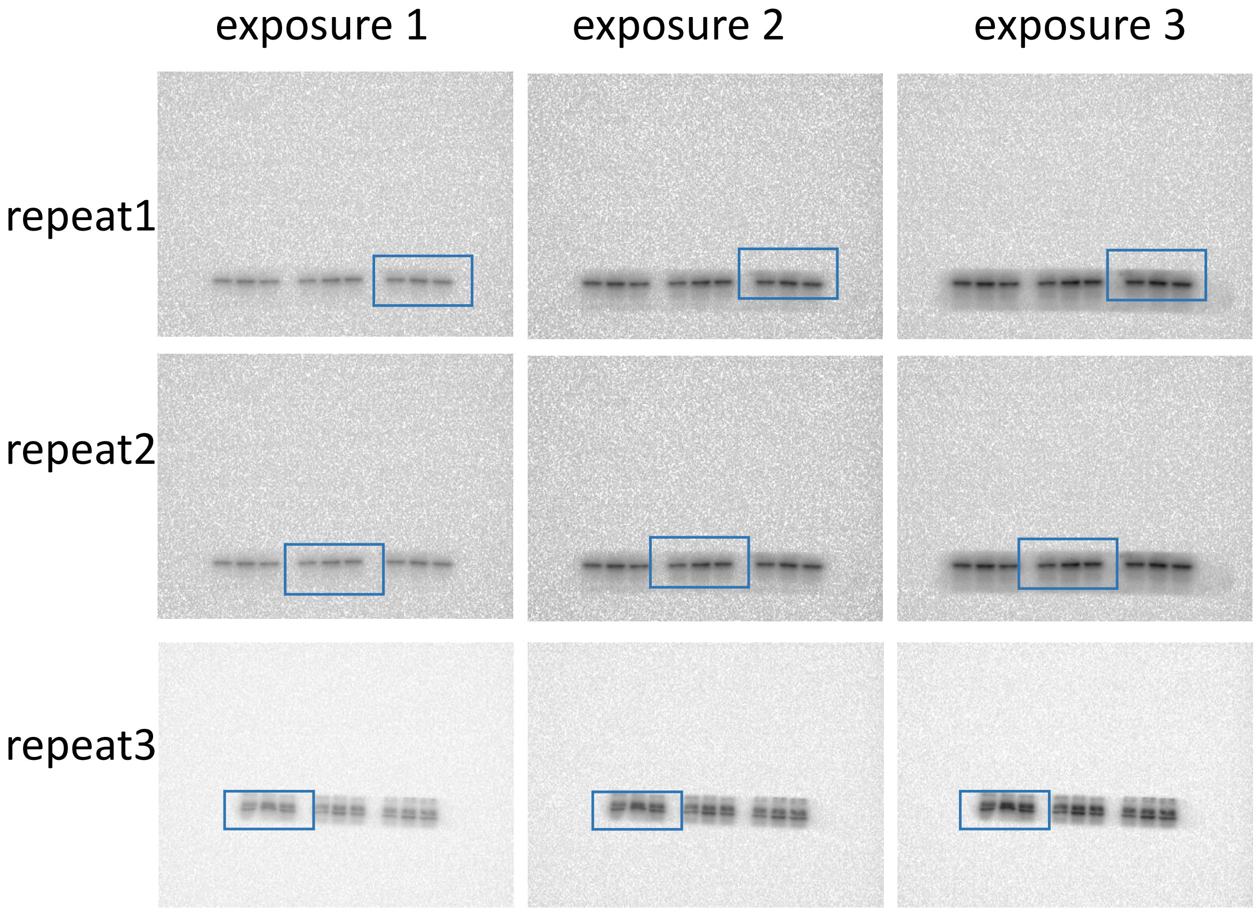

Figure 6

Figure 6f SKOV3
PCNA

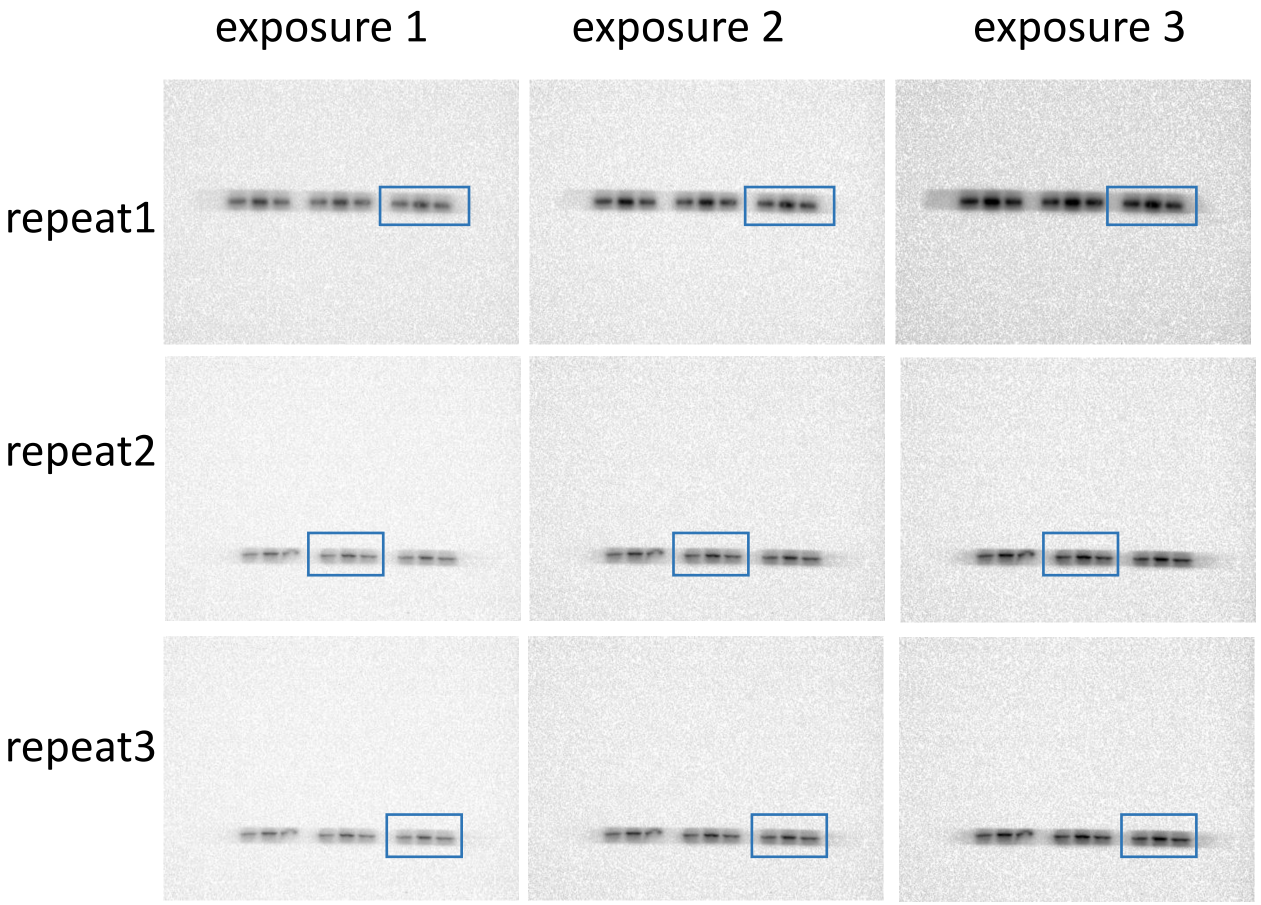

Figure 6

Figure 6f SKOV3
cleaved-Caspase3

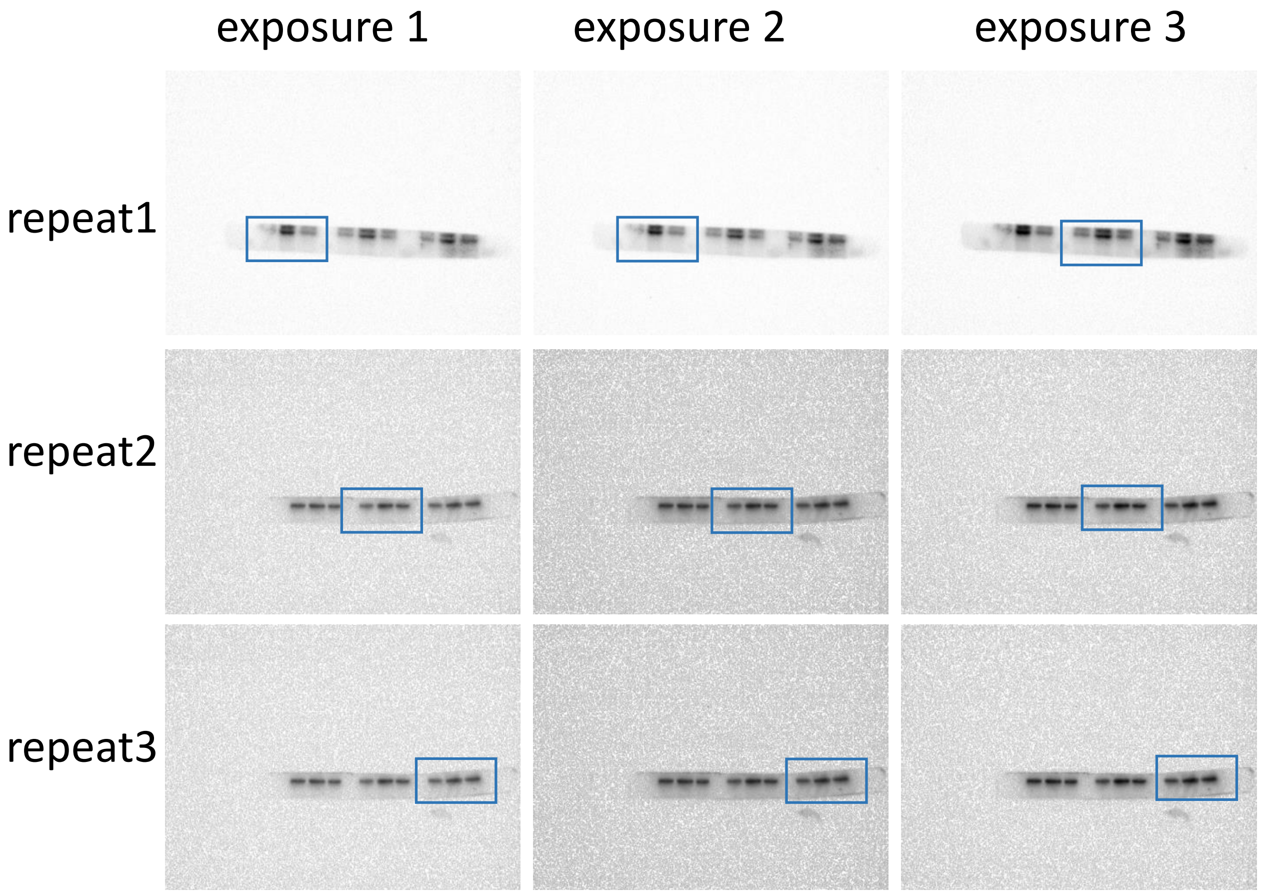

Figure 6

Figure 6f SKOV3
cleaved-Caspase12

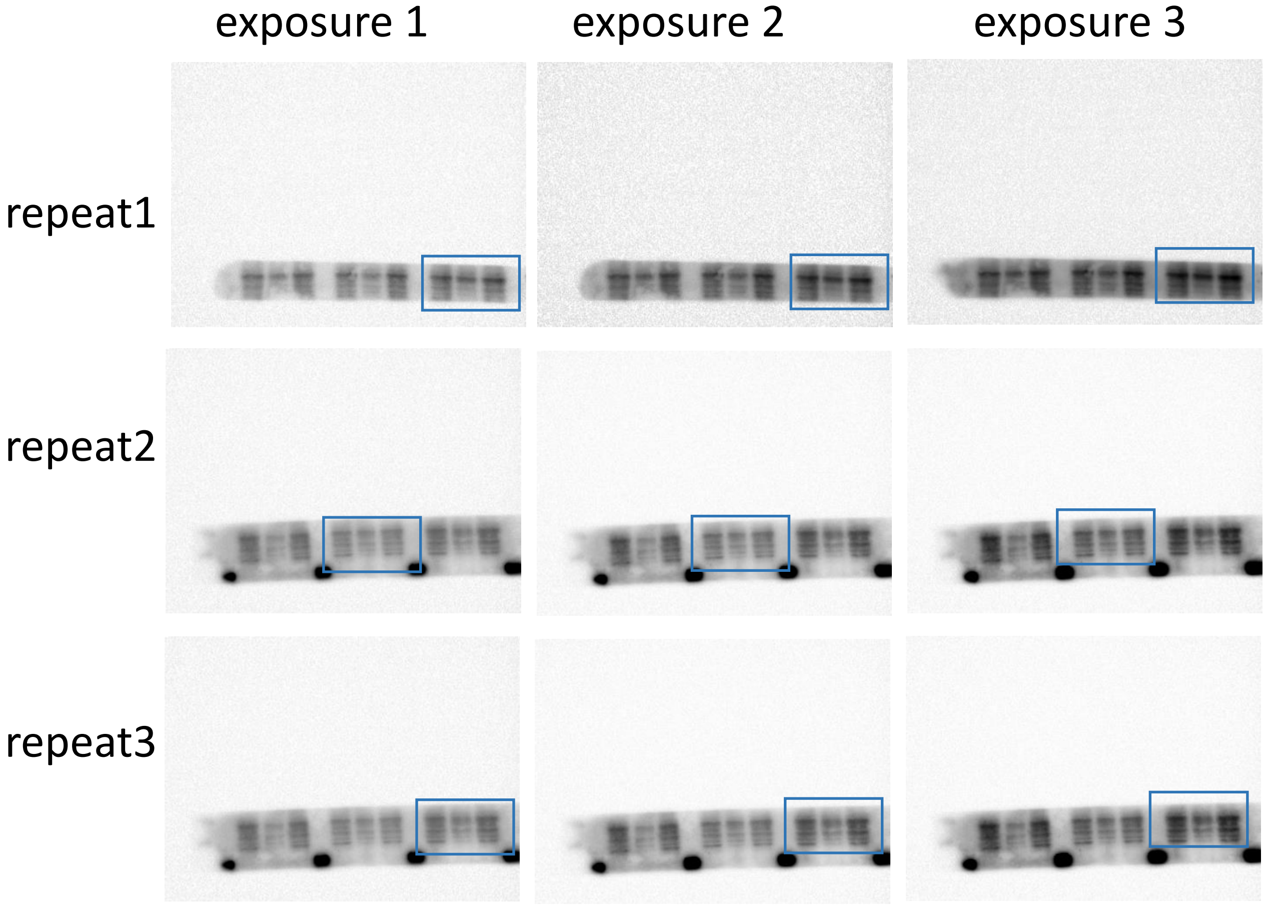

Figure 6

Figure 6f SKOV3
GAPDH

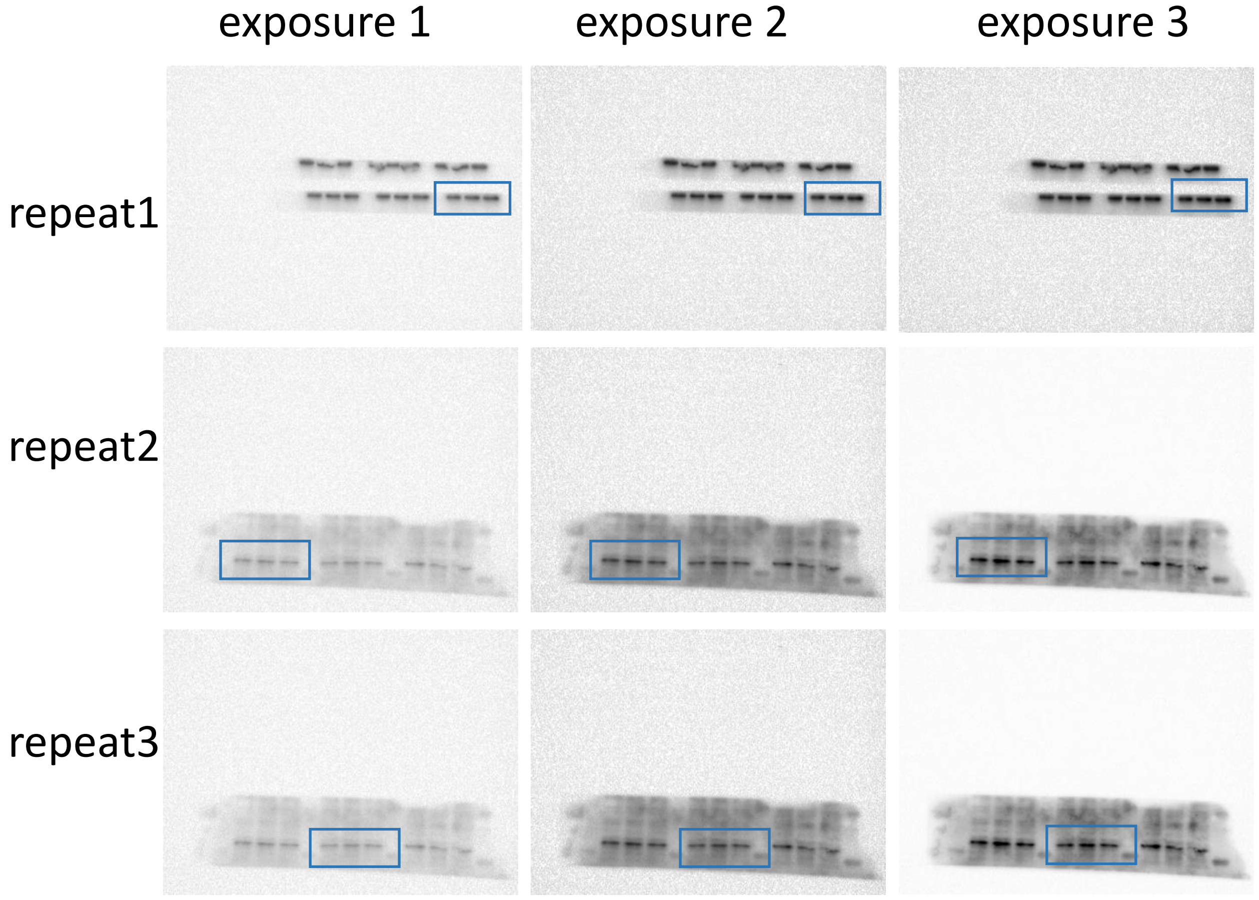

Figure 7

Figure 7a CAOV3
p27

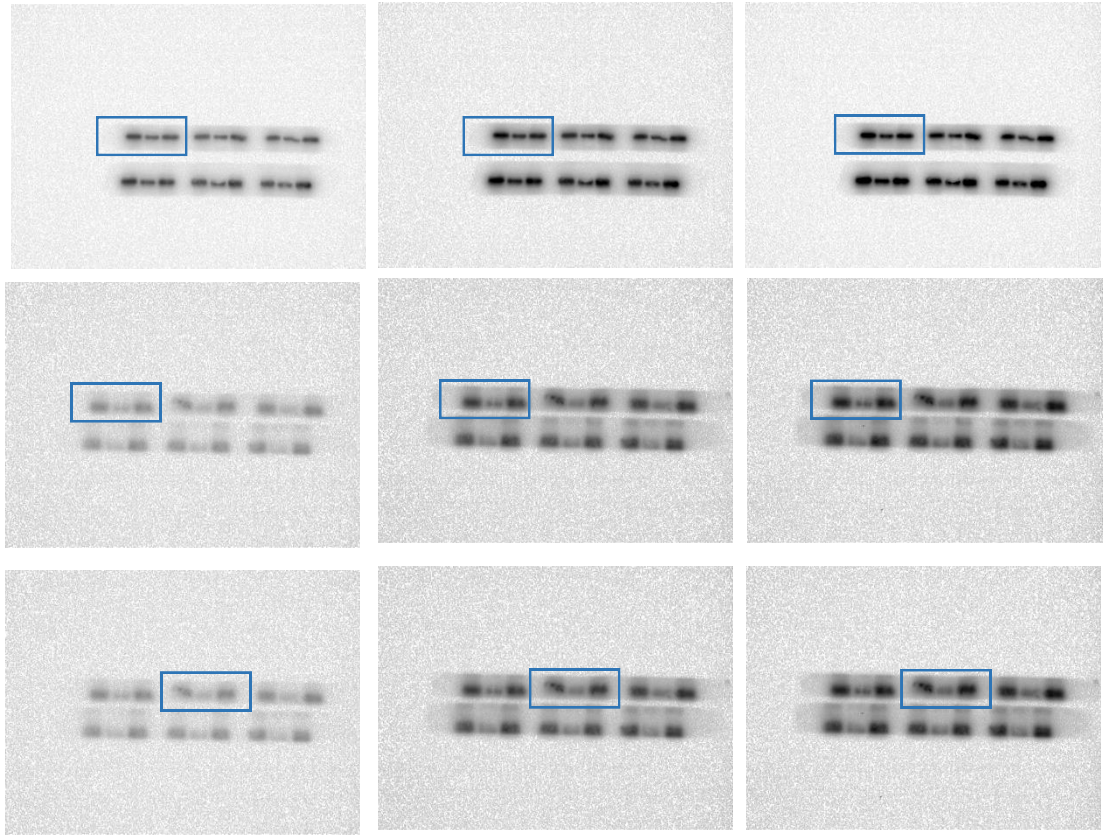

Figure 7

Figure 7a CAOV3
p62

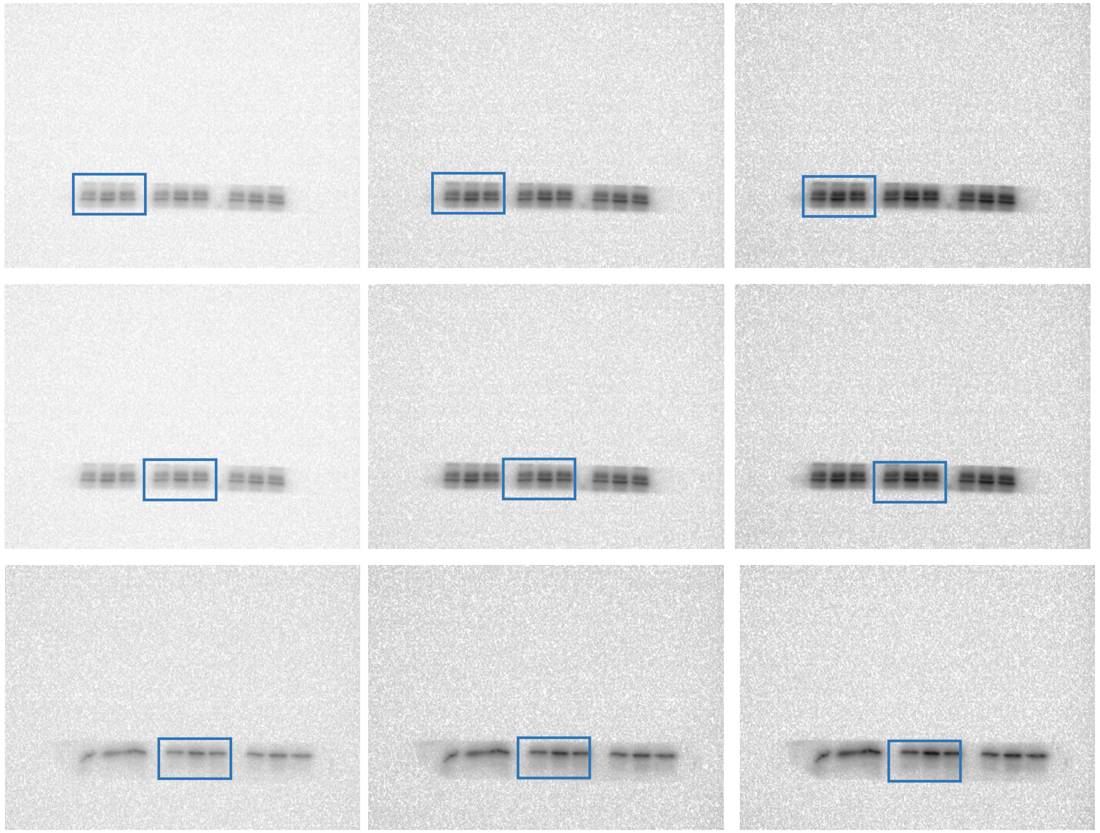

Figure 7

Figure 7a CAOV3
Atg5

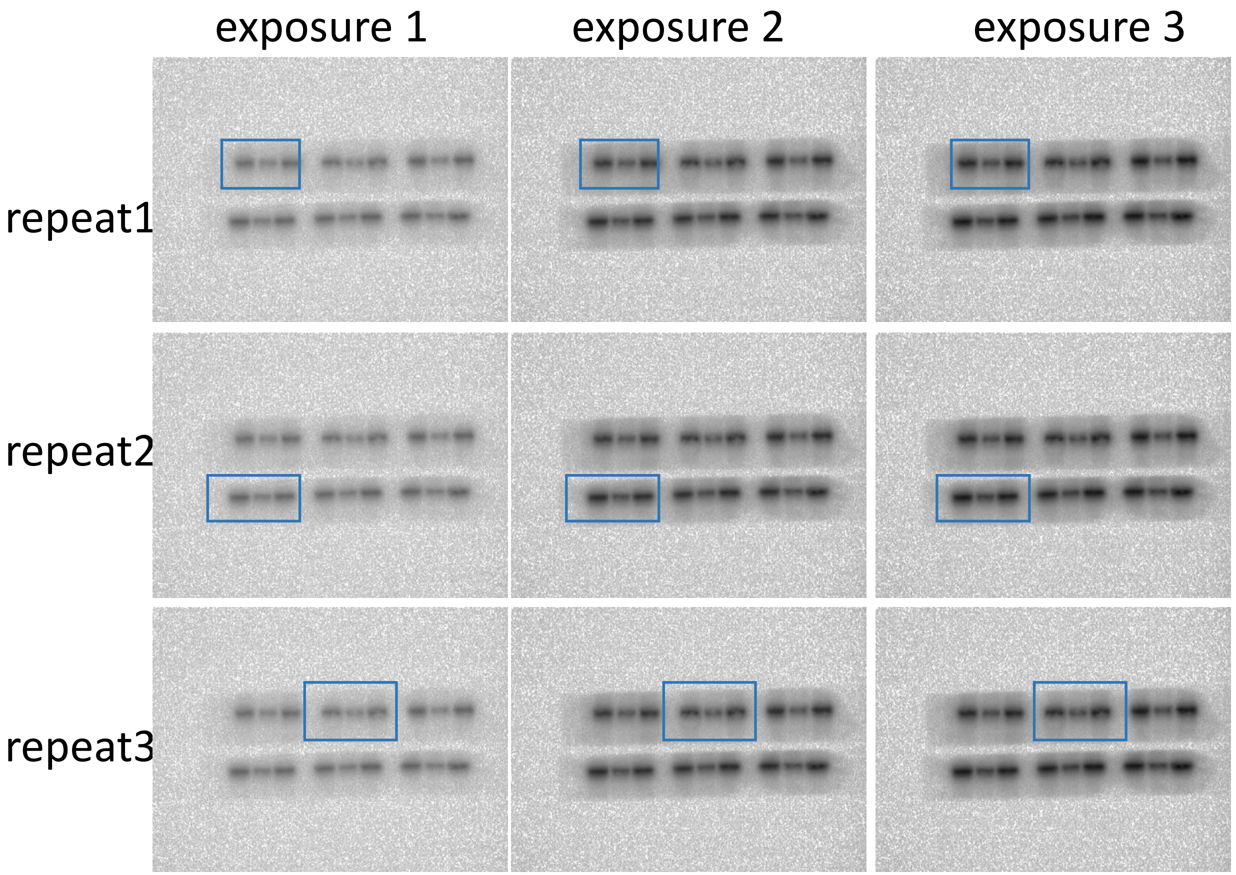

Figure 7

Figure 7a CAOV3
Beclin1

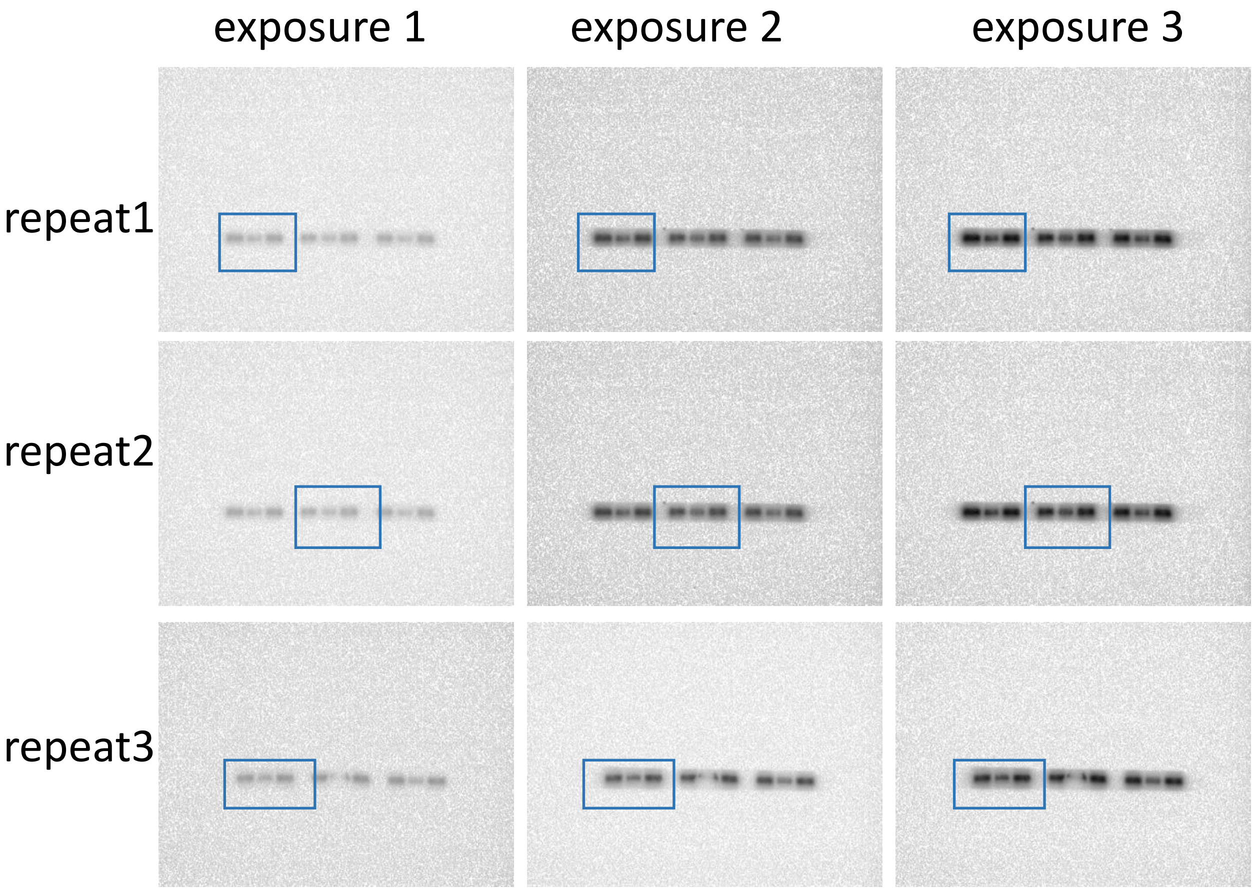

Figure 7

Figure 7a CAOV3
LC3

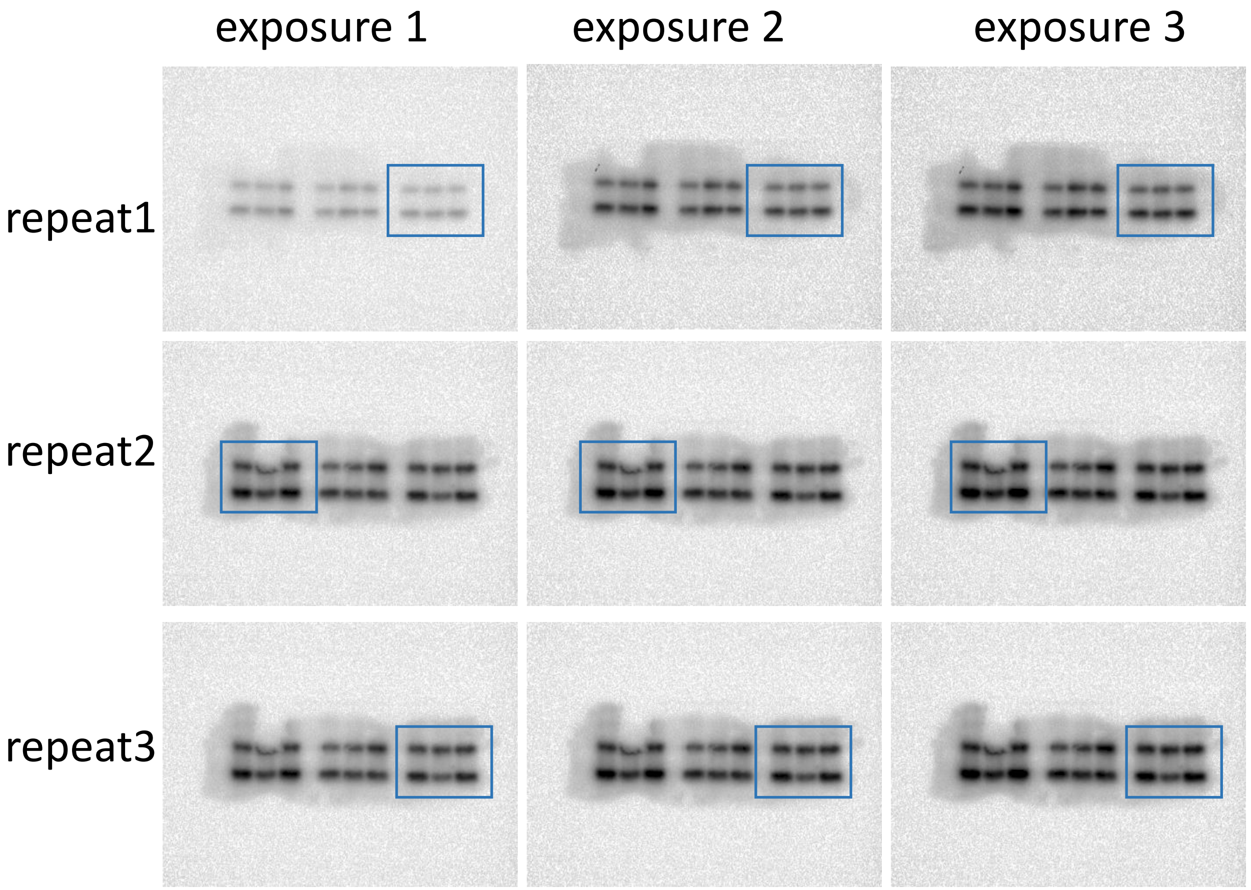

Figure 7

Figure 7a CAOV3
NCA

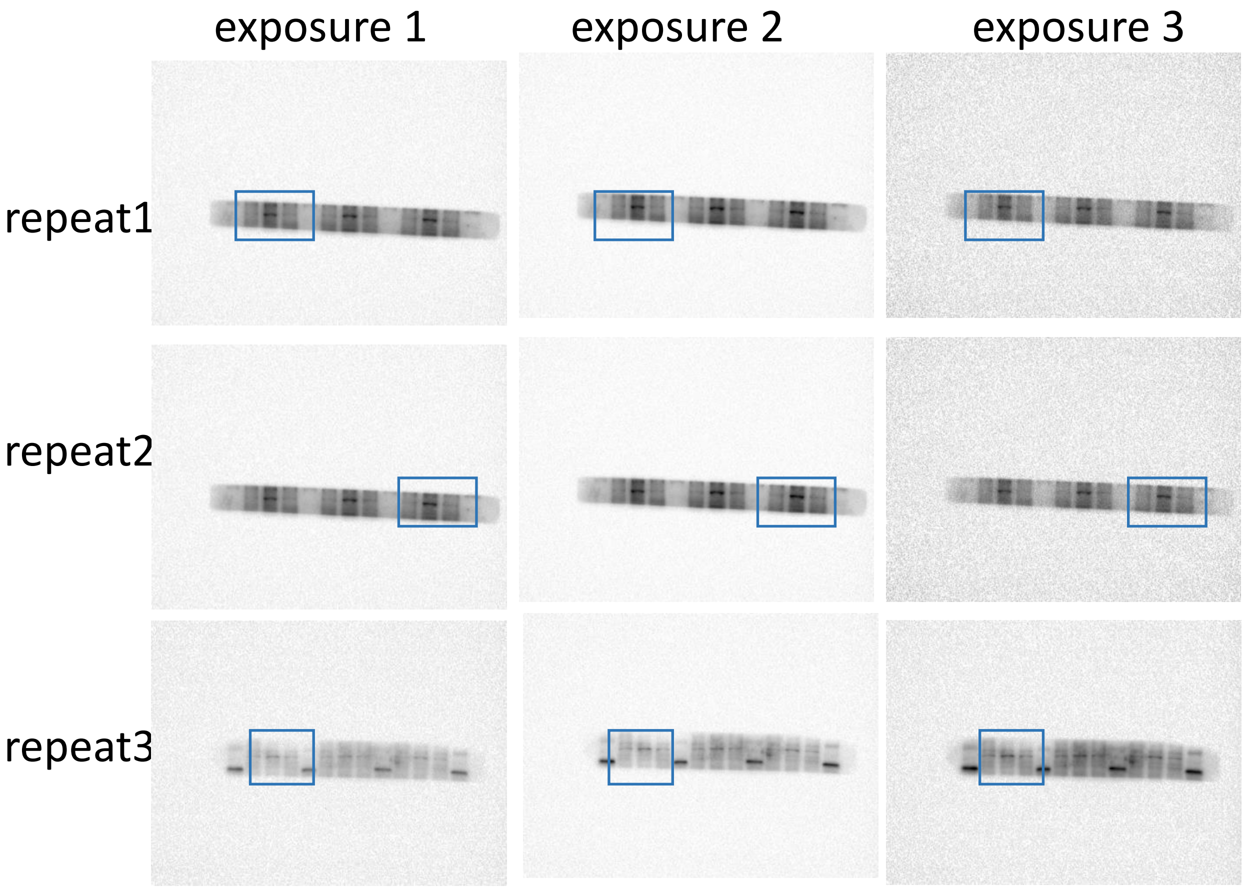

Figure 7

Figure 7a CAOV3

ECA

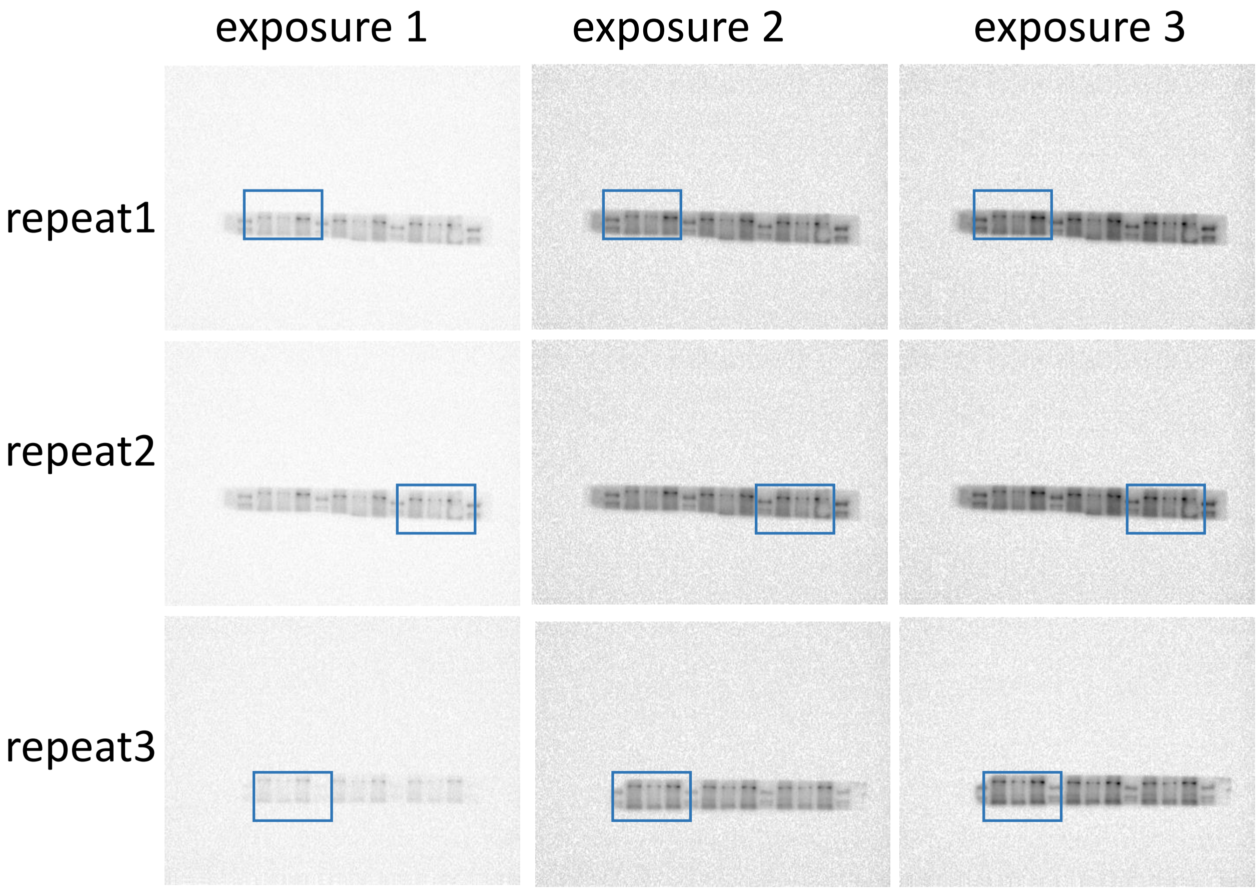

Figure 7

Figure 7a CAOV3

Timp2

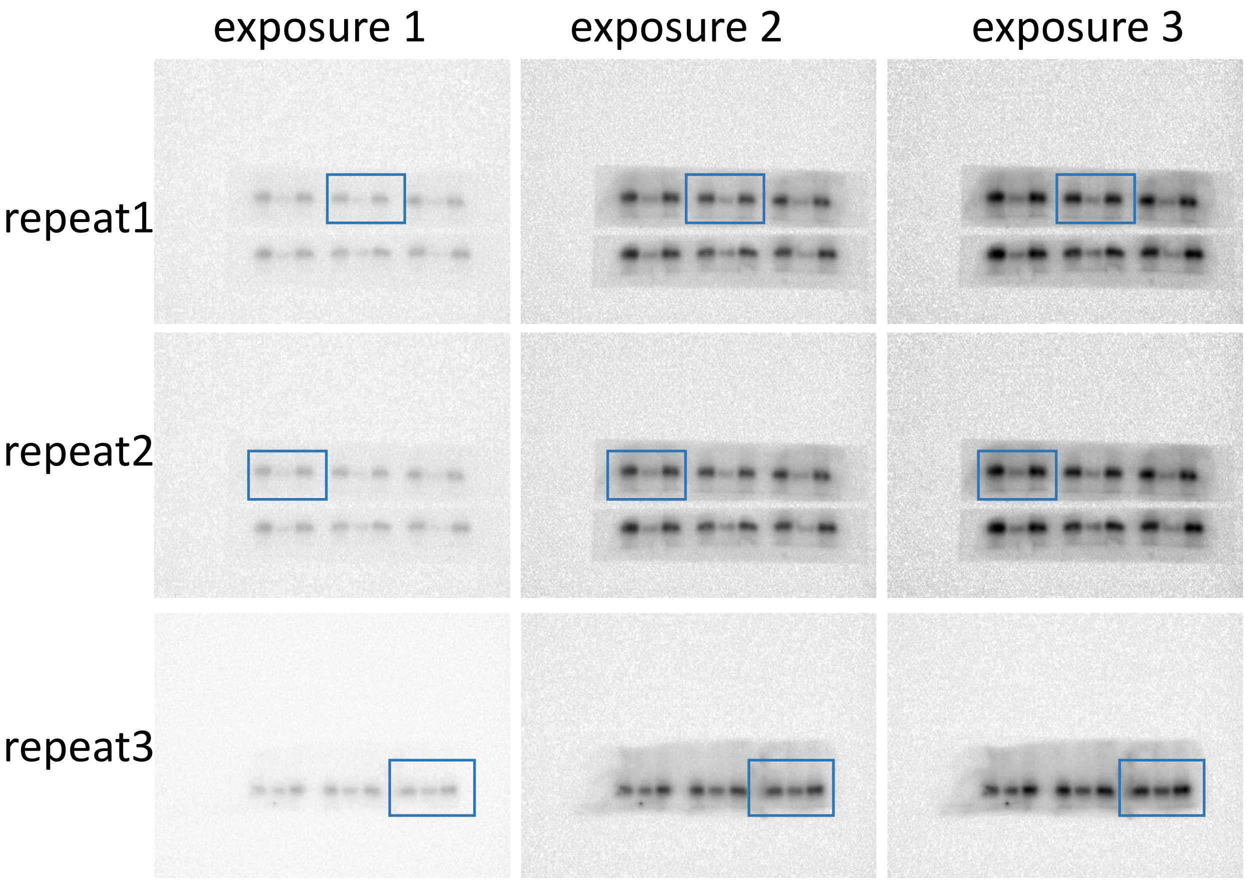

Figure 7

Figure 7a CAOV3
GRP94

repeat1
repeat2
repeat3

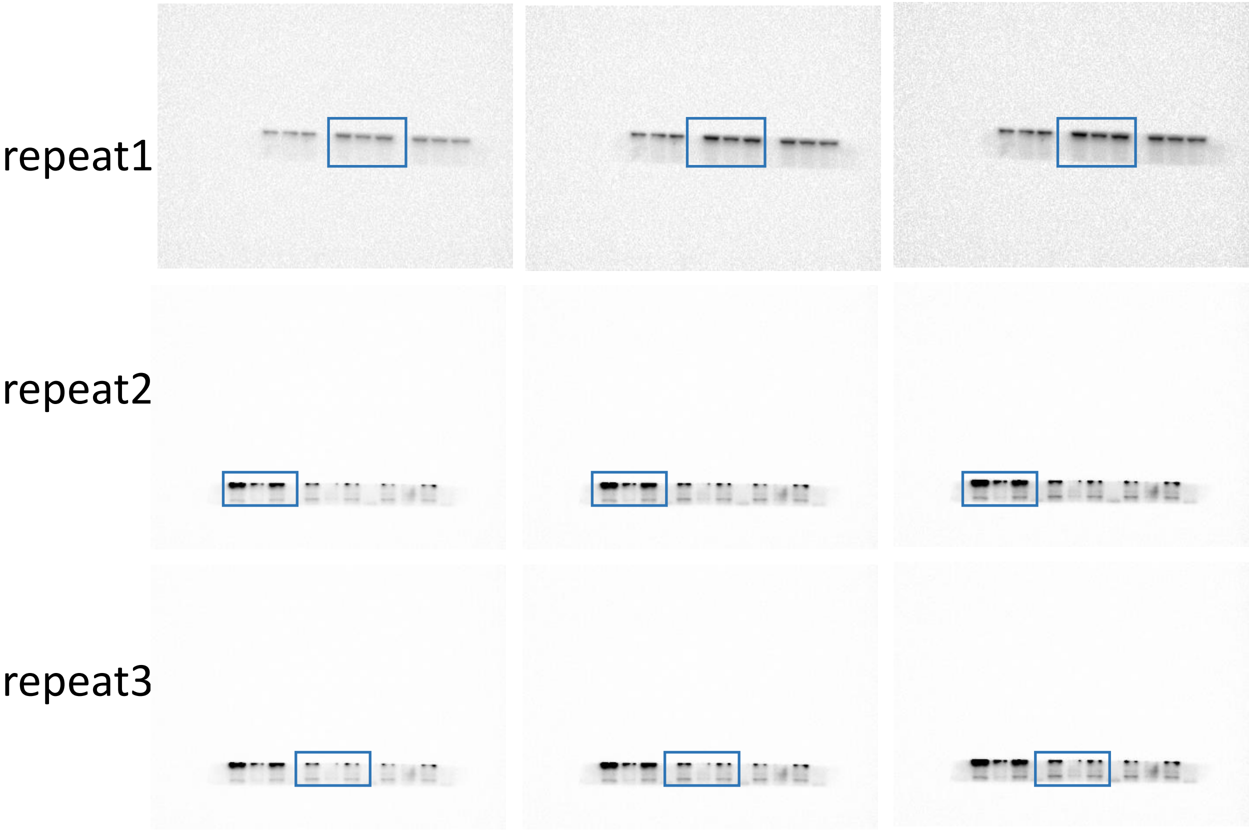

Figure 7

Figure 7a CAOV3
GRP78

exposure 1 exposure 2 exposure 3

repeat1
repeat2
repeat3

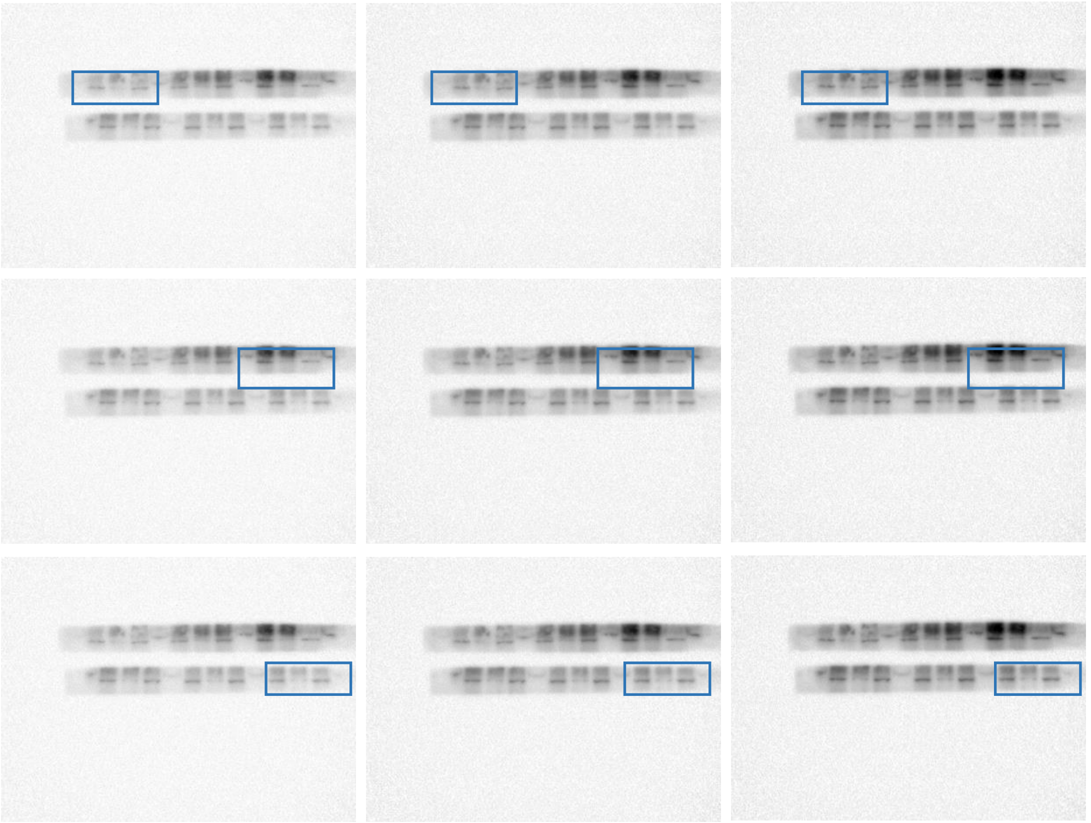

Figure 7

Figure 7a CAOV3

GAPDH

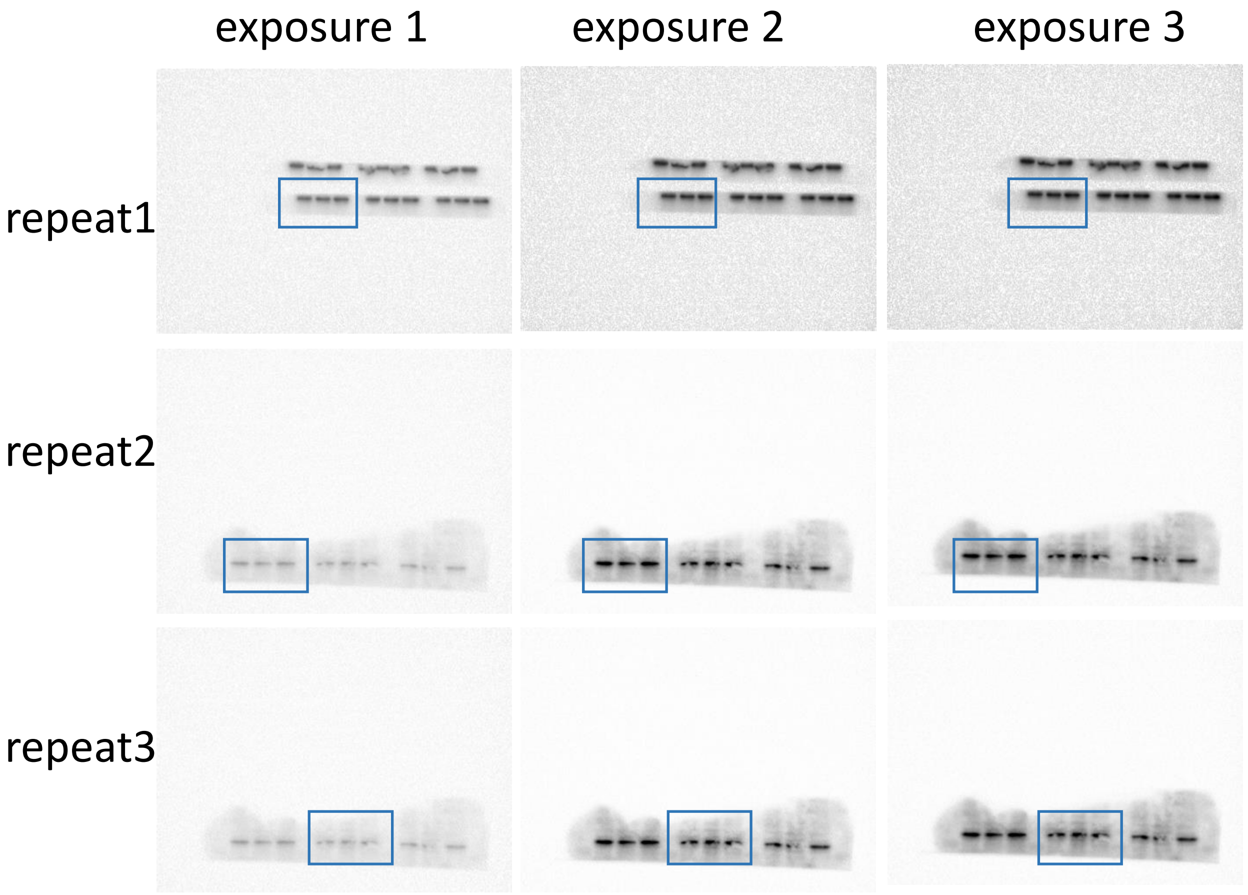

Figure 7

Figure 7a SKOV3

p27

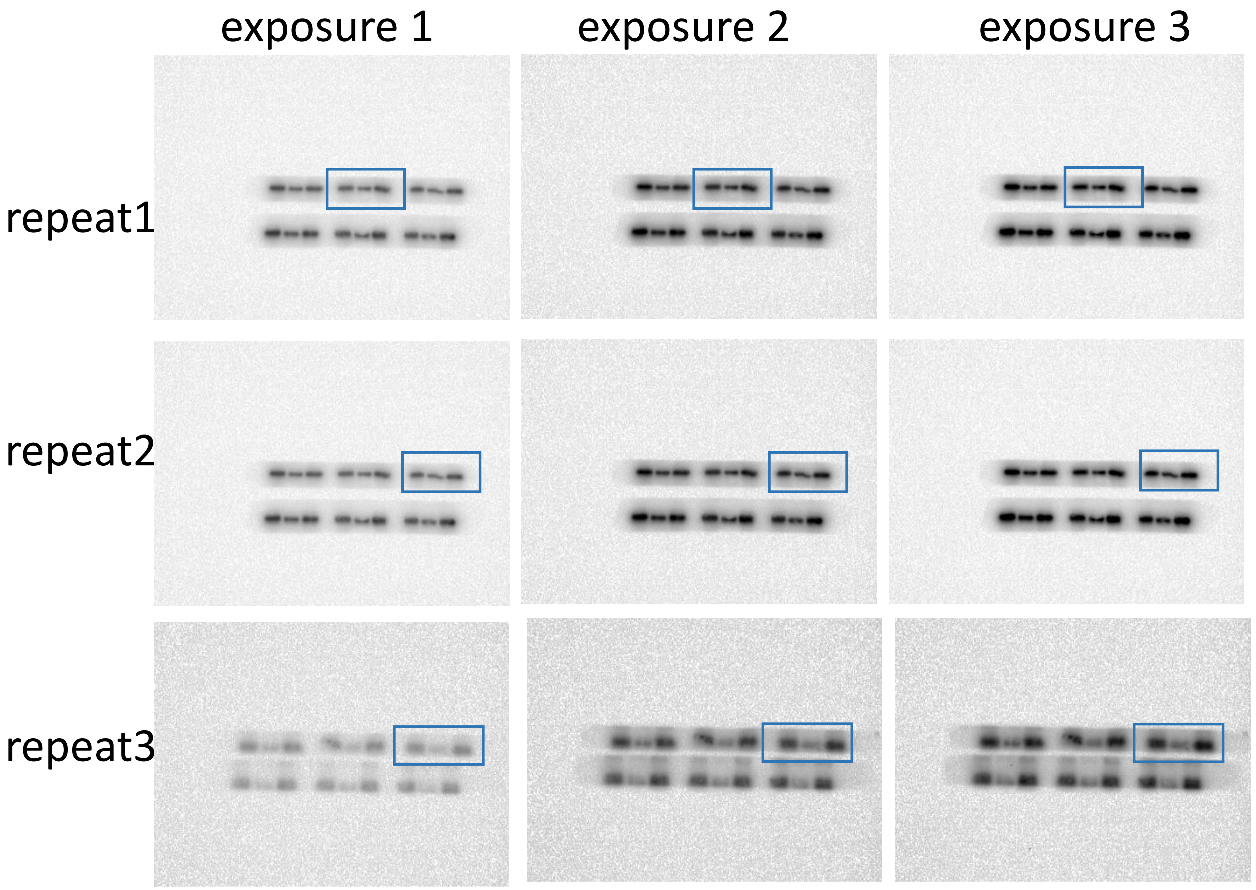

Figure 7

Figure 7a SKOV3
p62

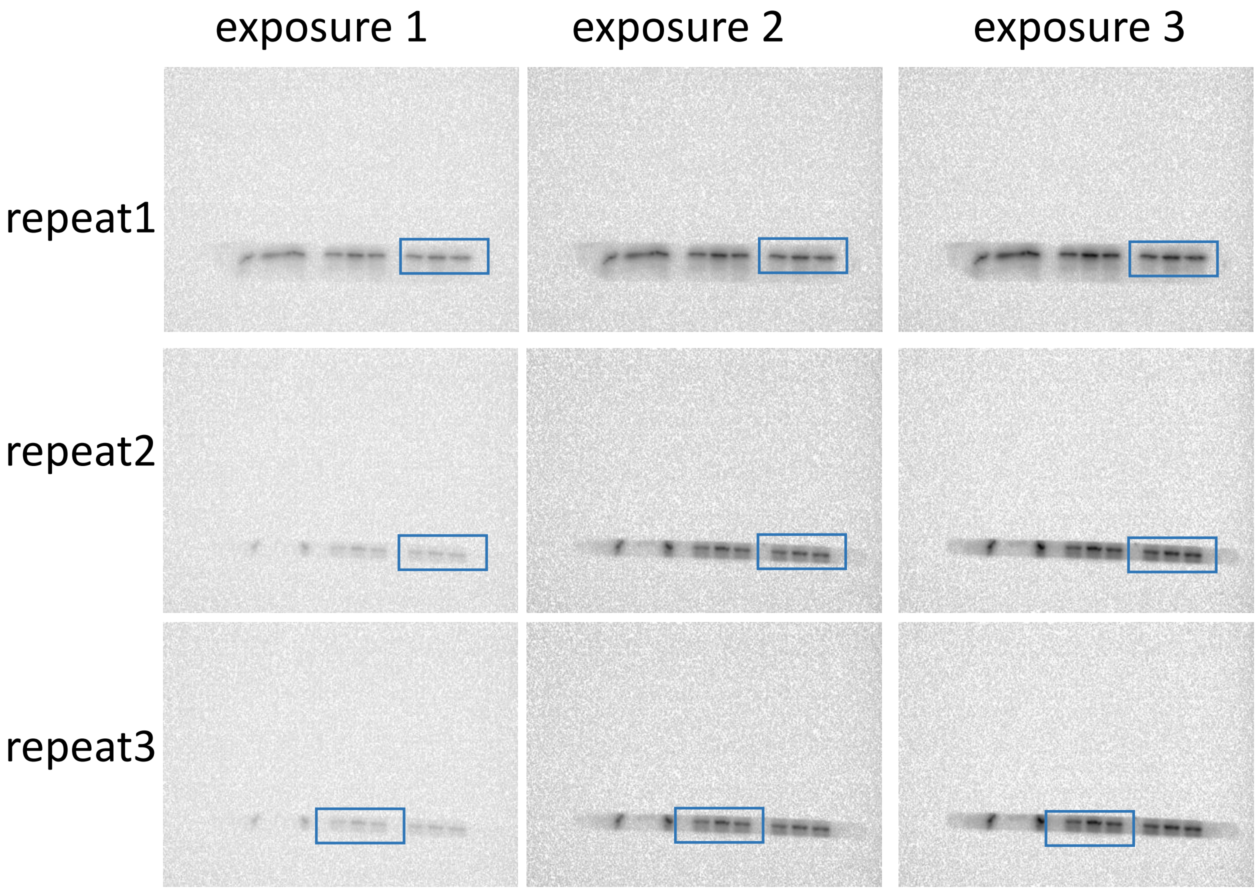

Figure 7

Figure 7a SKOV3
Atg5

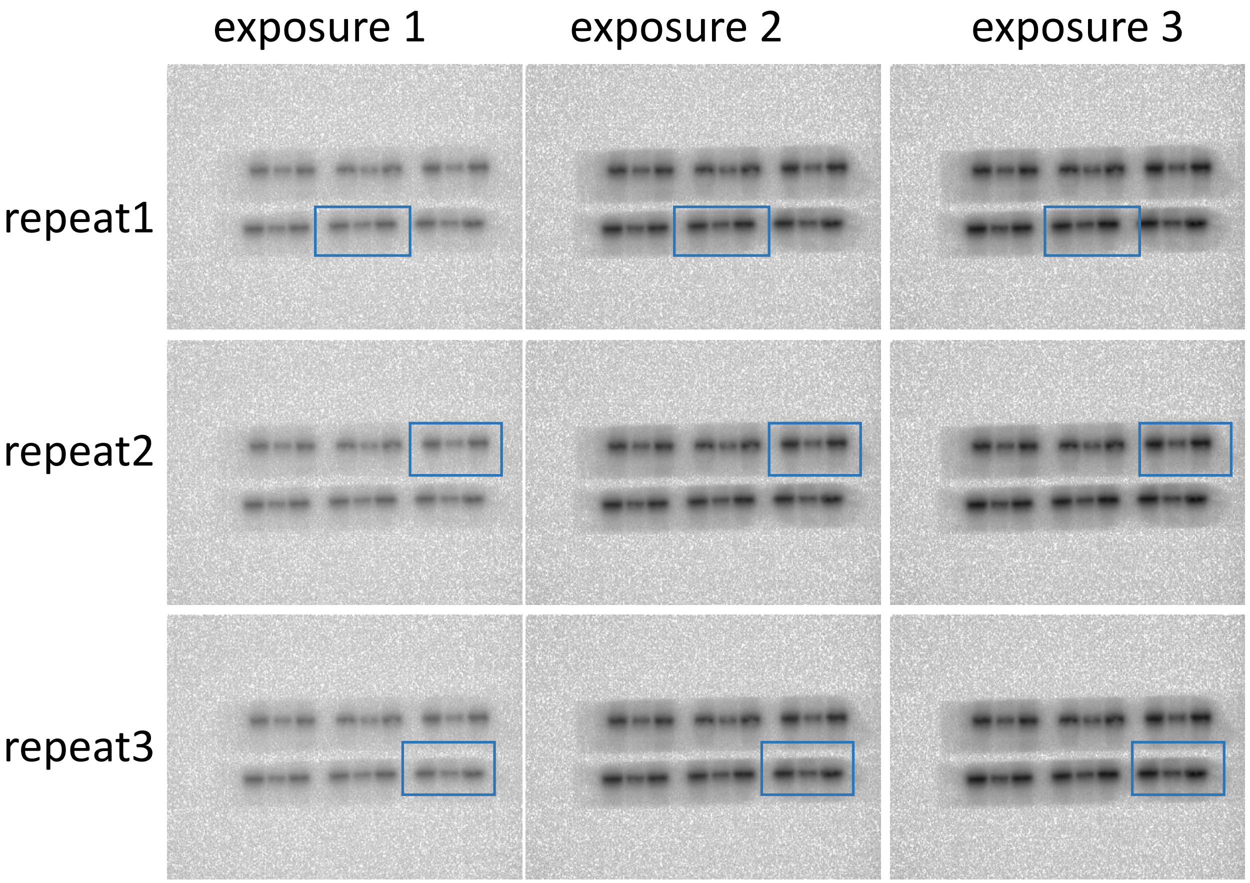

Figure 7

Figure 7a SKOV3
Beclin1

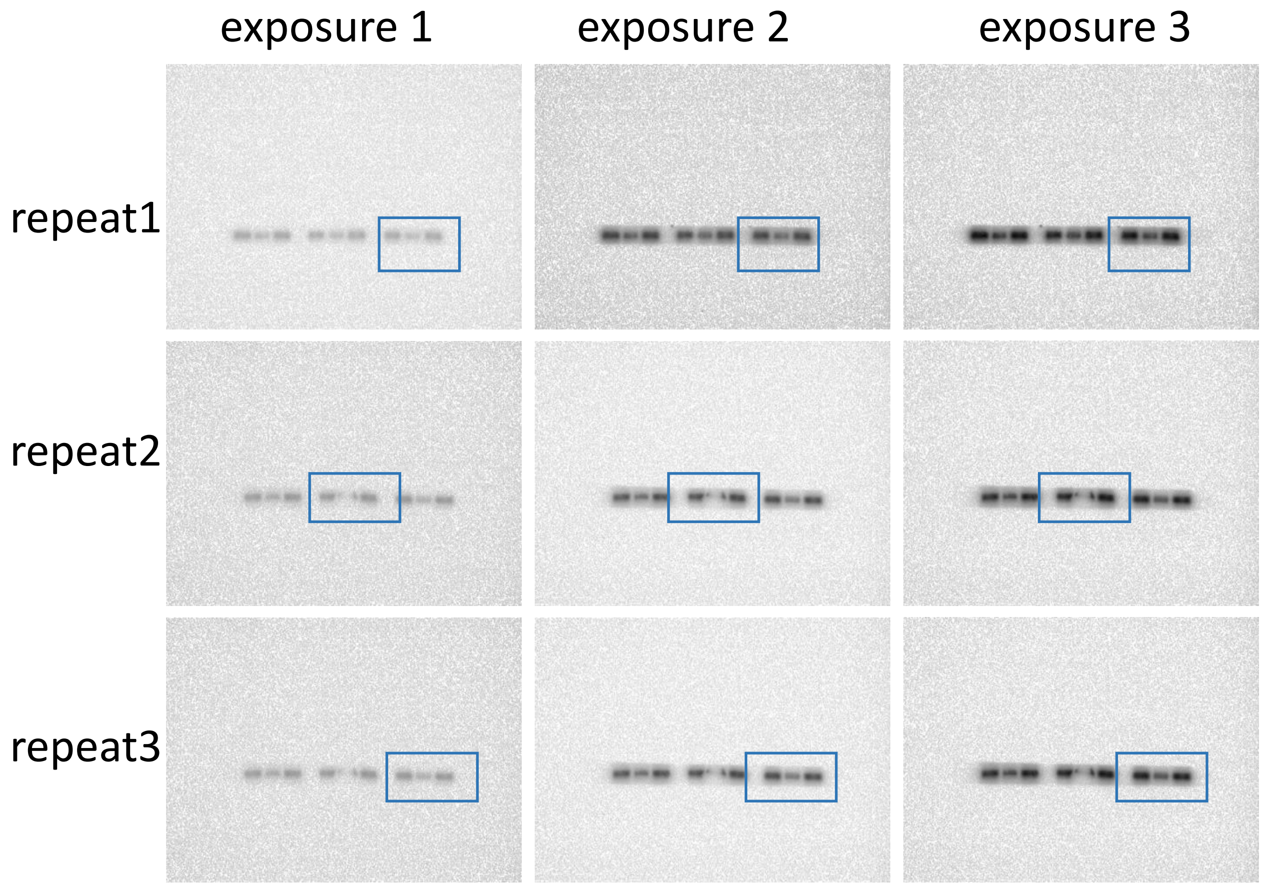

Figure 7

Figure 7a SKOV3
LC3

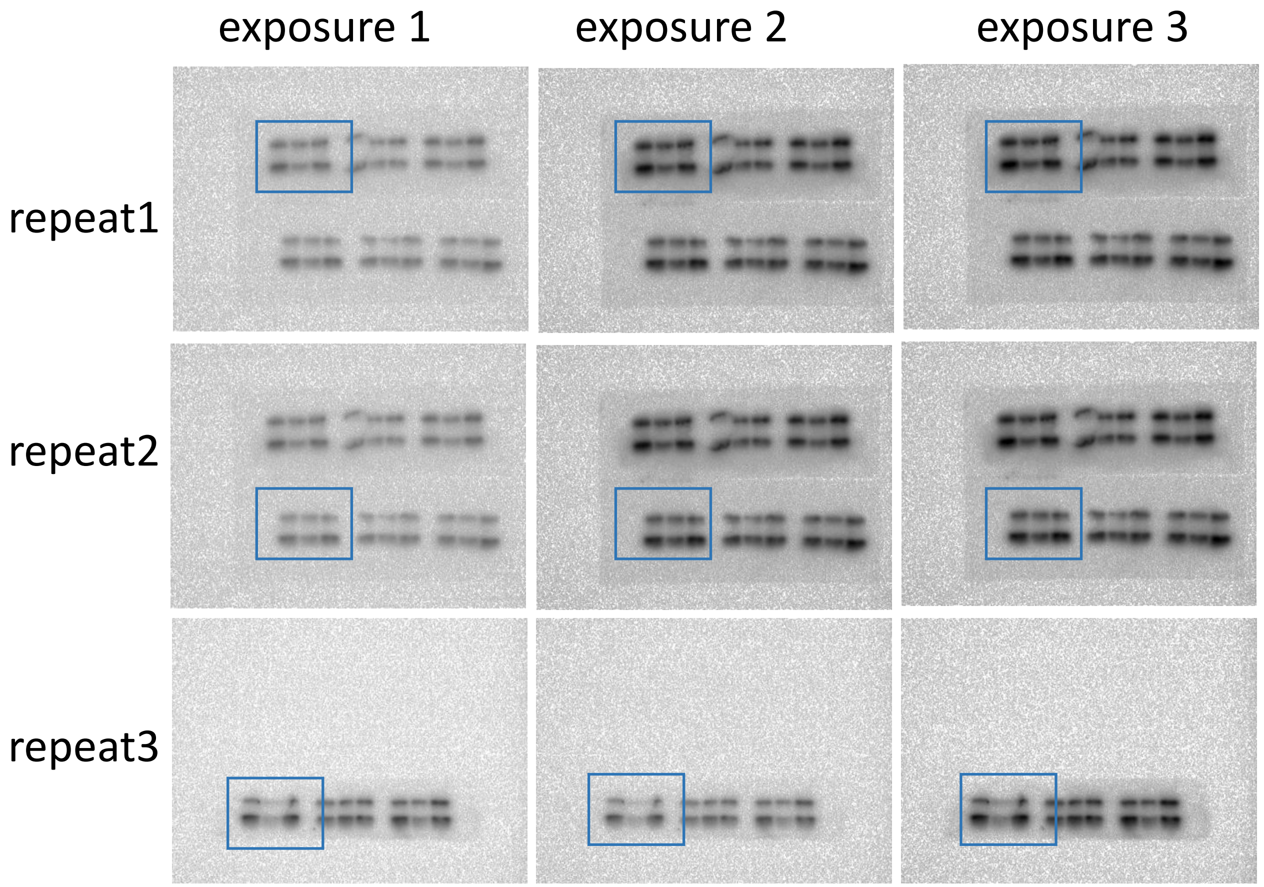

Figure 7

Figure 7a SKOV3
NCA

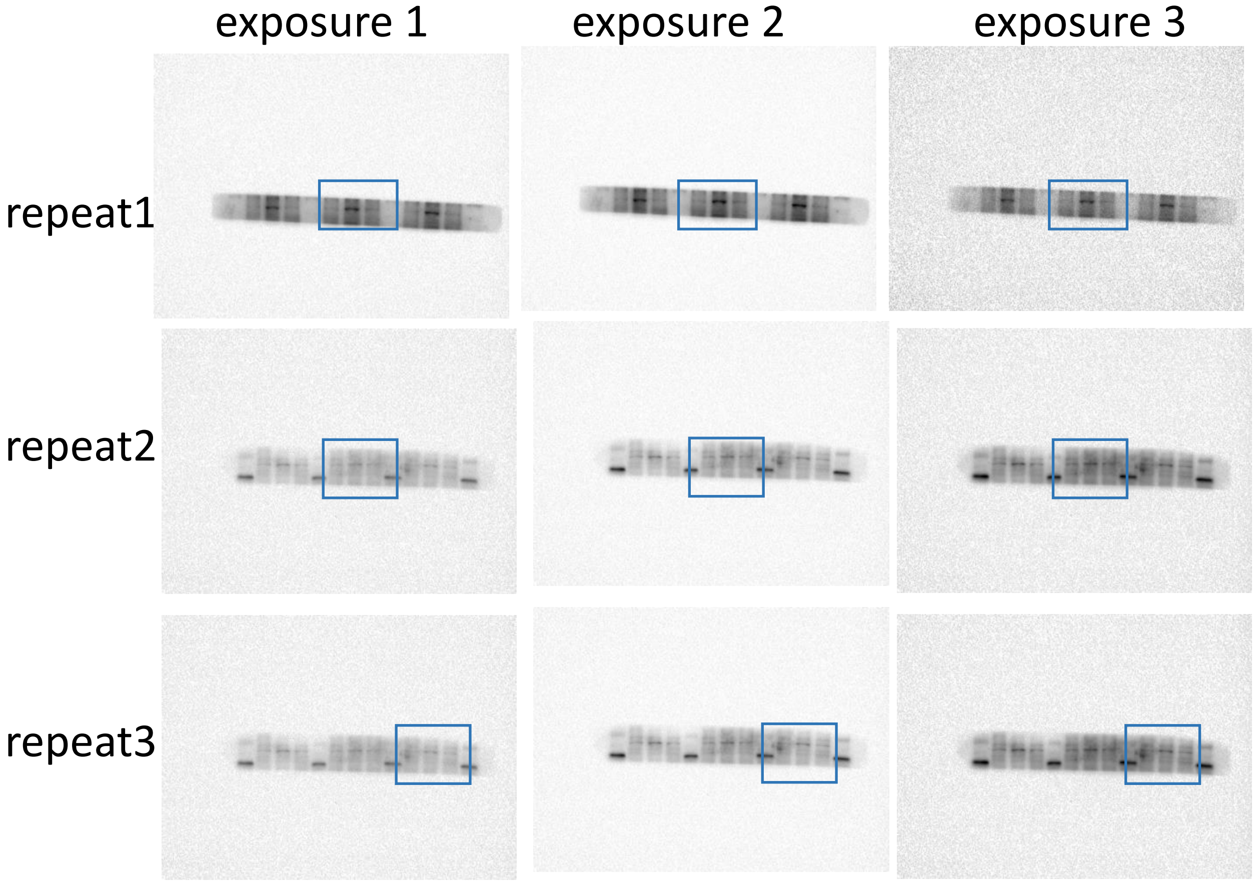

Figure 7

Figure 7a SKOV3
ECA

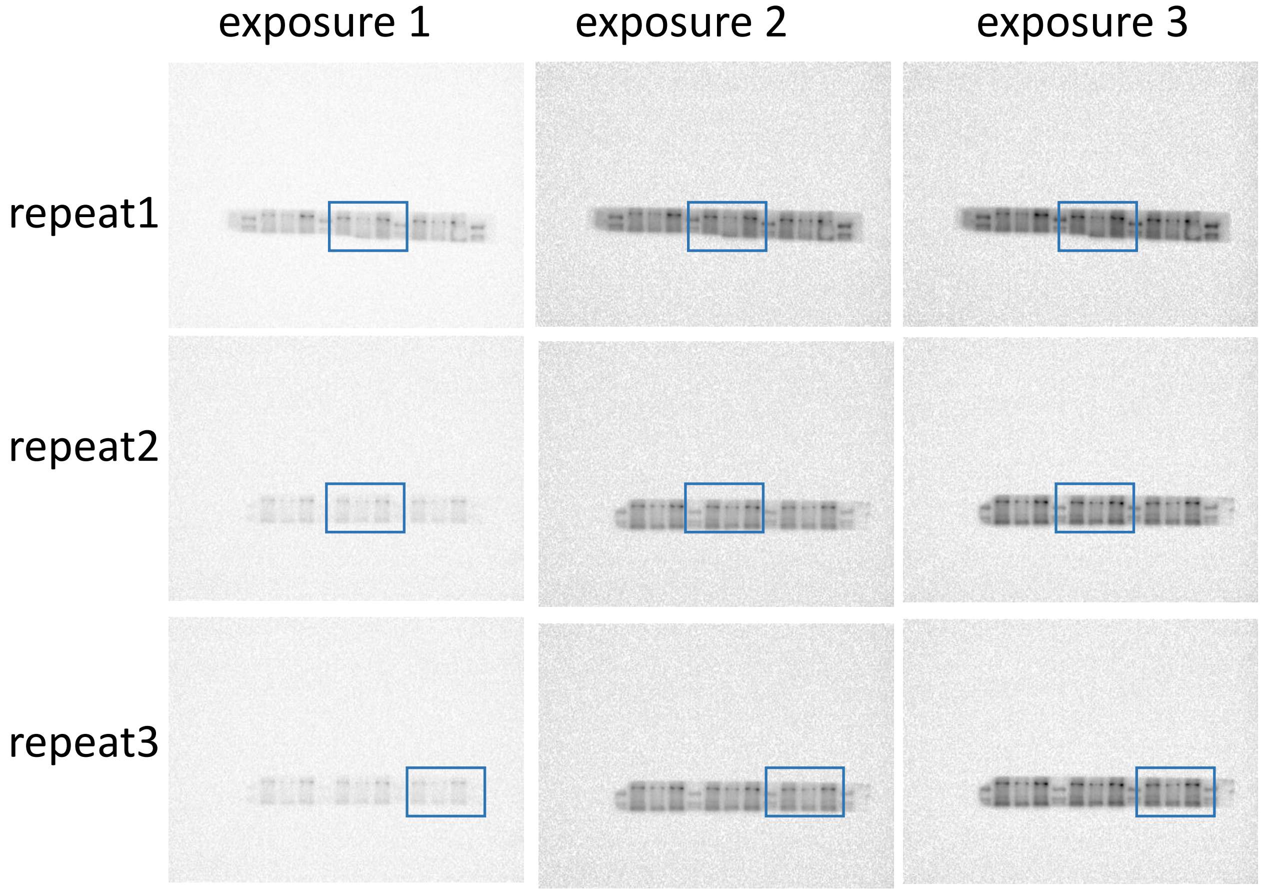

Figure 7

Figure 7a SKOV3

Timp2

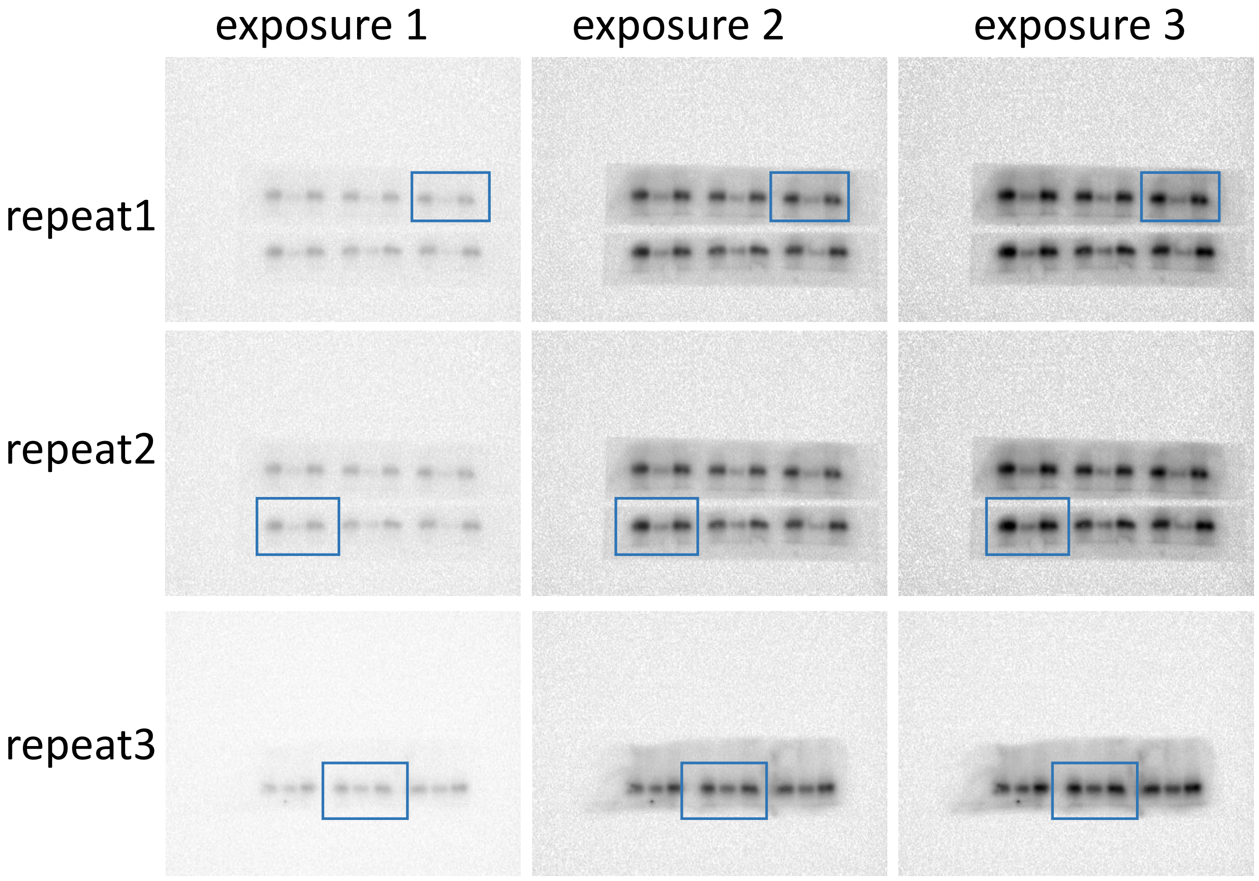

Figure 7

Figure 7a SKOV3

GRP94

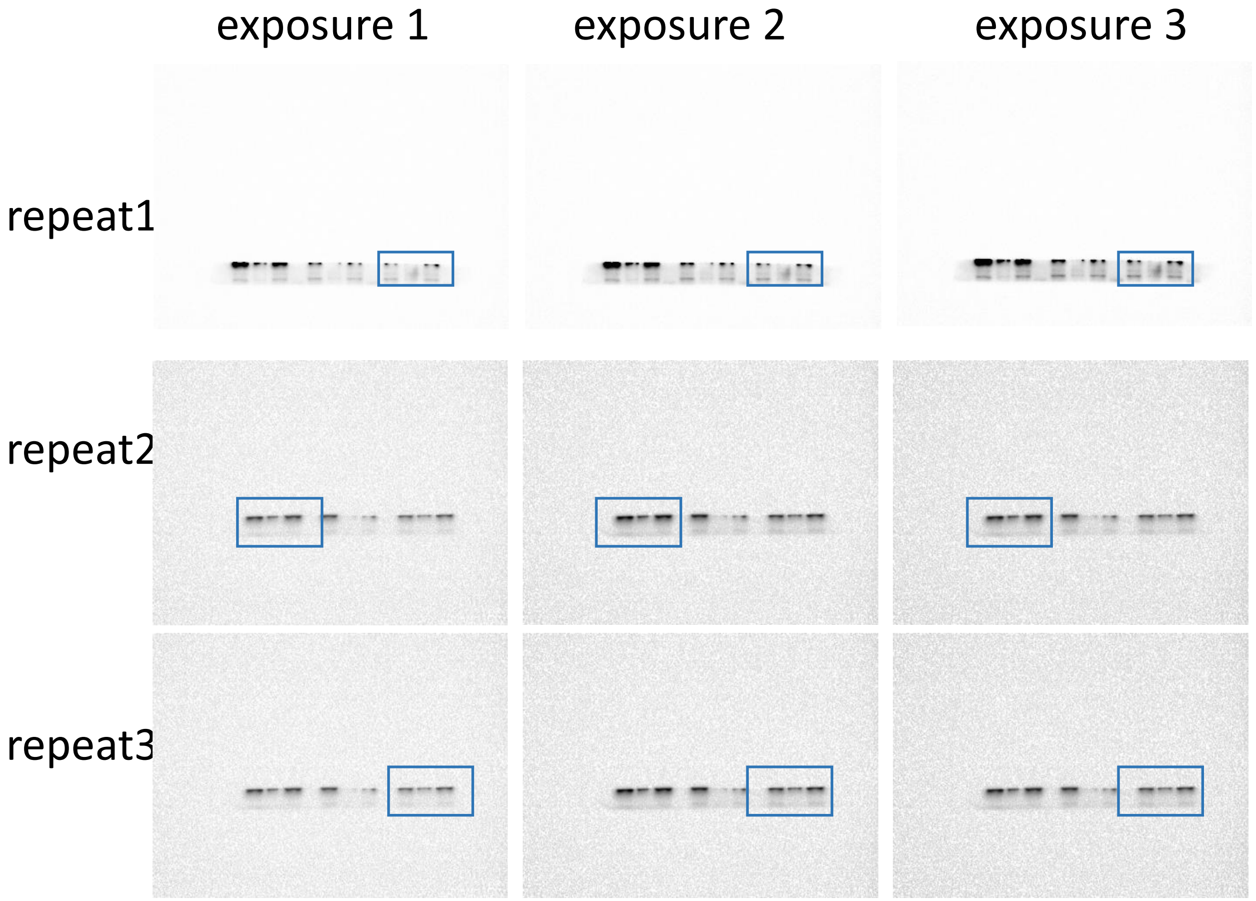

Figure 7

Figure 7a SKOV3
GRP78

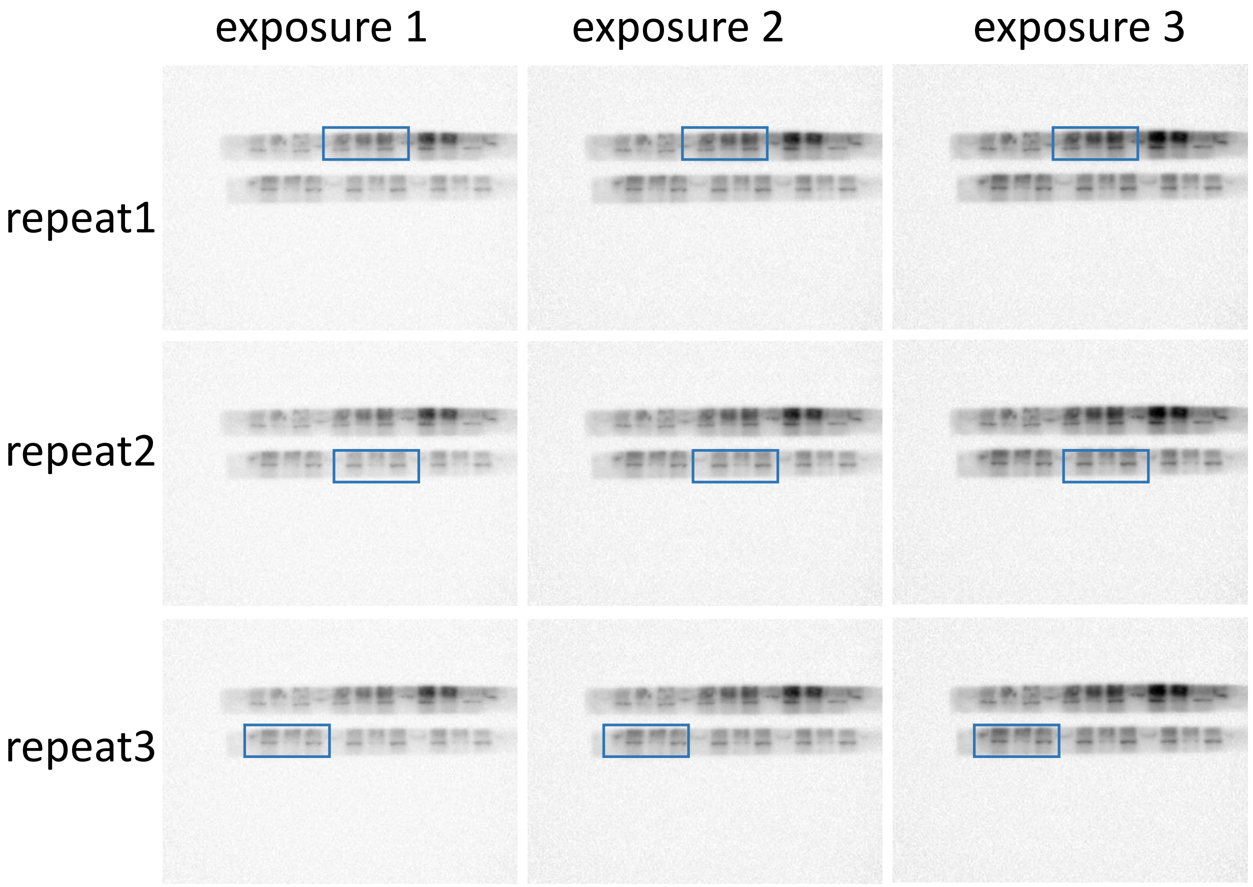

Figure 7

Figure 7a SKOV3
GAPDH

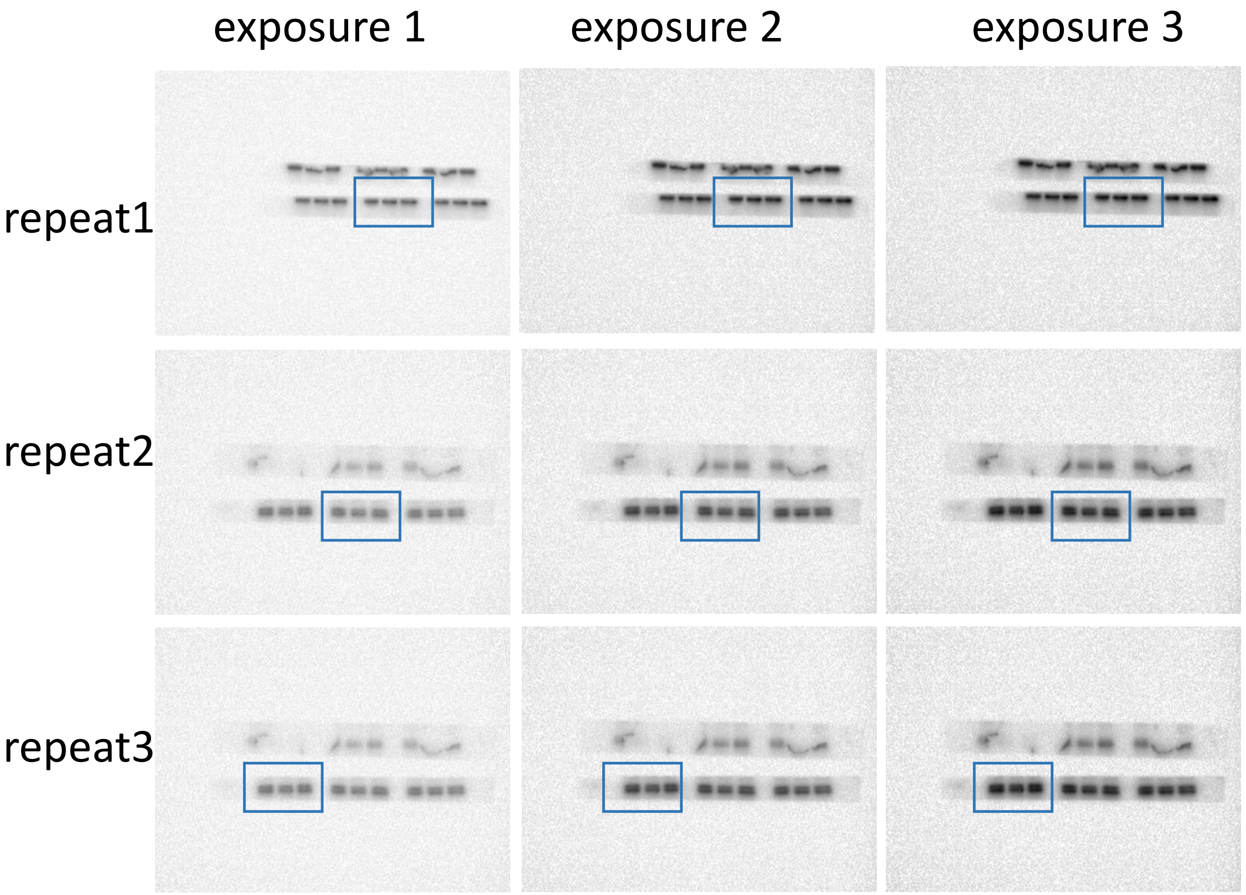

Figure 8

Figure 8a CAOV3

P-PI3K

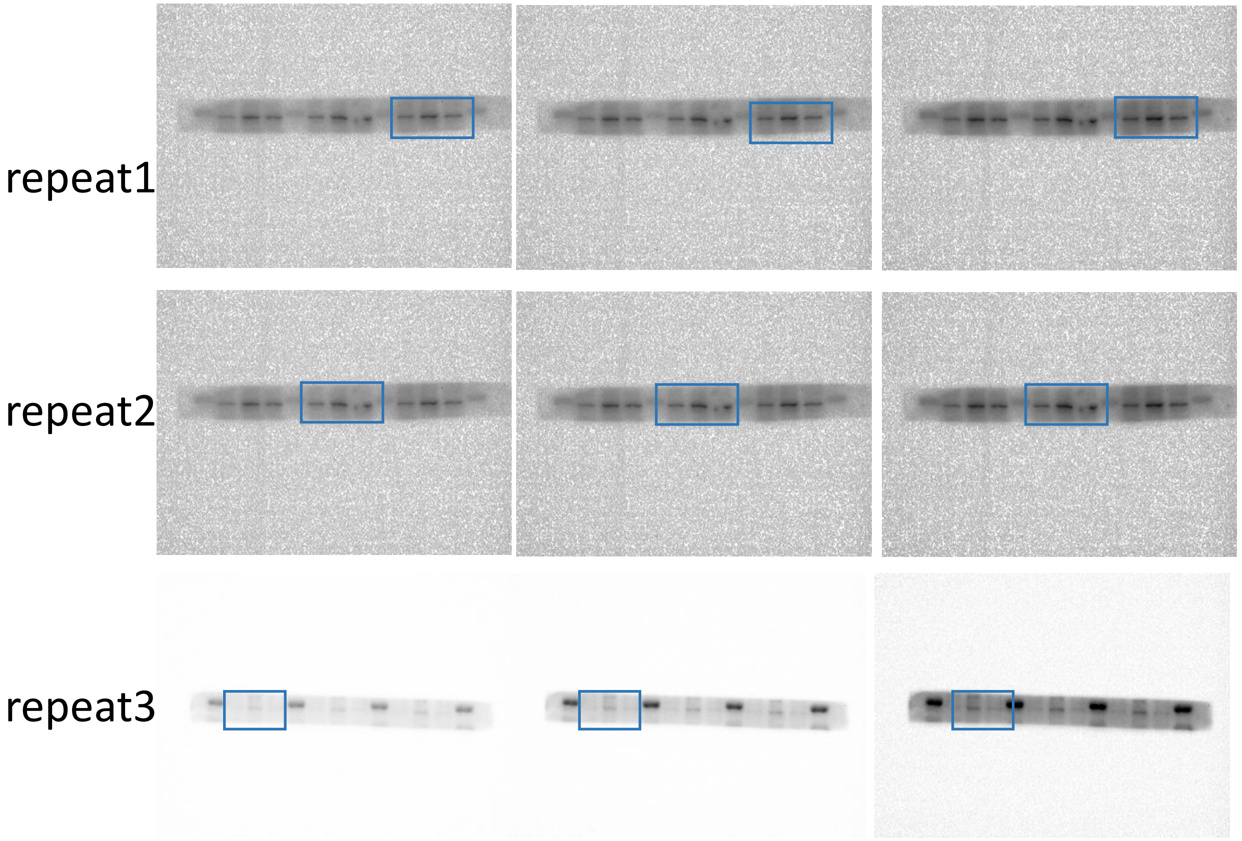

Figure 8

Figure 8a CAOV3

PI3K

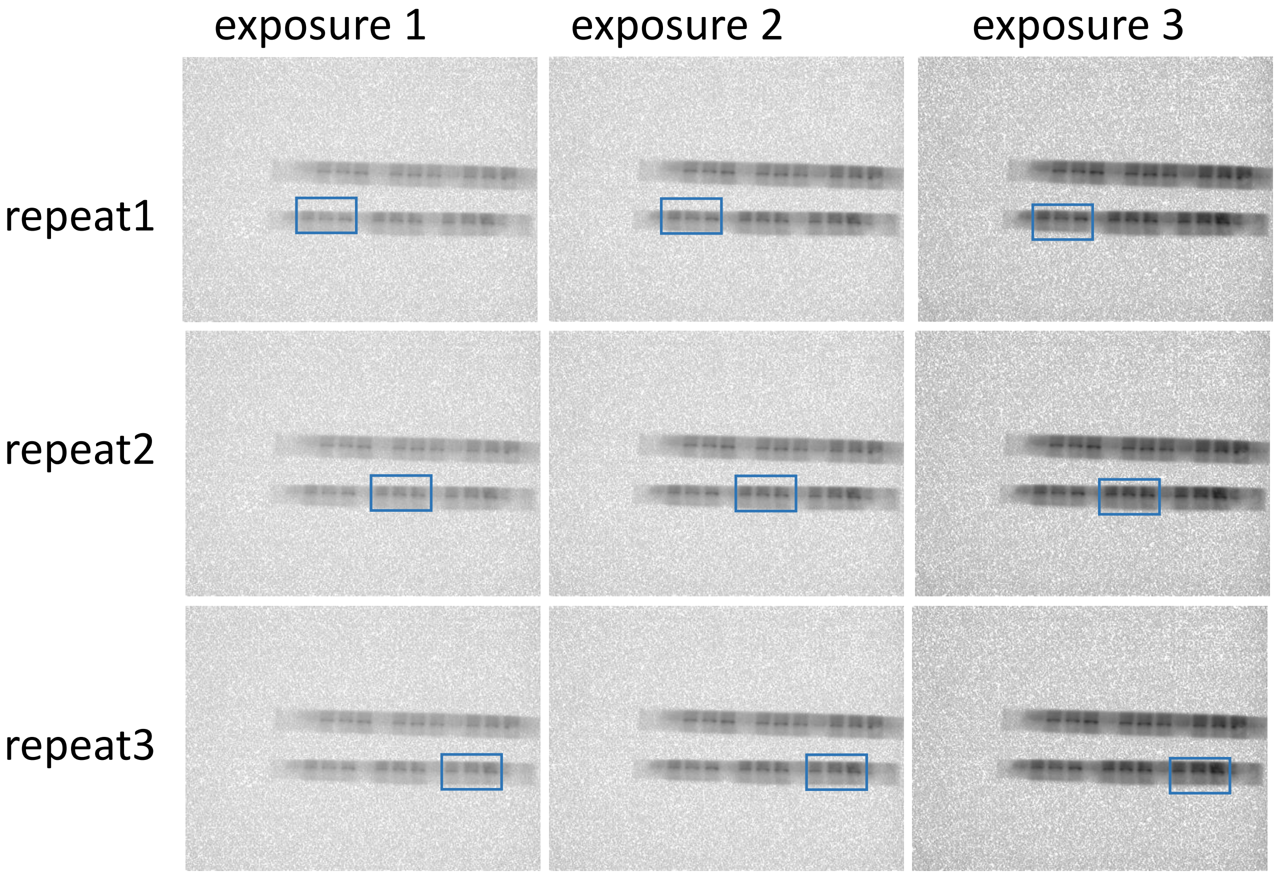

Figure 8

Figure 8a CAOV3
p-AKT

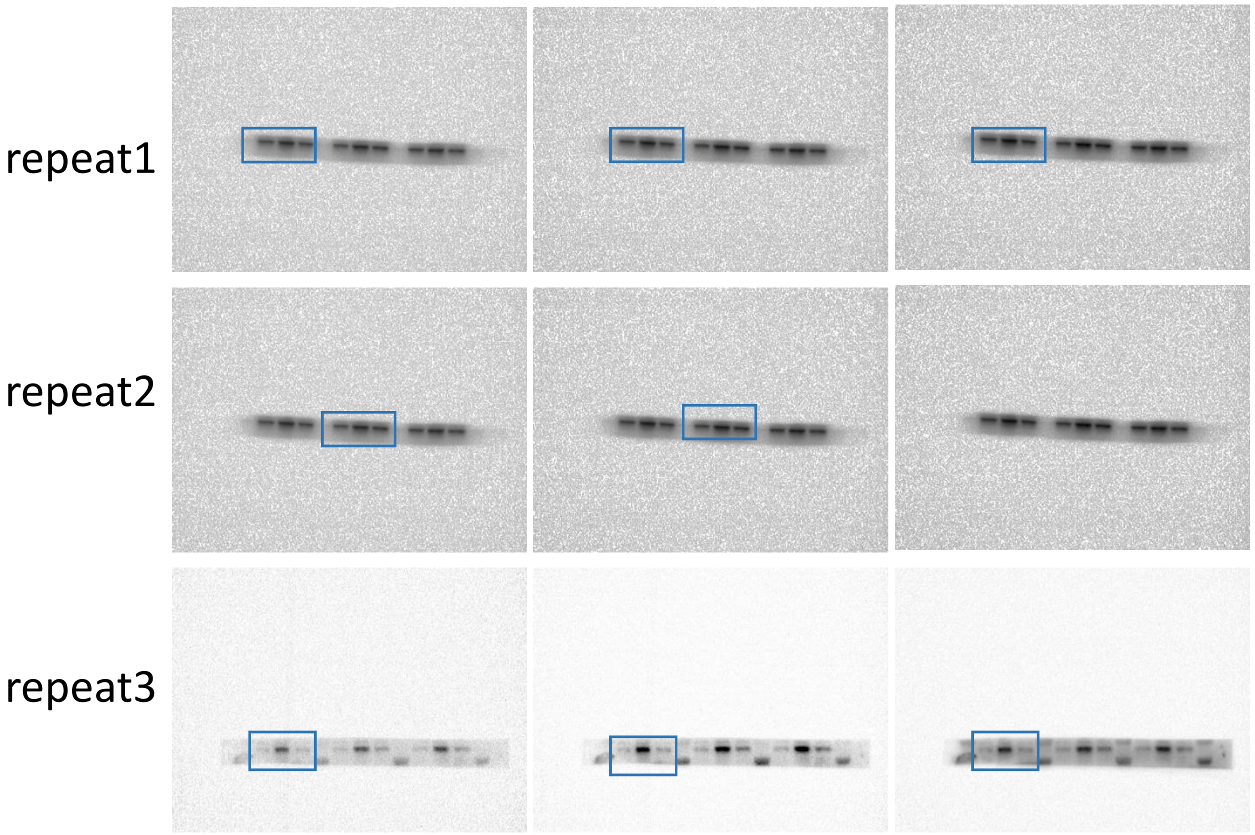

Figure 8

Figure 8a CAOV3
AKT

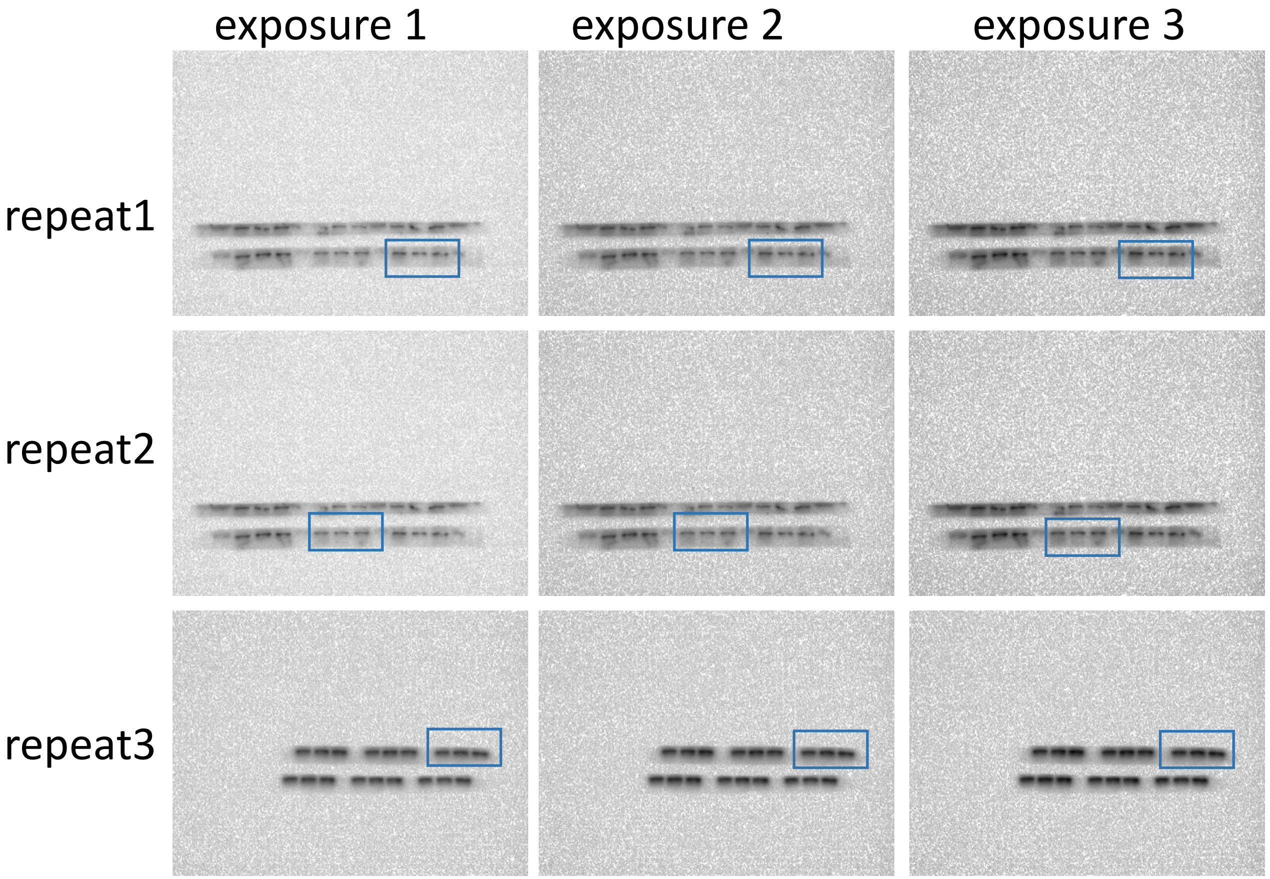

Figure 8

Figure 8a CAOV3

p-mTOR

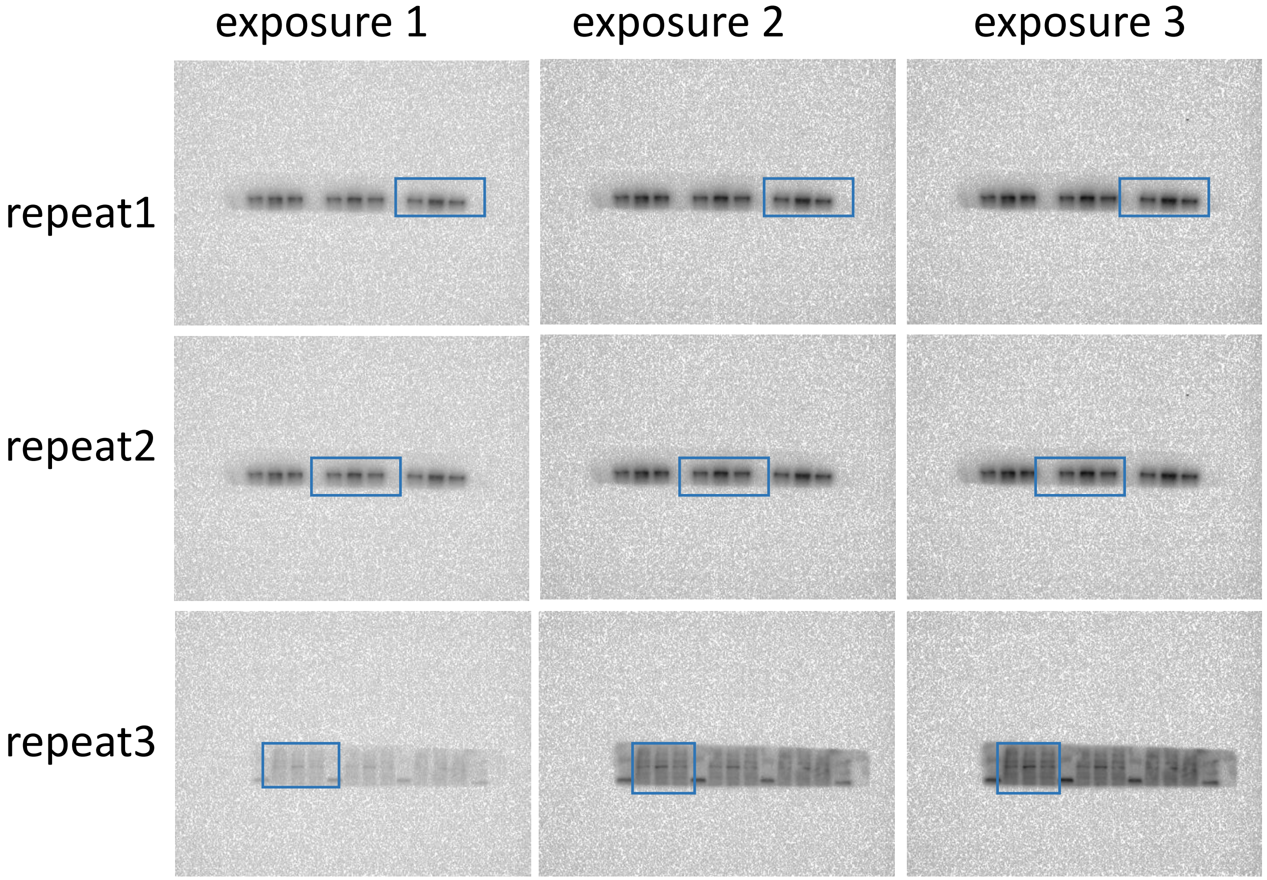

Figure 8

Figure 8a CAOV3

mTOR

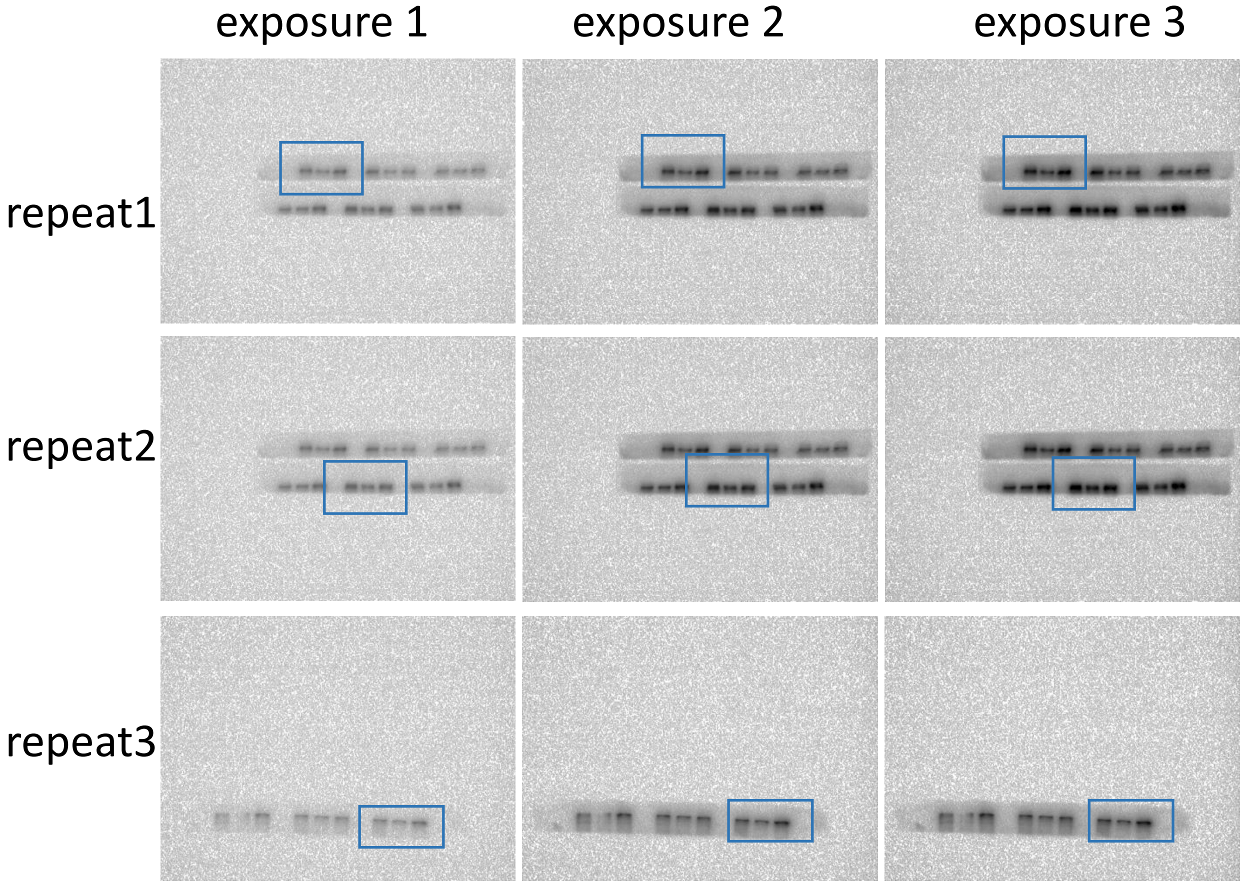

Figure 8

Figure 8a CAOV3
p-P38

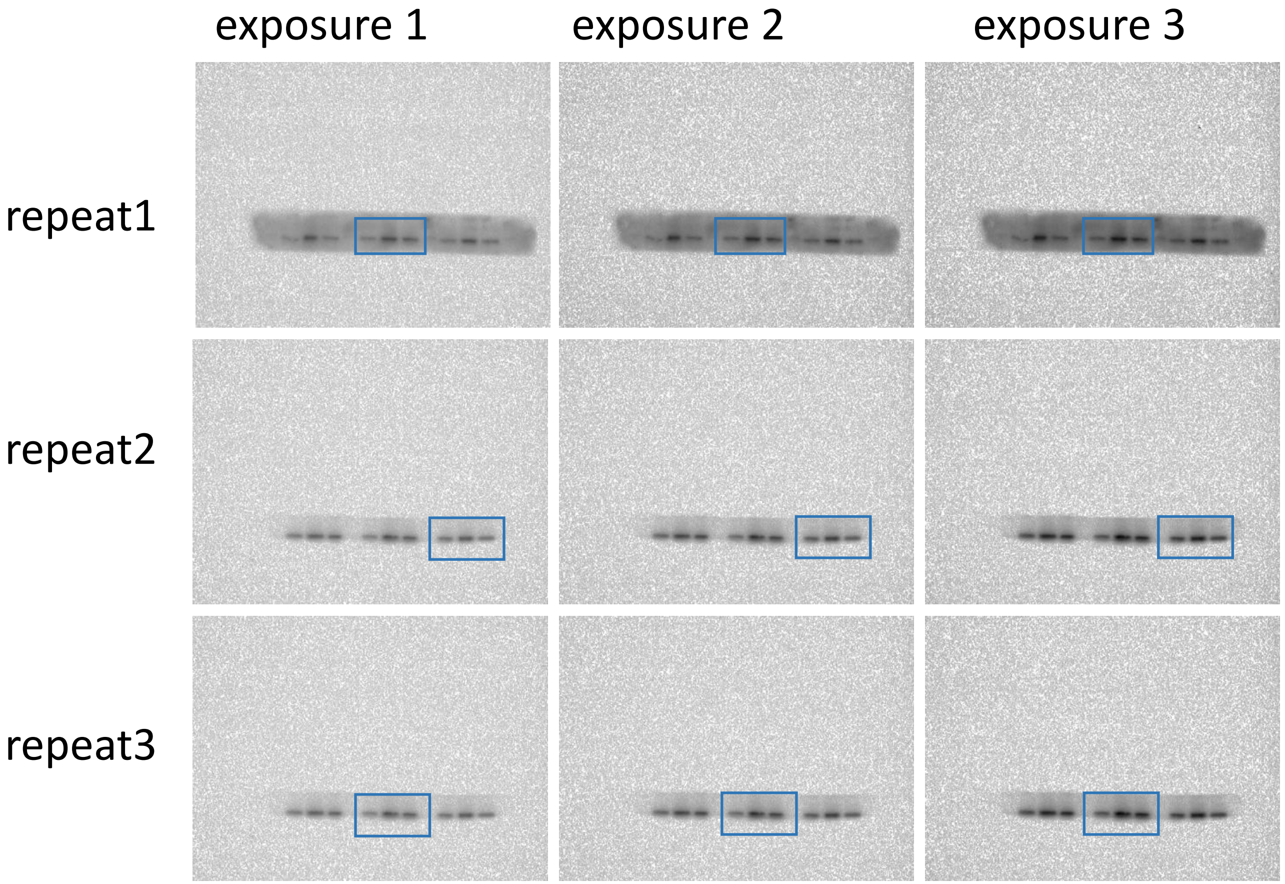

Figure 8

Figure 8a CAOV3
P38

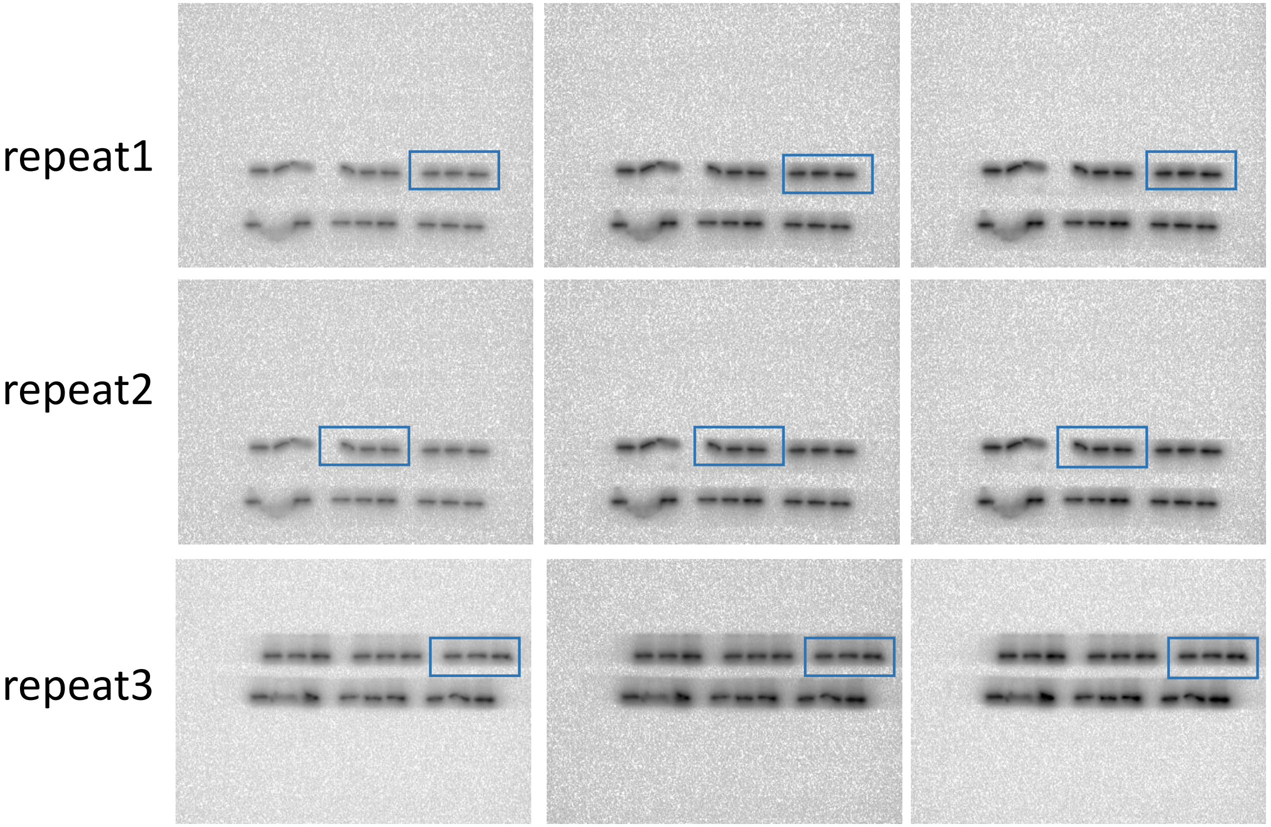

Figure 8

Figure 8a CAOV3

GAPDH

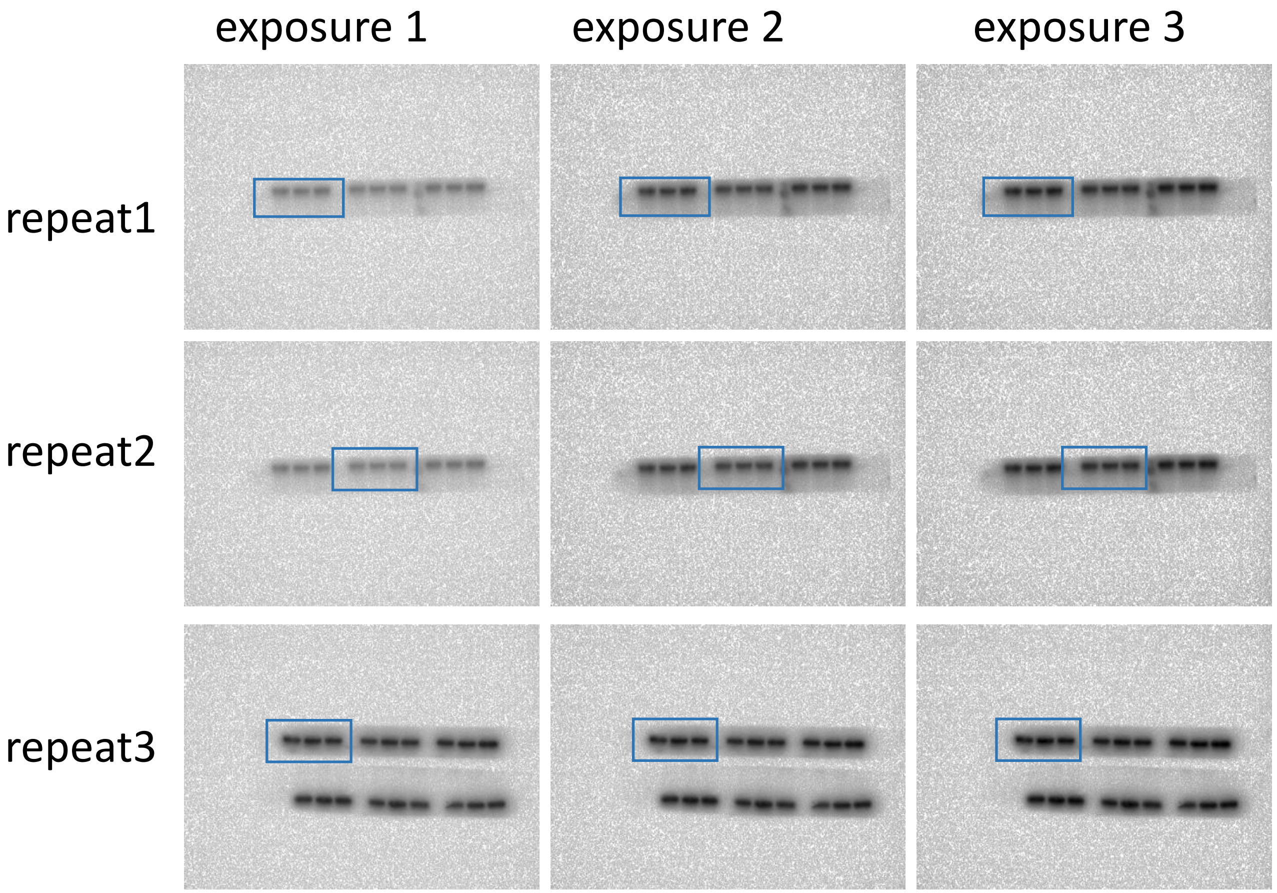

Figure 8

Figure 8a SKOV3

P-PI3K

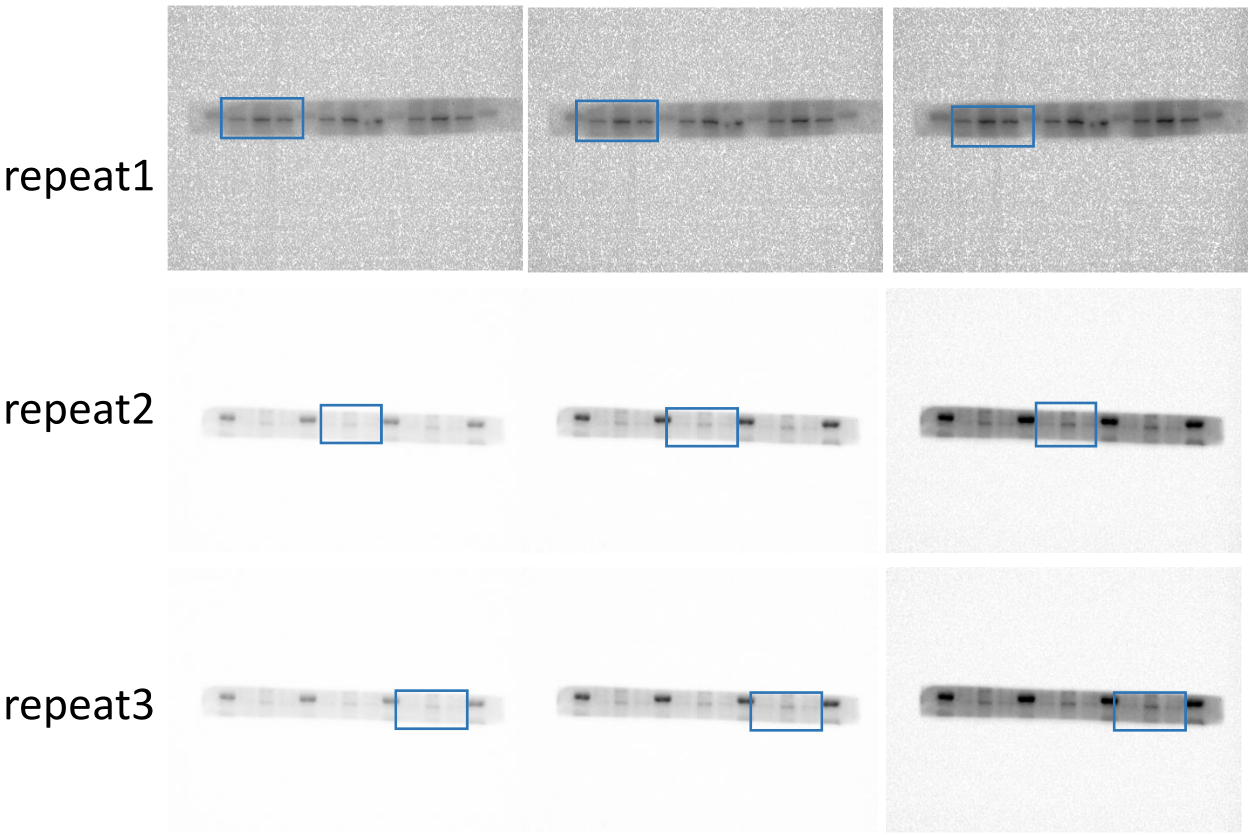

Figure 8

Figure 8a SKOV3
PI3K

exposure 1 exposure 2 exposure 3

repeat1
repeat2
repeat3

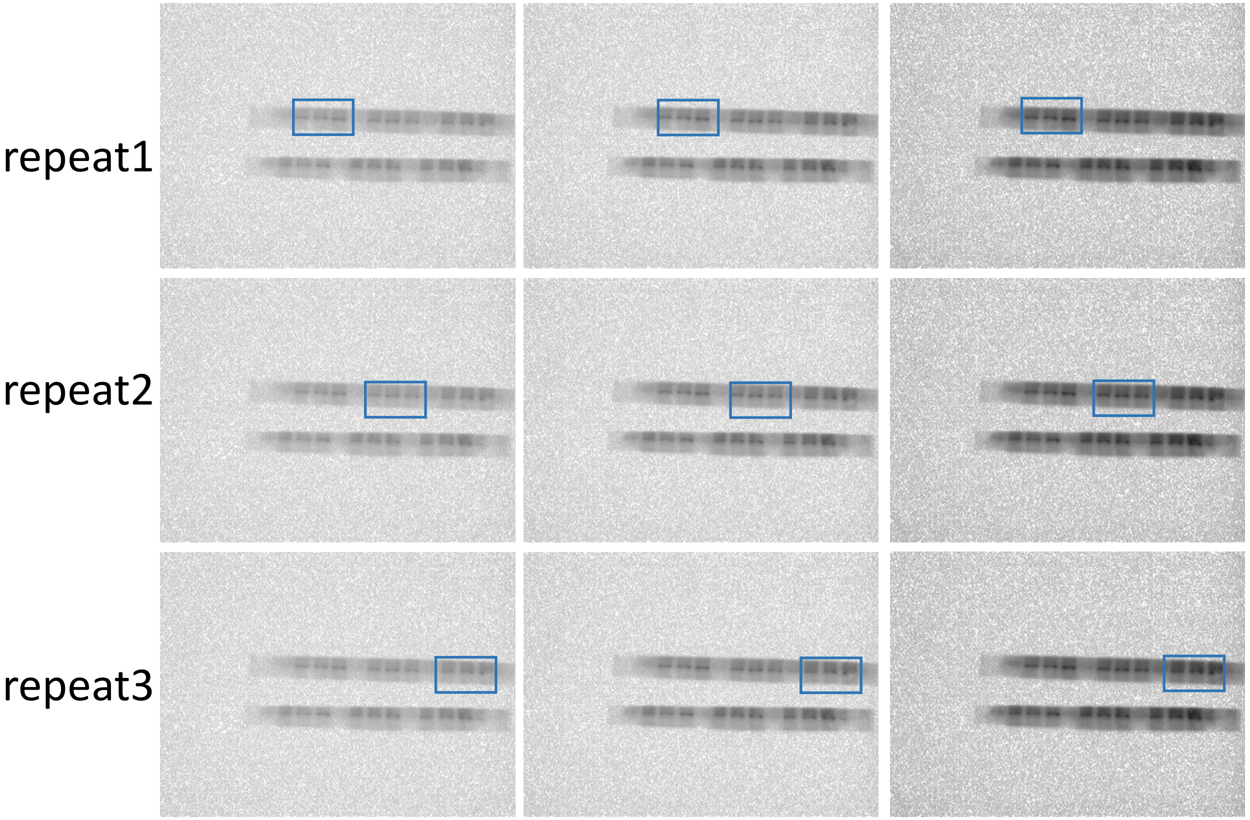

Figure 8

Figure 8a SKOV3
p-AKT

exposure 1 exposure 2 exposure 3

repeat1
repeat2
repeat3

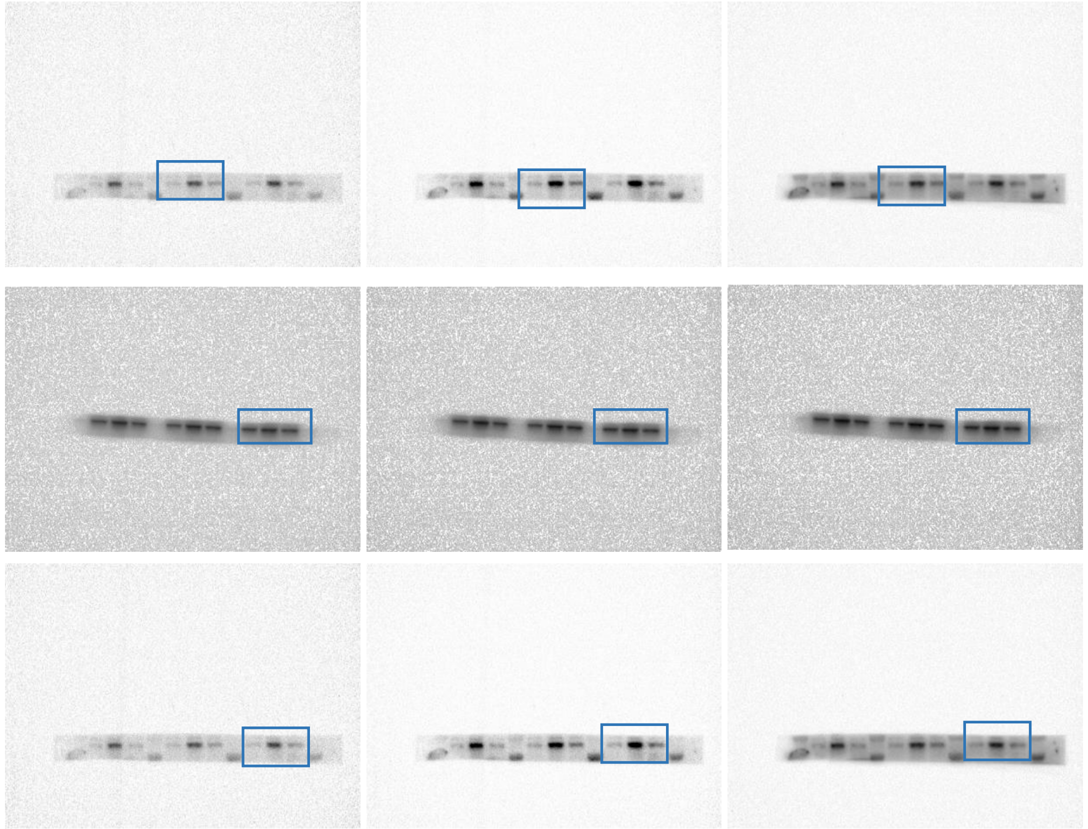

Figure 8

Figure 8a SKOV3
AKT

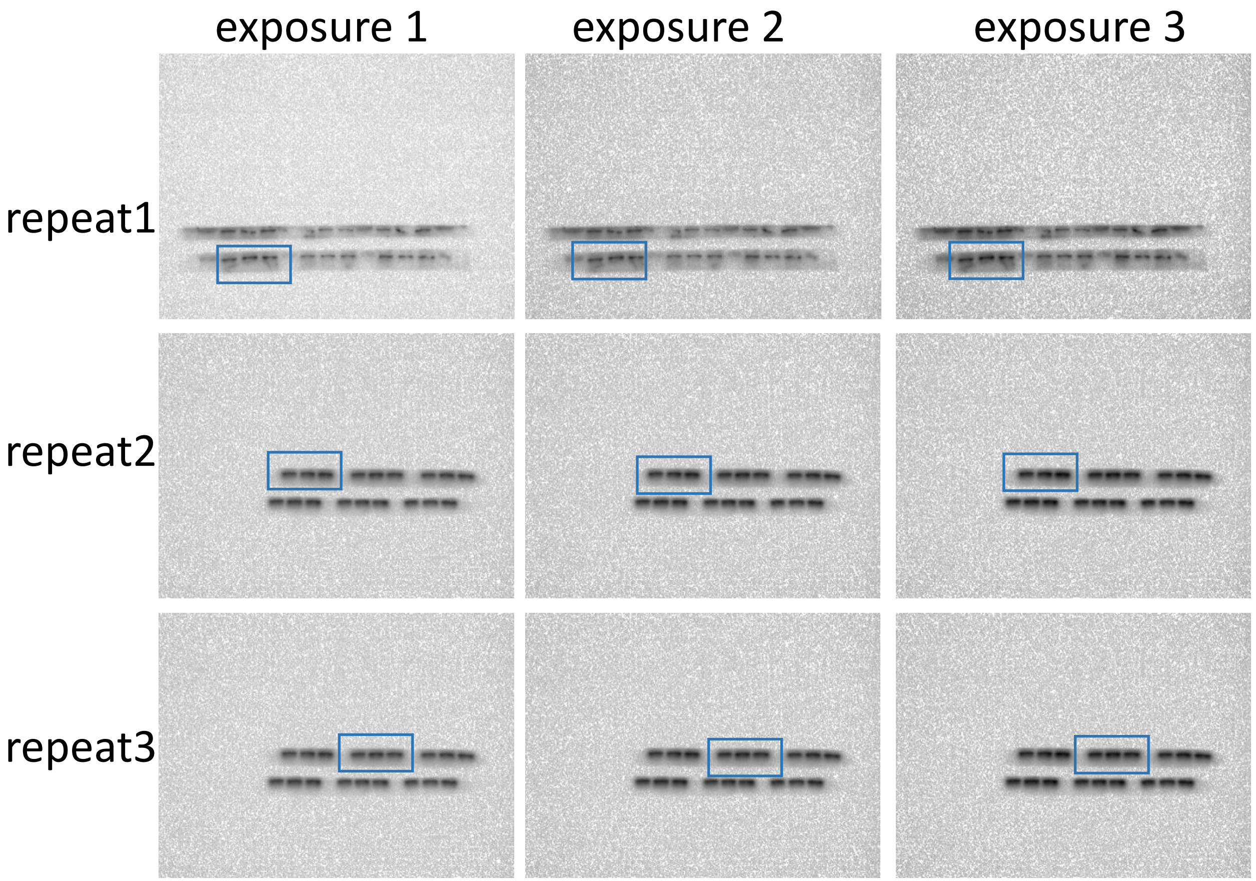

Figure 8

Figure 8a SKOV3
p-mTOR

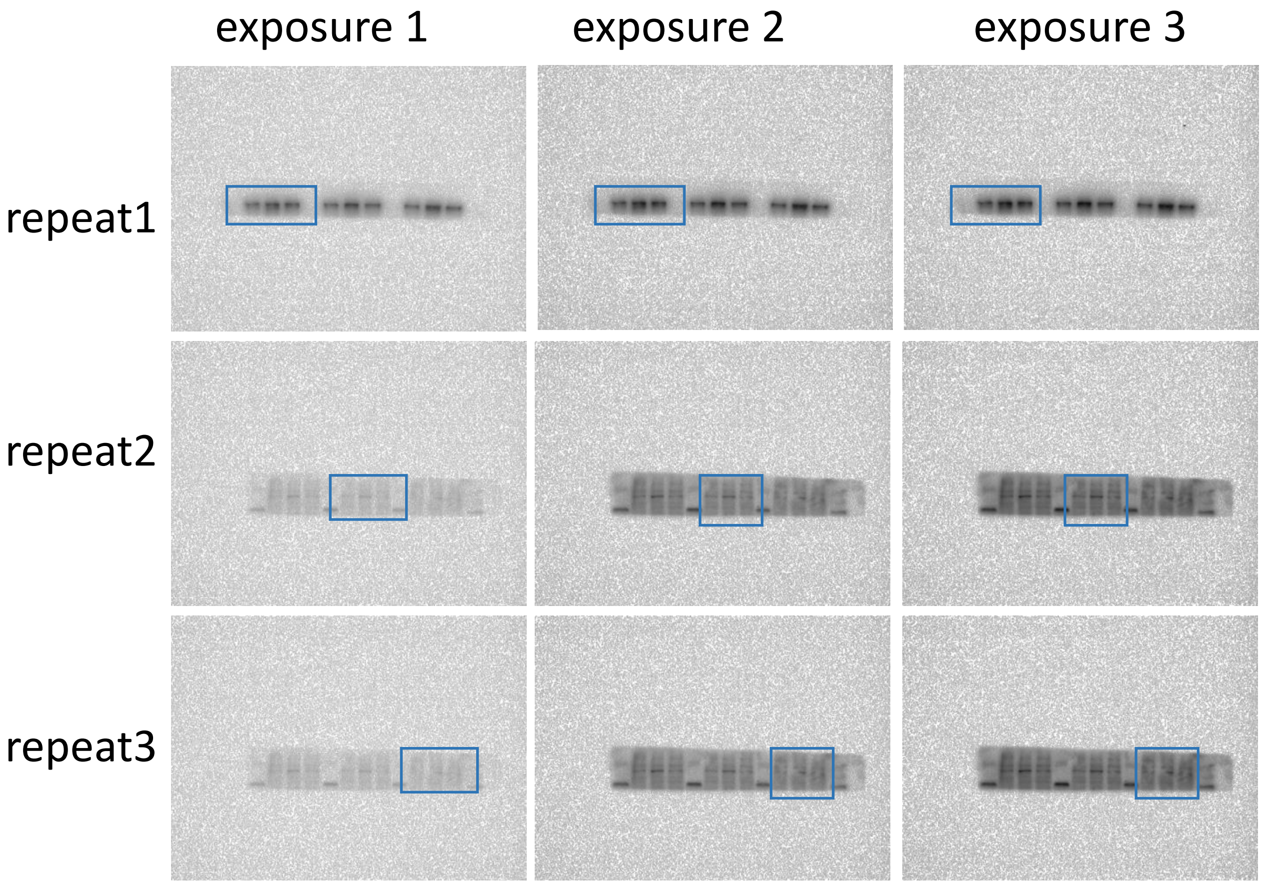

Figure 8

Figure 8a SKOV3
mTOR

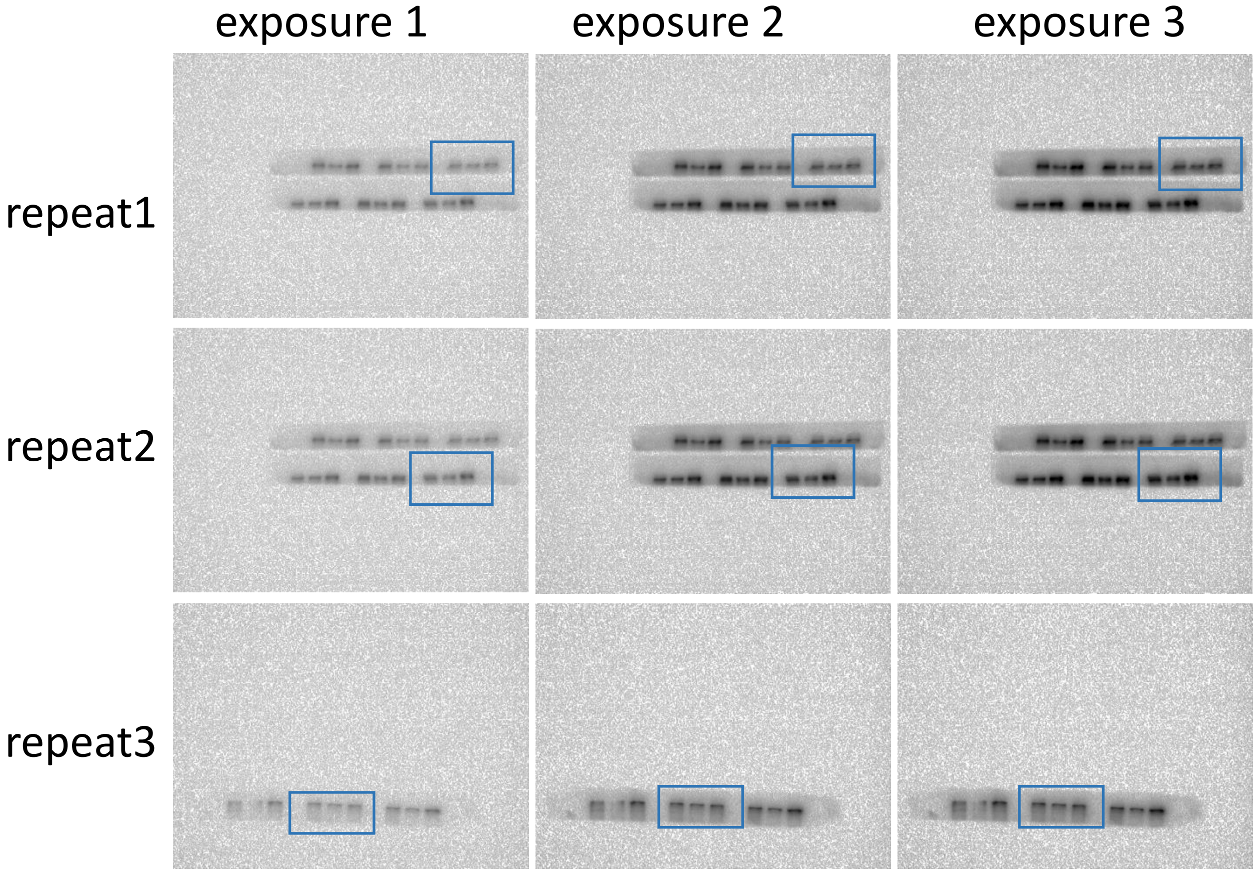

Figure 8

Figure 8a SKOV3
p-P38

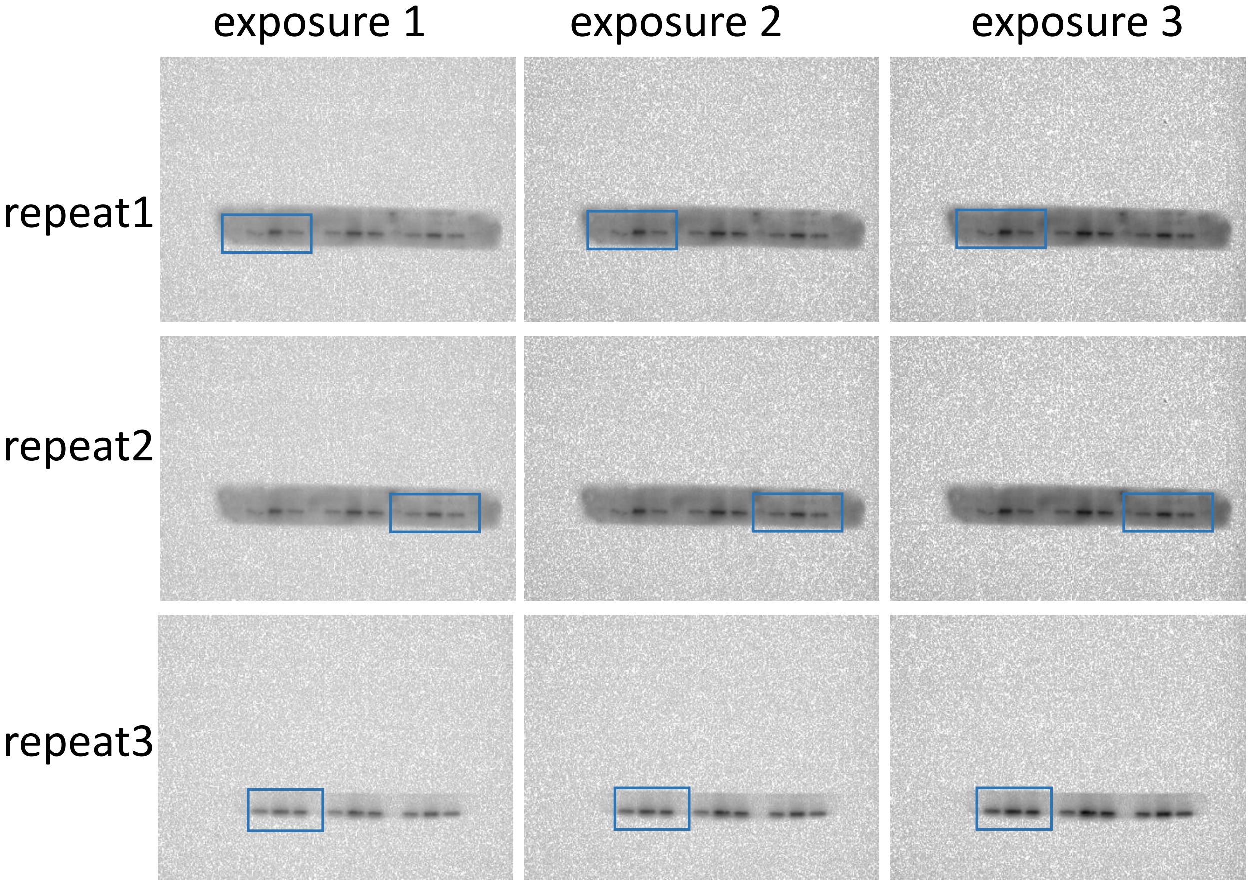

Figure 8

Figure 8a SKOV3
P38

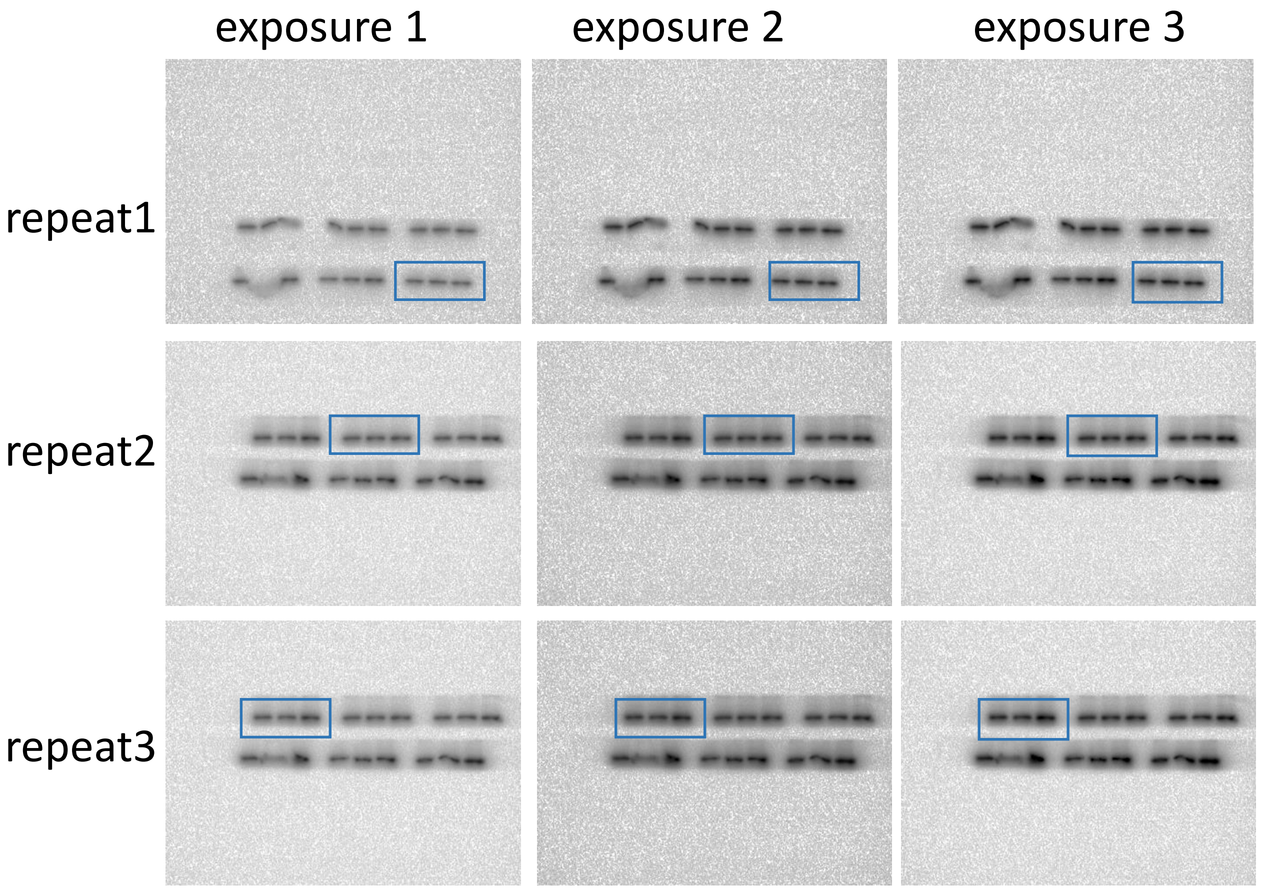

Figure 8

Figure 8a SKOV3
GAPDH

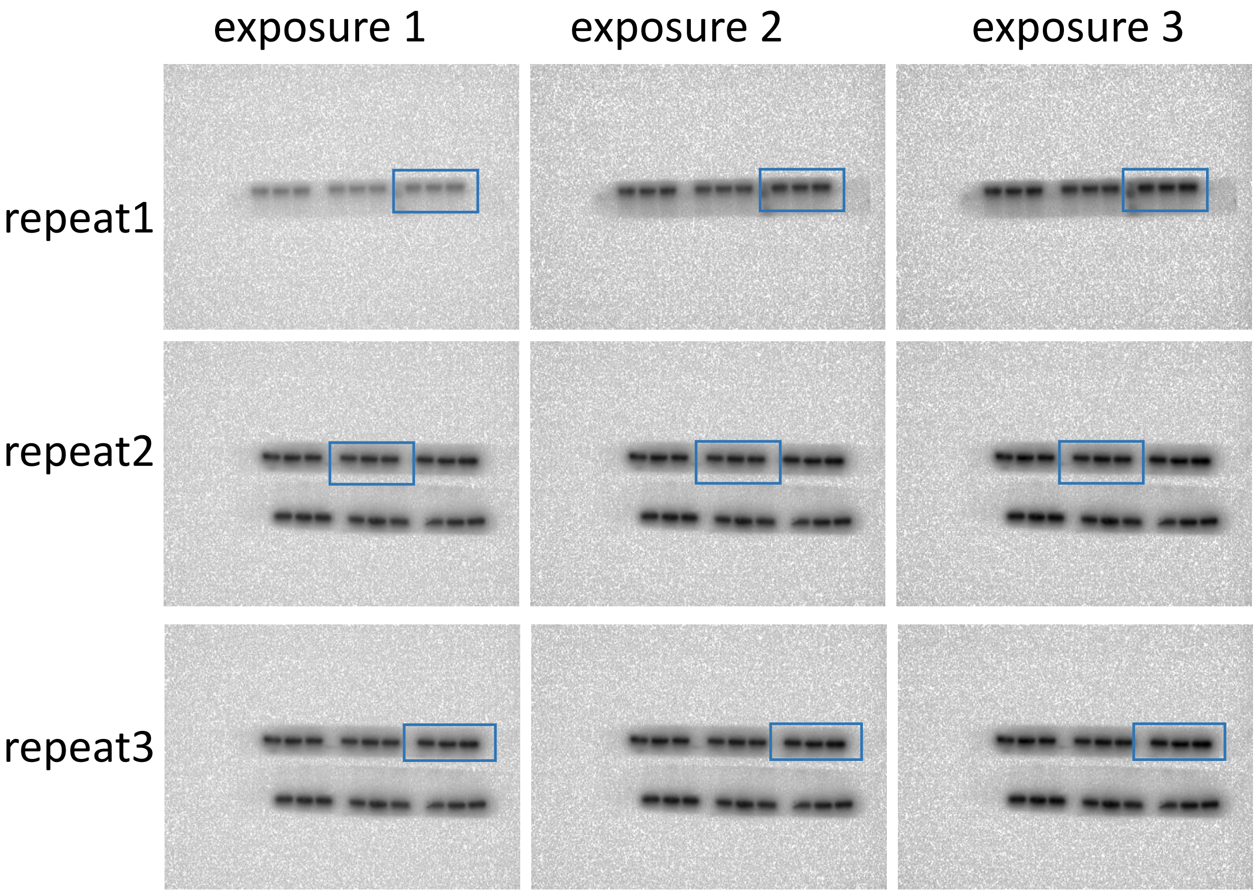

Supplement: Supplementary file 1 — Additional file 1. Supplementary Figures. [file 12885_2022_10248_MOESM1_ESM.pdf]
